# British Association for Cancer Research 22nd Annual general meeting. 13-15 April 1981. Abstracts.

**Published:** 1981-08

**Authors:** 


*Br. J. Cancer* (1981) **44**, 271

### BRITISH ASSOCIATION FOR CANCER RESEARCH
### 22nd ANNUAL GENERAL MEETING

*Held at the University of Keele 13–15 April 1981*

### ABSTRACTS OF MEMBERS' PROFFERED PAPERS

### CHLOROACETALDEHYDE, A VINYL CHLORIDE METABOLITE, INDUCES ERRORS DURING *IN VITRO* DNA SYNTHESIS.

J. A. Hall & R. Saffhill, *Paterson Laboratories, Christie Hospital and Holt Radium Institute, Manchester*

Chloroacetaldehyde (CA), a rearranged metabolic product of the human carcinogen vinyl chloride, has been shown to be mutagenic in certain microbial systems as well as towards mammalian cells. CA has been reacted with the alternating DNA-like polymers poly(dA-dT) and poly(dC-dG) when, as with DNA, etheno-adducts of the adenine and cytosine bases are formed. These treated polymers, when used as templates for *E. coli* DNA polymerase I, show a decreased ability to direct DNA synthesis. This is accompanied by an increase in the relative levels of non-complementary nucleotides incorporated into the newly synthesised DNA-like material. This increased with the amount of modified base present in the templates used. With the poly(dA-dT) templates one dGMP residue was incorporated for every $60\pm$ etheno-adenine residues present, whilst no dCMP misincorporation was detected. One misincorporation of dAMP or dTMP occurred in the presence of $30\pm$ and $80\pm$ ethenocytosine residues respectively in the poly(dC-dG) templates. For the modified poly(dC-dG) templates, a nearest-neighbour analysis shows that the majority of the errors were incorporated opposite the cytosine (or modified cytosine) bases. Extensive analyses of the modifications present in the templates indicate that the misincorporations observed are not due to the presence of apurinic sites or to the formation of uracil or xanthine by deamination of the cytosine or adenine bases respectively. We conclude that the non-complementary nucleotide incorporations probably arise from the presence of etheno-adducts in the templates used.

### ALKYLATION, PERSISTENCE OF DNA LESIONS, CELL SURVIVAL AND MUTATION IN 4 RODENT TUMOUR CELL LINES AFTER EXPOSURE TO N-METHYL-N-NITROSOUREA (MNU).

L. Durrant, G. P. Margison & J. M. Boyle, *Paterson Laboratories, Christie Hospital and Holt Radium Institute, Manchester*

Dose response curves for colony forming ability were determined following incubation with MNU for 30 min at 37°C. The $D_0$ values were 0·7, 0·3, 0·4 and 0·07 mM MNU for Chinese hamster cell lines V79 and V79/79, rat Yoshida sarcoma (YS), and mouse leukaemia L1210 cells respectively. Induced mutation frequencies for resistance to 6-thioguanine were 13·8, 16·3 and $17{\cdot}0\times10^{-4}$ per mM MNU for V79, V79/79 and L1210 respectively. The methylated purines $O^6$-methylguanine ($O^6$-meG), N7-methylguanine (N7-meG) and 3-methyladenine (3-meA) were determined on DNA hydrolysates extracted at various times after MNU exposure. There was good correlation between cell survival and total methylated purines for all cell lines except L1210, which showed excessive killing. The half-lives of N7-meG and 3-meA varied among the cell lines, from 15–30 h and 3·6–7·2 h respectively. The half-life of $O^6$-meG was $\geqslant$100 h, 21 h, 5·7 h and 49·5 h in V79, V79/79, YS and L1210 respectively. Cell-free extracts of YS and L1210 were tested for their ability to destroy $O^6$-meG when incubated with methylated DNA *in vitro*. The activity from YS was at least twice that from L1210.

These results indicate that cell survival is not always related to the level of DNA alkylation, and induced mutation frequency does not correlate with the rate of $O^6$-meG removal in these cell lines.

### PRETREATMENT OF RATS WITH A SINGLE DOSE OF ACETYLAMINOFLUORENE INCREASES THE CAPACITY OF LIVER ENZYMES TO MOVE $O^6$-METHYLGUANINE FROM DNA *IN VIVO* AND *IN VITRO*.

D. P. Cooper, G. P. Margison & P. J. O'Connor, *Paterson Laboratories, Christie Hospital and Holt Radium Institute, Manchester*

Chronic pretreatment of rats with AAF has been shown to enhance the repair of $O^6$-methylguanine ($O^6$-meG) (Buckley *et al.*, 1979; Nature 281) and similar effects have now been obtained using single-dose pretreatments. Male Wistar rats were given AAF up to 20 mg/kg; i.p. 24 h before a single dose (1 mg/kg, i.p.) of [$^{14}$C]-dimethylnitrosamine (DMN). 5 h after the DMN challenge there were some variations in the amounts of 7-methylguanine (7-meG) and 3-methly-adenine (3-meA) in the liver DNA of control and pretreated groups. However, the 3-meA/7-meG ratio was similar in all cases, whilst the $O^6$-/7-meG ratio was reduced after 20 mg AAF/kg, suggesting that repair of $O^6$-meG was enhanced. The effect was not specific for DMN-induced damage, as the repair of $O^6$-meG produced in liver by MNU was also enhanced. Incubation of cell-free liver extracts with MNU-methylated DNA *in vitro* decreased the amount of $O^6$-meG in the acid precipitable DNA. This decrease was protein-dependent, but no free $O^6$-meG base was found in the acid supernatants. Extracts from control rats destroyed 21% of the $O^6$-meG in the substrate, whilst extracts from animals pretreated with 6·67, 20 or 60 mg AAF/kg destroyed 40, 77 and 90% of the $O^6$-meG respectively. The effects observed *in vivo* were therefore probably due to an increased production of repair enzyme. AAF pretreatment did not affect 7-meG glycosylase activity in cell-free extracts. There was a 20-fold increase in the incorporation of [$^3$H]-dT into hepatic DNA of rats pretreated with 20 mg AAF/kg, whilst lower doses had no effect. The enhancement of $O^6$-meG repair could therefore be related either to cell proliferation as a result of the hepatotoxicity of AAF or to the induction of repair enzymes in response to sublethal DNA damage.

### EFFECT OF CHRONIC DIALKYNITROSAMINE ADMINISTRATION ON ALKYLGUANINE REMOVAL FROM SYRIAN GOLDEN HAMSTER LIVER DNA.

R. A. Smith & G. P. Margison, *Paterson Laboratories, Christie Hospital and Holt Radium Institute, Manchester*

Chronic administration of low doses of dimethylnitrosamine (DMN) or diethylnitrosamine (DEN) specifically enhances the enzymic removal of the promutagenic base, $O^6$-alkylguanine from rat liver DNA (Montesano *et al.*, 1979, *Cancer Res.*, **39**; Margison *et al.*, 1979, *Br. J. Cancer*, **40**). Investigations in Syrian golden hamsters indicate that the efficiency of $O^6$-alkylguanine removal from liver DNA is decreased after similar dose schedules. Once-weekly administration of unlabelled DMN (3 mg/kg s.c.) led to an increase in the amount of $O^6$-methylguanine (meG) produced by a single s.c. dose of 3 mg/kg [$^{14}$C]-DMN in comparison with controls. The kinetics of meG removal after administration of DMN (2 mg/kg/day i.p. on weekdays for 3 weeks) showed the increased amounts of $O^6$-meG in DNA were due to an inhibition or overloading of the enzyme involved. Daily DMN administration considerably increased the incorporation of 1-carbon fragments from [$^{14}$C]-DMN into DNA purines indicating that the schedule had increased DNA turnover without enhancing $O^6$-meG removal. Administration of DEN (10 mg/kg/day i.p. on weekdays for 3 weeks) also produced increased amounts of $O^6$-ethylguanine in DNA compared to controls given a single dose of 10 mg/kg$^-$[$^{14}$C]-DEN, indicating that the $O^6$-meG removal system acts on or is similarly affected by pretreatment with ethylating agents. The overloading of $O^6$-alkylguanine removal during chronic administration of dialkylnitrosamines may be a significant factor in the induction of liver tumours in Syrian hamsters.

### EFFECT OF AFLATOXIN $B_1$ ON THE REPAIR OF $O^6$-METHYLGUANINE IN THE HEPATIC DNA OF RATS AND MICE.

G. B. Maru, G. P. Margison, Y-H. Chu & P. J. O'Connor, *Paterson Laboratories, Christie Hospital and Holt Radium Institute, Manchester*

Pretreatment of male Wistar rats by i.p. injection of single (2 mg/kg) or multiple (4 × 0·5 mg/kg) doses of aflatoxin $B_1$ (AFB) stimulates the process for the removal of $O^6$-methylguanine from hepatic DNA in animals challenged with a single dose (2 mg/kg) of [$^{14}$C]-dimethylnitrosamine (DMN). The sensitivity of this system, in terms of its ability to "adapt", is indicated by the fact that the repair response can be detected as early as one day after pretreatment with a single dose (Chu *et al.*, 1981, *Br. J. Cancer*, **43**, 850). However, treatment of mice (C57BL, males) with doses from 0·1 to 100 mg/kg of AFB, had no marked effect on the activity of the $O^6$-meG repair system, when assayed *in vitro* using cell-free liver extracts and DNA methylated with [$^3$H]-MNU as substrate. This murine repair system has been titrated *in vivo*, using a range of doses of [$^{14}$C]-DMN (0·25–10 mg/kg). In terms of saturation kinetics it appears to be intermediate between the Syrian golden hamster (Stumpf *et al.*, 1979, *Cancer Res.*, **39**, 50), and the rat (Pegg & Hui, 1978, *Biochem. J.*, **173**, 739), but more closely resembling that for the rat. Different doses of AFB and exposure periods are under investigation, but it is tentatively concluded that the mouse, like the hamster and in contrast to the rat, is refractory to the inducibility of the $O^6$-meG repair system.

### DISTRIBUTION OF BENZO(A)-PYRENE-DNA ADDUCTS WITHIN MAMMALIAN CHROMATIN.

P. L. JACK & P. BROOKES, *Chemical Carcinogenesis Division, Institute of Cancer Research, Pollards Wood Research Station, Chalfont St. Giles, Bucks.*

The distribution of benzo(a)pyrene (BP)-DNA adducts within mammalian cell chromatin was examined by micrococcal nuclease digestion of nuclei from carcinogen-treated cells. Friend erythroleukaemia cells, prelabelled with [$^{14}$C]-dT, were treated with $^3$H-benzo(a)pyrene-7,8 diol-9,10-epoxide (BPDE) and a nuclear fraction prepared. Micrococcal-nuclease digestion of nuclei, harvested immediately after treatment, indicated a 4-fold enrichment of BP-DNA adducts on the linker region between nucleosome cores. Analysis of DNA, isolated from briefly digested nuclei by agarose gel electrophoresis, indicated that mononucleosomes released early during digestion contained slightly more adducts than total DNA. Treatment of primary mouse embryo cells with BPDE showed a similar enrichment of BP-DNA adducts within the linker region, however treatment of such cells with BP for 72 h (necessary to allow metabolic activation) showed no such preferential location. This apparent difference between the 2 agents was resolved by showing that post-treatment incubation of BPDE-treated cells led to a loss of the preferential linker binding. The time-dependent loss of preferential binding was shown to be independent of DNA replication, since it also occurred when hydroxyurea, an inhibitor of DNA synthesis, was present during post-treatment incubation of the confluent cultures.

### MEASURING AFLATOXIN ACTIVATION IN LIVER SLICES AND BY BACTERIAL MUTAGENESIS, AS AN INDICATOR OF SPECIES SUSCEPTIBILITY.

P. J. HERTZOG, S. C. BOOTH & R. C. GARNER, *Cancer Research Unit, University of York*

There is considerable species variation in susceptibility to aflatoxin $B_1$ (AFB) carcinogenesis, which is reflected in the extent of carcinogen interaction with liver DNA as measured in *in vivo* studies (*i.e.* rat > hamster > mouse). We have tested S-9 fractions of uninduced liver from these species for AFB activation in bacterial-mutagenesis assays, which are based on carcinogen-DNA interaction. Using *S. typhimurium* strains TA98 and TA100, hamster liver fractions produced the highest number of mutants/plate, followed by the rat and then the mouse.

When liver slices from these animals were incubated *in vitro* with [$^3$H]-AFB in an $O_2$ atmosphere, the pattern of species susceptibility was reflected in the level of binding of [$^3$H]-AFB to purified DNA (rat > hamster > mouse :: 30 : 11 : 1). Preliminary data using human liver slices indicates that DNA binding of AFB is considerably less than in rats.

The limitations of using bacterial-mutagenesis assays in this study could be due to the absence of cofactors for detoxification enzymes. Liver slices seem a reliable estimate of the *in vivo* situation, at least with regard to the level of DNA-bound AFB. One should be careful, therefore, when interpreting data from bacterial-mutagenesis screening; whereas these latter systems provide a good indication of the mutagenic/carcinogenic potential of a compound, they do not provide a good indication of potency or species susceptibility.

### INVESTIGATION OF THE BIOLOGICAL EFFECTS OF TUMOUR PROMOTERS: INHIBITION OF METABOLIC CO-OPERATION BY TPA.

A. R. KINSELLA, *Paterson Laboratories, Christie Hospital and Holt Radium Institute, Manchester*

It can be hypothesized that the phenomenon of tumour promotion involves aberrant mitotic segregation, which permits the expression of a specific recessive genetic or epigenetic chromosomal change present in initiated cells (Kinsella & Radman, 1976, *Proc. Natl Acad. Sci. U.S.A.* **75,** 6149). In favour of such a hypothesis we have demonstrated that the anti-promoting agent antipain inhibits MNNG-induced chromosome aberrations in V79 cells, in the absence of any effect on MNNG-induced mutagenesis. Conversely, TPA enhances MNNG-induced chromosome aberrations, in the absence of an effect on MNNG-induced mutagenesis to $6TG^{R}$. There is, however, considerable controversy surrounding the influence of TPA on carcinogen-induced forward mutagenesis. A comparison of *in situ* and replating mutagenesis assay techniques in this laboratory, has demonstrated that previous reports of TPA enhancement of forward mutagenesis were the result of a known artefact of the *in situ* assay. Reconstruction experiments show that the recovery of mutant colonies is markedly reduced above a certain critical cell density as a result of specific interclone metabolic co-operation. Thus, mutant expression is limited to the period between treatment and attainment of the critical cell density. TPA, by eliminating metabolic co-operation, enhances mutant recovery in such systems in the absence of any effect of mutagenesis *per se* (Kinsella, *Carcinogenesis*, **2,** 43). Comparison of the biological effects of the promoters, anthralin, oleic acid, iodoacetic acid and stilboestrol dipropionate with those of TPA, seems to confirm that promoting agents have no influence on mutagenesis *per se*. However, all these agents satisfy the requirements of the hypothesis, in inducing either numerical chromosomal changes or rearrangements.

**DETECTION OF CARCINOGEN-DNA ADDUCTS BY RADIO-IMMUNOASSAY.** R. SAFFHILL & J. M. BOYLE, *Paterson Laboratories, Christie Hospital and Holt Radium Institute, Manchester*

Certain butylating agents (*e.g.* butylnitrosourea and dibutylnitrosamine) are potent carcinogens, but their reaction with DNA has been little studied due to the limited specific activity of the radioactive carcinogen available, the low level of reaction with cellular DNA and the small amounts of DNA extractable from some tissues of interest, or from cultured cells. Some of these restrictions have, sometimes, limited the study of the reaction of methylating agents with DNA. We have now developed sensitive radio-immunoassay methods to detect specific butyl- and methyl-adducts formed in DNA, in particular those products that are potential promutagenic lesions (*viz.* $O^6$-alkyl-guanine, $O^2$- and $O^4$-alkylthymine). Polyclonal antibodies have been produced by immunizing rabbits or mice with the appropriate nucleoside–protein conjugate. Mouse hybrid cell lines (hybridomas) that produce monoclonal antibodies have been made. These yield highly specific antibodies, may be grown either *in culture* or as ascites. cells *in vivo* and may be stored frozen to provide a future source of standard antibody. We now have a variety of antibodies that are specific for $O^6$-butyldeoxyguanosine, $O^2$-butylthymidine, $O^4$-butylthymidine, $O^6$-methyldeoxyguanosine, $O^2$-methylthymidine, and $O^4$-methylthymidine. Using a Farr precipitation-inhibition assay we can detect sub-pmd ($<0{\cdot}1$ pmol) amounts of modified nucleoside in the presence of $\mu$mol amounts of parent (unmodified) nucleoside. With these radio-immunoassay methods it should be possible to quantitate the levels of several alkylation reaction products formed in cellular DNA both *in vivo* and *in vitro*.

**COLONIC LYMPHOID TISSUE AND ITS INFLUENCE ON TUMOUR INDUCTION IN DIMETHYLHYDRAZINE-TREATED RATS.** P. W. BLAND & D. C. BRITTON, *Department of Clinical Investigation, Royal United Hospital, Bath, and Pharmacology Group, University of Bath, Bath*

The mucosa of the large intestine contains a large amount of organized lymphoid tissue, but little is known of its contribution to antigen-processing, secretory immunity or tumour immunity. In the rat, mucosal lymphoid follicles are grouped together into discrete colonic lymphoid patches (CLP) in the proximal, mid and terminal colon. We have investigated the relationship between CLP and colonic neoplasia in the rat model of 1,2-dimethyl-hydrazine (DMH)-induced colonic adenomas and adenocarcinomas.

100 male, 7-week old Sprague-Dawley rats were injected s.c. with DMH (21 mg/kg) weekly for 20 weeks and groups were autopsied from weeks 20–40. 52 rats bore a total of 71 colonic tumours. 57 (80%) of these tumours (adenomatous polyps and both polypoid and sessile adenocarcinomas) arose from the epithelium overlying CLP, and many of the remainder were closely associated with isolated follicles.

Light and electron microscopy of CLP in control rats showed that constituent follicles were separated from the gut lumen by a flattened lymphoepithelium, highly specialized to facilitate antigen transport. Adjacent follicles were separated by groups of crypts generating normal columnar epithelium. The contribution to tumour induction in these interfollicular crypts from altered epithelial-cell kinetics and interaction with the underlying lymphoid elements, is currently under investigation.

**ANASTOMOSIS IS A FAVOURED SITE FOR TUMOUR DEVELOPMENT AFTER COLONIC SURGERY IN RATS.** J. B. BRISTOL, P. W. DAVIES, M. WELLS & R. C. N. WILLIAMSON, *University Department of Surgery, Bristol Royal Infirmary, Bristol*

Metachronous colorectal cancer may develop after partial intestinal resection because the remaining mucosa is hyperplastic (Williamson 1979, *R. Coll. Surg. Eng.*, **61**, 341). This possibility was tested in male Sprague-Dawley rats (n = 200) weighing $159 \pm 14$ g (s.d.). Five groups were subjected to either mid-colonic transection, right or left hemicolectomy, caecal resection or no operation (controls). Operation was performed either before or after 5-weekly s.c. injections of azoxymethane (10 mg/kg/wk) or vehicle.

Final tumour yields were unaffected by timing of surgery. There was no evidence of adaptive growth in the colon after partial colectomy, though in the ileum wet weight was increased by 22–40% after caecal resection or right hemicolectomy ($P < 0{\cdot}005$). Of the total of 217 large-bowel tumours, 23·5% occurred at the colonic anastomosis, 40% in distal colon, 20% in the rectum and 16·5% in proximal colon. Left hemicolectomy reduced the tumour yield by 52–88%, compared with transection or controls ($P = 0{\cdot}05$-$0{\cdot}005$). Caecal resection had no effect. Although right hemicolectomy produced a doubling in distal tumours ($P < 0{\cdot}02$) so did transection alone, and in each group this is largely attributable to the many anastomotic tumours. The anastomosis was also the site for the development of an invasive mucinous adenocarcinoma at the colorectal anastomosis, following left hemicolectomy in one rat receiving vehicle alone.

Ileocolic and colorectal anastomoses are favoured sites for tumour development after partial colectomy. Unaltered carcinogenesis in the remaining colon may reflect the lack of adaptive change.

**A COMPARISON OF RACIAL DISTRIBUTION OF CANCERS OF THE CERVIX AND PENIS AMONG 71 POPULATIONS IN 28 COUNTRIES.** I. K. CROMBIE & T. M. SORAHAN. *Cancer Epidemiology Research Unit, Department of Social Medicine, University of Birmingham*

Populations predominantly of European descent ("whites") experienced cervical-cancer incidence rates which clustered tightly around a median value $14{\cdot}0 \times 10^{-5}$. In contrast non-white incidences were more evenly spread about a higher median (22·4) and included many higher rates. A similar pattern, at lower incidences, emerged with penile cancer: whites clustered around the median (0·7); non-whites exhibited a wider range, a higher median (1·05) and no clustering.

A highly significant correlation ($P < 0{\cdot}001$) was found between the incidences of cervical and penile cancer among all registries. Of all the positive correlations between penis and the 45 female sites and cervix with the 43 male sites the largest co-efficient was obtained between penis and cervix; and no significant correlation was observed with any other genital site. Further examination revealed that the penis–cervix relationship was almost entirely due to the contribution of the "non-white" populations, among whom a large and significant ($P < 0{\cdot}002$) correlation co-efficient was observed; the positive coefficient among whites was small and non-significant. The category "non-white" is a heterogeneous collection of races. Subdivision into more homogeneous groupings revealed a clustering of incidences: thus the Japanese in particular, but other Asian groups as well, exhibited low incidences of both cancers; whereas South American populations, and those of African descent, experienced a high incidence of both. The marked correspondence between the incidence of these cancers indicates related aetiologies; possibly reflecting sexual mores or hygiene practices.

**SOME EPIDEMIOLOGICAL ASPECTS OF TESTICULAR CANCER IN ENGLAND AND WALES.** J. M. DAVIES, *Division of Epidemiology, Institute of Cancer Research, Sutton, Surrey*

In 1901 testicular cancer was a rare disease in this country, and death rates showed a typical peak in old age. Since then incidence and mortality rates among young men have risen dramatically and now show a marked peak around age 30; rates are still rising. Successive birth cohorts of men born from 1871 to 1951 had steadily increasing death rates up to age 45, and today testicular cancer is the commonest neoplasm registered among men aged 20–34. The disease is becoming more common in Western countries generally (especially in Denmark), but little is known about its aetiology or the reasons for the increasing incidence.

In 1971 mortality was highest among professional, administrative and clerical workers and lowest among manual workers, but differential social-class rates were already apparent in 1921 and 1931. U.S.A. data also show the disease to be most common among professional men. National mortality data covering 1957–61 suggest that testicular cancer is more common among single than married men, but evidence from the U.S.A. on this aspect is conflicting, and further research is needed.

**EPIDEMIOLOGY OF INDUSTRIAL BLADDER CANCER IN WEST YORKSHIRE.** R. A. CARTWRIGHT, *Yorkshire Regional Cancer Organization, Cookridge Hospital, Leeds;* R. W. GLASHAN, *Urology Department, Huddersfield Royal Infirmary*

This paper presents some preliminary results from a case-control study currently underway in parts of West Yorkshire. To date 1015 cases and 1288 controls have been analysed. Of these 72 cases and 37 controls were employed for at least 6 months in the chemical industry, giving a maximum-likelihood estimation of the risk ratio as 3·1 (95% confidence limits of 1·9–3·8). Other trades whose work brought them into contact with the chemical industry have an excess of bladder cancer cases amongst them, at a ratio of 2·5. Process workers associated with the dye-manufacturing industry, who form a substantial proportion of chemical workers, have a higher risk still of bladder cancer (4·5).

The case–control interviewing approach of this study makes it possible to estimate how other determinants of bladder cancer influence the risks for such hazardous occupations. Process workers who smoke have a risk ratio of nearly 6 whilst non-smoking process workers have a lower and statistically insignificant risk.

**A COMPARATIVE STUDY OF THE ANTITUMOUR ACTIVITY OF N-METHYLFORMAMIDE AND RELATED COMPOUNDS.** S. P. LANGDON*, N. W. GIBSON*, J. A. HICKMAN*, A. GESCHER*, M. F. G. STEVENS* & G. ATASSI†, **C.R.C. Experimental Chemotherapy Group, University of Aston, Birmingham; †Institut Jules Bordet,* 1000 *Brussels, Belgium*

N-Methylformamide (NMF, HCONHMe) was first reported to be active against the Sarcoma 180 tumour in 1953 (Clarke *et al.*, *Proc. Soc. Exp. Biol. Med.*, **84,** 203) and more recently has been found to be active against several human-tumour xenografts in mice. Renewed interest in NMF, and our interest in other antitumour agents with N-methyl groups which are important for biological activity has led us to investigate the activity of NMF, and some analogues, against tumours which are sensitive to the N-methyl group, requiring drugs such as hexamethylmelamine (HMM), DTIC and procarbazine. The M5076 ovarian carcinoma is one of the few murine tumours which is sensitive *in vivo* to HMM, and is also claimed to be an excellent model for the prediction of chemosensitivity in man (Simpson-Herren *et al.*, 1979, *Proc. Am. Assoc. Cancer Res.*, **80**) $10^6$ M5076 cells were implanted i.m. in the hind legs of 20g female $BDF_1$ mice and drugs administered i.p. daily as a single dose from Day 1 to Day 17. Tumour-volume inhibition on Day 24 was 100% for NMF, 64% for formamide (F) and 24% for N-ethylformamide (NEF) all at 300 mg/kg. Similar structure-activity relationships had been reported by Clarke *et al.* using the Sarcoma 180 and were confirmed by us, using this tumour. TLX5 lymphomas ($10^5$ cells s.c., drugs i.p. as a single dose on days 3–7) which were either sensitive or resistant to DTIC and procarbazine, both gave ~85% increase in survival time with 400 mg/kg NMF, but F and NEF had no significant activity at this dose, again confirming the requirement for the N-methyl group for significant *in vivo* antitumour effect.

**METABOLIC STUDIES ON THE ANTI-TUMOUR AGENT N-METHYLFORMAMIDE.** D. Ross, A. Gescher & J. A. Hickman, *CRC Experimental Chemotherapy Research Group, Department of Pharmacy, University of Aston, Birmingham*

Of all the formamides tested by others and by us against various murine tumours, N-methylformamide (NMF) is by far the most active. To test the hypothesis that the marked difference in activity is caused by differences in the metabolism of these agents, we investigated the biotransformation of NMF and N-ethylformamide (NEF) in male CBA CA mice. Parent compounds and their metabolites were identified by gas chromatography using an $N_2$-sensitive detector. Formamide (F) was the major urinary metabolite of both agents. When NMF and NEF were incubated with mouse liver preparations (whole homogenate, 9000*g* supernatant, microsomes) at varying substrate concentrations and in the presence and absence of $O_2$ significant metabolism could not be detected.

We also investigated the influence of a series of N-alkylformamides on tissue levels of the endogenous non-protein thiolglutathione (GSH). GSH has been suggested to regulate the toxicity of drugs like Adriamycin (Olson *et al.*, 1980, *J. Pharmacol. Exp. Ther.*, **215**, 450) and paracetamol. 400 mg/kg NMF given i.p. to mice lead to a marked decrease of GSH in the liver; by 59·8% 1 h after drug administration, whereas equimolar doses of other N-alkylformamides had no depleting effect. The hepatic depletion of GSH by NMF may be related to the hepatotoxicity of NMF seen in an early clinical trial (Laird Myers *et al.*, 1956, *Cancer*, **9**, 949).

**EFFECT OF ALKYLATING AGENTS AND OTHER DRUGS ON THE UPTAKE OF MELPHALAN (M) BY MURINE L1210 LEUKAEMIA CELLS *IN VITRO*.** A. D. Martin, R. W. G. Beer, A. G. Bosanquet & E. D. Gilby. *Department of Medical Oncology, Royal United Hospital, Bath*

M is a standard drug in the treatment of multiple myeloma. In recent years combination therapy with prednisolone has improved the response rate and survival times of patients, but addition of further cytotoxic drugs to treatment regimens has produced little significant improvement. With an experimental technique adapted from Vistica *et al.*, 1978 (*Mol. Pharmacol.*, **14**, 1136) we have investigated the effect of these and other drugs on M transport *in vitro*, as it is possible that changes in response to treatment may be related to changes in the transport of M. We have confirmed that M uptake is an active process competitively inhibited by L-leucine. In 36 experiments in amino-acid-free medium the mean concentration of M taken up was 225 pmol/$10^6$ cells. High-pressure liquid chromatographic analysis of the cell sap showed that most of the drug is present as free native M. The nitrosourea BCNU was the only drug of 33 tested (at 1 and 10 times their approximate serum concentrations in man) which stimulated the uptake of M, and did so by 76% after 30 min. However, methyl CCNU, with a similar structure to BCNU, depressed M uptake by 40%. Incubation with adriamycin, aminophylline, chlorpromazine, activated cyclophosphamide, mustine, ouabain or vincristine produced a decrease of 20–35% in uptake of M. Transport was reduced to a lesser extent by incubation with chlorambucil, frusemide or indomethacin. It is possible that the inhibition of M transport by these drugs when used in combination with M could reduce the effectiveness of a multi-drug regimen.

**EFFECT OF L-METHIONINE DEPRIVATION ON THE PROLIFERATIVE ACTIVITY OF NORMAL AND LEUKAEMIC MARROW CELLS *IN VITRO*,** S, Eridani, B. Sawyer, S. Villa & M. Tisdale, *Departments of Haematology and Biochemistry, St Thomas's Hospital and Medical School, London*

Following previous work (Tisdale, 1980, *Cell. Biol. Int. Rep.*, **4**, 563) suggesting that some tumour cells may have a high requirement for methionine, which in addition to protein synthesis is used for nucleic-acid methylation and polyamine biosynthesis, a comparison has been made between normal and leukaemic marrow (obtained from patients with ALL, AML and CML) with regard to the capacity to incorporate methyl-$^3$HdT into acid-insoluble material, after incubation in media lacking L-methionine and supplemented with L-homocysteine. A lower incorporation was shown by leukaemic cells, than normal ones, with a significant difference ($P < 0{\cdot}05$). Marrow cells of leukaemic patients in remission behave as normal cells.

This difference is not due to inability of leukaemic cells to synthesize L-methionine from homocysteine, because an increased incorporation can be seen in the presence of homocysteine when methionine concentration in culture becomes limiting. Further experiments suggest that such high methionine requirement might be due to a decreased transport capacity by leukaemic cells: all marrows show the same linear uptake for the whole range of concentrations tested, but the maximum uptake velocity is lower in leukaemic cells. Restriction by different means of the supply of methionine to leukaemic cells might play a role in the control of myeloproliferative disorders.

### QUANTITATION OF CARCINOGEN-INDUCED RESISTANCE IN RAT HEPATOCYTES TO CYTOTOXICITY BY ADRIAMYCIN *IN VITRO*. B. I. Carr, *City of Hope National Medical Center, Duarte, California, U.S.A.*

The hepatocytes from rats which were treated with a variety of hepatocarcinogens *in vivo* have been shown to develop resistance to Adriamycin-induced cytotoxicity, as measured by an *in vitro* assay using trypan-blue exclusion (Carr, *BACR*, 1980). In order to determine the quantitative relationships between the amount of carcinogen and the subsequent development of resistance, a single dose of 2-acetylaminofluorene (AAF) was injected into male Fischer F344 rats weighing 200–220 g, and 18 h later their hepatocytes were harvested and placed in primary monolayer culture for a subsequent 24 h. Cell viability was then assessed in plates which contained Adriamycin and compared to that in controls. It was found that a single injection of 5 mg/kg AAF could induce resistance to Adriamycin ($1{\cdot}8 \times 10^{-4}$M) when measured after 24 h in culture. Less than 2·5 mg/kg or more than 25 mg/kg of AAF failed to induce comparable resistance. When the rats were harvested sequentially after a single dose of 5 mg/kg AAF it was found that the carcinogen-induced resistance to Adriamycin cytotoxicity was no longer present 7 days after injection. By contrast, 0·02% AAF given continuously in the diet led to a stable resistance that did not decrease with time.

Resistance to Adriamycin which is induced by a single dose of carcinogen now permits the design of experiments to examine the quantitative and temporal relationships in rat liver, between induction of resistance to cytotoxicity and altered cell growth.

### A MODEL RELATING DRUG TRANSPORT, PROLIFERATION-DEPENDENCE OF CYTOTOXICITY AND THE EMERGENCE OF RESISTANCE TO THE CURRENTLY USEFUL ALKYLATING AGENTS (AA). J. E. Byfield & P. M. Calabro-Jones, *University of California, San Diego, California, U.S.A.*

Using human T-lymphocyte clonogenesis *in vitro* we have studied the effect of the induction of proliferation (by PHA) on the sensitivity of T-cells to AA killing. It was found that resting cells are considerably more resistant ($> D_0$ and $\geqq D_0$) to some agents (melphalan (MEL), *cis*-platinum, $HN_2$) than cycling cells. No effect of the cell proliferative state could be found for BCNU, Me-CCNU, Mitomycin C, Procarbazine, ACNU and X-rays. The toxicity of MEL was reduced by competition with normal amino acids, confirming the work of others. This confirms that MEL is probably taken up by amino-acid transport carriers in normal human cells. Glucose-containing drugs (*e.g.* chlorozotocin) and activated cyclophosphamide (phosphoramide mustard) were ineffective against these mature T cells, though cytotoxic against epithelial cells in culture. The data is consistent with a model in which the clinically useful AA fall into two distinct groups: (1) those drugs that are water-soluble, effectively lipid insoluble and whose entry into cells is (membrane) carrier-dependent; (2) those drugs that are lipid soluble or amphipathic (carrier-independent, CID) and that enter cells by simple diffusion. CID agents all show prolonged marrow depression and generally reduced therapeutic ratios. We believe this relates to a greatly reduced dependence on proliferation, since these must also directly enter normal resting (marrow and gut) stem cells. The model permits a rationalization of several aspects of AA anti-cancer activity including (new) drug design.

**ENHANCEMENT OF ANTITUMOUR ACTIVITY BY $H_2$-RECEPTOR ANTAGONISTS.** M. COLLINS, *Cancer Chemotherapy Department, Imperial Cancer Research Fund, London.*

The antitumour effect of razoxane (RZ) is enhanced by pretreatment with the $H_2$-receptor antagonist cimetidine (Collins, 1980, *Br. J. Cancer*, **42,** 173). The specificity of this enhancement on the Walker tumour was investigated.

In rats pretreated with cimetidine, metiamide or ranitidine, the antitumour effect seen in response to RZ was significantly greater than in rats treated only with RZ. A similar degree of enhancement was achieved using equiactive doses of the 3 antagonists against gastric acid secretion in the rat. None of the $H_2$-receptor antagonists showed significant antitumour activity. Pretreatment with an inactive analogue of cimetidine gave no enhancement of RZ antitumour activity.

Rats were pretreated with cimetidine before administration of RZ, cyclophosphamide, methotrexate or 5-fluorouracil; enhancement of antitumour activity was only seen with the combination of cimetidine and RZ.

Some structural requirement or activity shared by cimetidine, metiamide and ranitidine, and not by the inactive analogue, appears to be necessary for enhancement of RZ antitumour activity. The reason for the specificity of this enhancement to RZ is being investigated.

**IMPROVING THE THERAPEUTIC INDEX OF CYCLOPHOSPHAMIDE (CY) IN IMMUNE-DEPRIVED ANIMALS BEARING HUMAN OAT-CELL LUNG TUMOUR XENOGRAFTS.** B. D. EVANS, I. E. SMITH & J. L. MILLAR, *Institute of Cancer Research and Royal Marsden Hospital, Sutton, Surrey.*

It was established in 1978 that the lethal effects of high-dose cyclophosphamide (400 mg/kg) could be offset by pretreatment with low-dose CY (50 mg/kg) given 4 days before (Millar & McElwain, 1978, *Antibiot. Chemother.*, **23,** 271). In contrast, experimental murine tumours do not appear to be protected by similar CY pretreatment (Millar *et al.*, 1980, *Br. J. Cancer*, **42,** 485).

Human oat–cell lung tumour xenografts grown in immune-suppressed mice have been shown to have chemotherapeutic responses which correlate closely with those in the patient from whom the original tumours were obtained (Shorthouse *et al.*, 1980, *Br. J. Surg.*, **67,** 715).

First, we demonstrated that it is possible to protect immune-deprived mice similarly to normal mice with CY pretreatment. Then we used the xenograft system to test whether CY pretreatment could be used to increase the therapeutic index of CY by decreasing normal tissue toxicity whilst maintaining the anti-tumour effect.

Two groups of 8 CBA/lac immune-suppressed mice with bilateral flank implantations of a human oat-cell xenograft were treated with 300 mg/kg CY i.p., with or without pretreatment with CY (50 mg/kg) 4 days earlier. At 8 weeks, 6 of the pretreated animals remained alive (75%) compared with none of the controls (0%). In the pretreated group 10/13 tumours were in complete remission (77%) compared with 7 out of 12 in the controls (58%). These results show that CY treatment enhances the therapeutic ratio of large-dose CY in this oat-cell xenograft system, and this may have useful clinical implications.

**ENHANCEMENT OF THE RADIATION RESPONSE OF HYPOXIC MAMMALIAN CELLS *IN VITRO* BY A PLATINUM CO-ORDINATION COMPLEX.** A. H. W. NIAS & M. LAVERICK, *Richard Dimbleby Department of Cancer Research, St Thomas's Hospital Medical School, London*

It is well established that certain platinum complexes of the *cis* configuration are potent antitumour agents in their own right. We now report the effect of the platinum complex—*cis* dichloro *bis* (isopropylamine) *trans* dihydroxy platinum IV (CHIP) given as a pretreatment to X-irradiated cultures of Chinese hamster ovary (CHO) cells and C3H mouse mammary tumour cells *in vitro*. CHIP was given as a 1 h pretreatment at 37°C with varying intervals before X-irradiation under aerated and hypoxic conditions. Schedules involving 1, 3, 5, and 24h intervals between drug and radiation in hypoxic CHO cells gave enhancement ratios (ER) of 1·7, 1·9, 1·36 and 1·0 respectively. The extrapolation numbers remained unaltered. A 1h delay in drug and radiation treatment of aerated CHO cells gave a much lower ER (1·1) but the extrapolation number was reduced. C3H mouse mammary-tumour cells showed no enhancement by CHIP of the response of aerated cells to X-ray damage. Enhancement was only found under hypoxia. A maximum ER of 1·7 was obtained after a 1–3h delay between drug treatment and radiation. By 5 h, ER was reduced to 1·14 and at 8 h there was no evident enhancement of cell killing. Under optimal conditions of drug and X-ray (1h delay) the effect of CHIP pretreatment on hypoxic cells is manifested not only as a decrease in $D_0$, which is measured by ER, but also as a reduction in the "shoulder region" to a point where survival is identical to that in aerated X-irradiated tumour cells. These results emphasize the importance of finding the optimal regimes of drug dose and timing of the subsequent X-irradiation.

**DNA CROSS-LINKING INDUCED BY PENTAMETHYLMONOMETHYLOL-MELAMINE *IN VITRO*.** J. R. F. MUINDI, C. J. RUTTY & K. R. HARRAP, *Department of Biochemical Pharmacology, Institute of Cancer Research, Sutton, Surrey*

Pentamethylmonomethylolmelamine (CB 10-369) the primary product of oxidative N-demethylation of hexamethylmelamine 7 (HMM) (*Life Sciences*, 1980, **26**, 147) is toxic to a number of tumour cell lines *in vitro* and has antitumour activity *in vivo*. CB 10-369 inhibits the incorporation of thymidine into DNA of L1210 and PC6 cells *in vitro*, though only the PC6 tumour is sensitive *in vivo*. Formaldehyde, which is one of the products of chemical breakdown of N-methylolmelamines, similarly inhibits dT incorporation in these two cell types. DNA cross-linking was assayed by the alkaline-elution technique of Kohn *et al.* (*Meth. Cell. Res.* 1979, **16**, 309). Both formaldehyde and CB 10-369 produced extensive total cross-links in L1210 cells. These cross-links were susceptible to treatment with proteinase K, and are therefore of the DNA–protein type. Formaldehyde, but not CB 10–369, also induced DNA strand breaks in L1210 cells. In contrast to melphalan, no DNA-DNA cross-links were observed up to 24 h after treatment with CB 10-369. Furthermore, the DNA–protein cross-linking induced by CB 10-369 and formaldehyde could be completely reversed by semicarbazide. Despite the *in vivo* sensitivity of the PC6 tumour, neither DNA-DNB nor DNA–protein cross-links could be demonstrated in these cells, using an equitoxic concentration of the N-methylolmelamine. Formaldehyde, however, did produce DNA–protein cross-links in PC6 cells, and these were again reversed by semicarbazide. Thus it would appear that the antitumour action of N-methylolmelamines is not attributable to DNA cross-linking.

**EXPERIMENTAL STUDIES ON TRIMETHYLTRIMETHYLOLMELAMINE AS AN ALTERNATIVE TO HEXAMETHYLMELAMINE (HMM) AND PENTAMETHYLMELAMINE (PMM).** D. R. NEWELL, C. J. RUTTY, J. R. F. MUINDI & K. R. HARRAP, *Department of Biochemical Pharmacology, Institute of Cancer Research, Sutton, Surrey*

HMM is an established anticancer drug which has shown activity against ovarian carcinoma, lung carcinoma and certain lymphomas. PMM, a water soluble alternative to HMM, has recently undergone a number of Phase I trials which, however, failed to demonstrate complete or partial responses in man (*Proc. Am. Ass. Cancer Res.*, (1980) **21**, 136, 143, 178, 347). It is postulated that the relatively slow metabolism of PMM to N-methylolmelamine in man fails to generate therapeutic levels of these highly cytotoxic species. In contrast, in the rat and mouse, more rapid metabolism allows cytotoxic levels of these metabolites to accumulate. The direct administration of an N-methylolmelamine would circumvent the requirement for metabolic activation.

For this reason we have studied $N^2$, $N^4$,$N^6$-trimethylol-$N^2$,$N^4$,$N^6$-trimethylmelamine(CB 10-375). This compound has similar antitumour activity to HMM and PMM against a number of experimental tumours, whilst CB 10-375 induces less neurotoxicity than PMM in rats and mice. Pharmacokinetic studies have demonstrated that CB 10-375 administration, to rats and mice, produces higher peak plasma concentrations of N-methylolmelamines (rat = 450 $\mu$M, mouse = 740 $\mu$M) than does PMM (rat = 225 $\mu$M, mouse = 575 $\mu$M). By analogy, the administration of CB 10-375 to man may similarly result in raised levels of N-methylolmelamines, which could then be sufficient for a therapeutic effect. In addition, a medium has been selected (10mM $NaHCO_3$) which stabilizes the N-methylol moieties sufficiently to facilitate clinical administration without exposing patients to formaldehyde.

**HYPERTHERMIA AND CYTOTOXIC DRUGS—COMBINED EFFECTS ON NORMAL MOUSE BONE MARROW CFU-S.** D. Honess & N. M. Bleehen, *MRC Clinical Oncology and Radiotherapeutics Unit, Hills Road, Cambridge*

A limiting toxicity of several cytotoxic agents whose tumoricidal effects are enhanced by hyperthermia to the marrow. The spleen-colony-forming unit (CFU-S) assay of Till & McCulloch, measuring survival of undifferentiated marrow stem cells, provides one method of quantitating marrow damage, and has been used in this work to assess the effects of combinations of hyperthermia with cytotoxic drugs on marrow function.

Modest whole-body hyperthermia was administered to unanaesthetized, unrestrained C3H/He mice by enclosing them in an incubator with a fresh air supply. This treatment rapidly induced a rectal temperature of 41°C ± 0·2°C which was maintained for 45 min, and this alone caused no change in survival of stem cells. Drugs were administered i.p. just before the start of heating. Unheated mice were left at room temperature.

Room-temperature dose response curves for cyclophosphamide and BCNU, giving a drop in survival of up to one decade were obtained, assays being carried out at 2 and 24 h after giving the drug. It was found that for cyclophosphamide at 200 mg/kg the hyperthermia caused a 10-fold increase in stem-cell killing at both times; for BCNU at 60 mg/kg there was a similar increase in killing at 24 h, but only 3-fold at 2 h.

These data indicate the need for directly comparable tumour-cell-killing data in order to estimate the therapeutic ratio of the combined treatments.

**FLOW CYTOFLUOROMETRIC ESTIMATION OF RELATIVE ADRIAMYCIN BINDING TO DNA AFTER HYPERTHERMIA *IN VITRO*.** S. H. Chambers & N. M. Bleehen, *MRC Unit of Clinical Oncology and Radiotherapeutics, Cambridge*

A method has been devised to estimate the relative amount of Adriamycin (ADM) bound to DNA in cells treated with pharmacologically relevant doses *in vitro*. ADM is an intercalating agent which interferes with the staining of DNA by the fluorescent dye ethidium bromide. Cells were treated with a range of ADM doses from 0 to 10 $\mu$g/ml. Staining was then performed with ethidium bromide at 2·5 $\mu$g/ml in 0·1% tri-sodium citrate. The resulting fluorescence distributions from isolated nuclei were measured with a flow cytometer. It was found that increasing ADM concentration reduced the intensity of the fluorescence emissions, and the results indicate that doses differing by 1 $\mu$g/ml can be resolved in this system, so long as strict control conditions are adhered to.

The method has been used to investigate the effect of heat on the binding of ADM in cells. The results show that there is greater binding of ADM to DNA after 1 h at 43°C than after 1 h at 37°C for drug doses from 1 to 30 $\mu$g/ml. Secondly, this increase may be due to an increased rate of binding of ADM, longer exposures at 37°C raise the ADM levels which tended to approach those of the 43°C cells. This method has potential for measuring low ADM levels in cells isolated from tumours, and benefits from only requiring small cell numbers.

**MODIFICATION BY MISONIDAZOLE AND METRONIDAZOLE OF THE RESPONSE OF THE RIF-1 MOUSE SARCOMA TO CYTOTOXIC DRUGS.** P. R. TWENTYMAN & P. WORKMAN, *MRC Clinical Oncology and Radiotherapeutics Unit, Cambridge*

Studies have been carried out into the ability of misonidazole (MISO) and metronidazole (METRO) to modify the response of the RIF-1 sarcoma to a range of cytotoxic drugs. Survival of clonogenic tumour cells 24 h after treatment has been used as the primary assay of tumour response, and growth delay has been measured as a secondary endpoint. The nitroimidazoles were administered by the i.p. route at a dose of 2·5 mmol/kg and at 30 min before the cytotoxic drugs.

Little change in the response to melphalan was brought about by MISO or METRO pretreatment. For cyclophosphamide a reduction in the shoulder of the cell-survival curve was seen with MISO pretreatment, the subsequent curves being parallel. This finding is in agreement with our previous growth delay studies.

A much larger increase in sensitivity was observed if MISO was given before CCNU or chlorambucil. The effect appeared to be dose-modifying by a factor of $\sim 2$. The enhancement of these two agents was much less for METRO pretreatment than for MISO.

Our current investigations include a study of changes in haemopoietic toxicity brought about by the addition of nitroimidazoles to cytotoxic drug treatment.

**STRUCTURE-ACTIVITY RELATIONSHIPS FOR THE ENHANCEMENT OF THE ANTI-TUMOUR EFFECT OF CCNU BY ELECTRON-AFFINIC AGENTS.** P. WORKMAN & P. R. TWENTYMAN, *MRC Clinical Oncology and Radiotherapeutics Unit, Cambridge*

The radiosensitizer misonidazole (MISO) has been shown to increase the *in vivo* effect of some cytotoxic drugs against some transplantable tumours, *e.g.* the nitrosourea CCNU against the KHT sarcoma in C3H mice (Sieman, *Br. J. Cancer* (in Press); Twentyman, *Br. J. Radiol.* (in Press). Using a regrowth-delay assay with the KHT tumour, we have compared the ability of a range of electron-affinic agents to enhance the anti-tumour activity of CCNU. No regrowth delay was seen with the electron-affinic agents alone. Agents were given (usually i.p.) 30 min before 10 mg/kg CCNU i.p. MISO (2·5 mmol/kg = 500 mg/kg) increased the regrowth delay from about 1·5 to 3·5 days (compared to about 8 days with 20 mg/kg CCNU alone). Using a fixed dose of 2·5 mmol/kg, we examined a series of 2-nitroimidazoles similar in electron affinity to MISO, but differing in octanol-water partition coefficient (P) over 3 orders of magnitude (0·016-20). Those more hydrophilic than MISO (P = 0·43), including desmethylmisonidazole, SR-2508 (i.v.) and SR-2555 (i.v.), were inactive, whereas those more lipophilic tended to be more active than MISO. Two fairly lipophilic 5-nitroimidazoles, nimorazole (P = 1·4) and metronidazole (P = 0·96), showed similar or greater activity, despite their considerably lower electron affinity. Two basic 2-nitroimidazoles, Ro 03-8799 and RSU 1047, similar in electron affinity to MISO, had about the same activity as MISO. We also tested, at maximum tolerated doses, a number of agents with electron affinity much greater than MISO. Nitrofurantoin (50 mg/kg), duraquinone (250 mg/kg) and menadione (32 mg/kg) had little or no activity; nitrofurazone (125 mg/kg) showed more activity, but less than MISO. The microsomal enzyme inhibitor SKF 525A (50 mg/kg) markedly increased the effect of CCNU. It is possible that the enhancement of CCNU activity by high doses of lipophilic analogues *in vivo* may be due in part to inhibition of CCNU metabolism.

**DISPOSITION KINETICS AND METABOLISM OF CB 1954 IN MICE AND DOGS.** R. A. S. WHITE & P. WORKMAN, *Department of Clinical Veterinary Medicine and MRC Clinical Oncology and Radiotherapeutics Unit, Cambridge*

CB 1954 (2,4-dinitro-5-azirdinylbenzamide) is highly selective against the Walker 256 rat carcinosarcoma, but shows little effect on other tumours (Khan & Ross, 1969, *Chem-Biol. Interact.*, **1**, 27; Cobb *et al.*, 1969, *Biochem. Pharmacol.*, **18**, 1519). It also has interesting radiosensitizing properties (Stratford *et al.*, 1981, in Press), but little is known of the pharmacokinetics. We developed a novel HPLC assay for the rapid analysis of CB 1954 and its principal metabolites, and used it to compare pharmacokinetics in mice and dogs. With an i.v. dose of 50 mg/kg in mice the kinetics in blood were biphasic, with a distribution-phase $t\frac{1}{2}$ of 4 min and elimination phase $t\frac{1}{2}$ of 1·4 h. Given i.p. (25-100 mg/kg) the elimination $t\frac{1}{2}$ was similar and the bioavailability (AUC i.p./AUCi.v.) was complete. In dogs the plasma kinetics at 10–25 mg/kg i.v. were monophasic, with a $t\frac{1}{2}$ of 3–4 h. After oral administration in gelatin capsules the elimination $t\frac{1}{2}$ was similar and the oral bioavailability (AUCoral/AUCi.v.) was 50%. In mice, urinary excretion of unchanged drug was 20%. In both species circulating concentrations greatly exceeded those required to inhibit growth of Walker tumour cells *in vitro*. Concentrations of CB 1954 in EMT6 mouse tumours were identical to those in blood from 2 h onwards. Brain levels were half those in tumour throughout. Repeated doses of CB 1954 (30 mg/kg/day i.p. × 5) had no effect on the drug's kinetics in mice; phenobarbitone (80 mg/kg/day i.p. × 5) decreased the $t\frac{1}{2}$ by 10%, and increased the rate of aziridine-ring removal, without affecting nitroreduction. The xanthine-oxidase inhibitor allopurinol (32 mg/kg i.p.) did not alter CB 1954 levels in mice, but appeared to delay the excretion of the nitroreduction product; the protective compound phenylAIC (100 mg/kg i.p.) had no effect.

**CORRELATION OF CYTOSINE ARABINOSIDE (ARA-C) PHARMACOKINETICS, ARA-C PHOSPHORYLATION, INTRACELLULAR ARA-CTP HALF-LIFE AND EFFECTS ON DNA SYNTHESIS IN ACUTE MYELOID LEUKAEMIA.** A. L. Harris & D. G. Grahame-Smith, *MRC Clinical Pharmacology Unit, Radcliffe Infirmary, Oxford*

Clinical resistance to Ara-C may be due to short plasma ½-life, poor Ara-C phosphorylation or reduced sensitivity of DNA synthesis to Ara-C in blasts *in vitro*. We have measured Ara-C plasma levels after 2 mg/kg bolus (Harris *et al.*, 1979, *Br. J. Clin. Pharmacol.*, **8,** 219) Ara-C phosphorylation to Ara-CTP and inhibition of DNA synthesis by Ara-CTP in intact blasts *in vitro* (Harris *et al.*, 1980, *Br. J. Haematol.*, **45,** 371). Ara-CTP intracellular ½-life was measured under identical conditions. In 5 patients all these measurements were performed before treatment with Ara-C and daunorubicin. The interaction of these variables could then be simulated using the patients' *in vivo* and *in vitro* data. Below 10 nM Ara-C there was no effect on DNA synthesis *in vitro*. The area under the plasma Ara-C concentration-time curve (AUC until Ara-C fell below 10 nM) was 22·8–138 μM/min. Ara-CTP production *in vitro* was 0·015–0·6 μmol/$10^{12}$ blasts/min/1 μM Ara-C. 95% of maximal simulated Ara-CTP production *in vivo* occurred by 45 min after a bolus. Ara-CTP intracellular ½-life was 30–120 min and 50% inhibition of DNA synthesis was produced by 0·13–0·65 μmol Ara-CTP/$10^{12}$ blasts. Simulated duration of >50% inhibition of DNA synthesis was 4–14 h. Simulations showed that increasing Ara-CTP intracellular ½-life from 30 to 60 min had a greater effect on duration of inhibition of DNA synthesis than a 5-fold increase in Ara-CTP levels or a 5-fold increase in sensitivity of DNA synthesis to Ara-C. In patients with low Ara-CTP levels 8-hourly i.v. boluses produced more inhibition than the same total dose by constant 24 h infusion. These results explain the poor predictive value of previously described variables, show the importance of intracellular Ara-CTP ½-life, and suggest ways of optimising Ara-C use in resistant patients.

**POLICY OF MINIMAL INTERVENTION IN THE MANAGEMENT OF LOW-GRADE NON-HODGKIN'S LYMPHOMA OF THE FOLLICLE-CENTRE CELL TYPE.** C. McCormick, R. C. F. Leonard, *Oxford Lymphoma Group, Churchill Hospital, Oxford*

In a prospective study of non-Hodgkin's lymphoma using the Kiel classification (Gerard-Marchant *et al.*, 1974, *Lancet*, ii, 406) a group of 60 patients with ML centroblastic-centrocytic was selected for conservative management. After biopsy and staging, 18 patients, including 12 with multiple-site (Stage II–IV) disease were given no treatment, a second group of 17 patients, including 9 with multiple-site disease, local radiotherapy for bulk disease, and a third group of 25 patients (all with multiple-site disease) chemotherapy.

In the "no treatment" group 6 patients eventually had radiotherapy and 5 chemotherapy; 3 of these 11 have died. The remainder are well between 22 and 52 months from diagnosis. Six deaths (one with single-site, 5 with multiple-site disease) occurred in the radiotherapy group and 11 are well 12–55 months from diagnosis. Ten deaths occurred in the chemotherapy group and 15 are well 14–58 months from diagnosis.

The actuarial survival projected for the whole group is 63%, which is not inferior to published reports of similar patients treated more aggressively with the intention of eradicating disease.

**PHARMACOKINETICS OF SUBCUTANEOUS CYTOSINE ARABINOSIDE IN PATIENTS WITH ACUTE MYELOBLASTIC LEUKAEMIA:** M. L. SLEVIN*, E. M. PIALL†, G. W. AHERNE†, A. JOHNSTON‡, M. C. SWEATMAN* & T. A. LISTER*. **Imperial Cancer Research Fund Dept of Medical Oncology, St Bartholomew's Hospital, London: †Division of Clinical Biochemistry, Dept of Biochemistry, University of Surrey: ‡Dept of Clinical Pharmacology, St Bartholomew's Hospital, London*

The pharmacokinetics of s.c. (Ara-C) were compared with bolus i.v. injection and i.v. infusion in 5 patients with AML.

Ara-C plasma levels were measured by the radioimmunoassay developed by Piall *et al.* (*Br. J. Cancer*, 1979, **40,** 548). S.c. Ara-C was rapidly absorbed with a half-life of absorption of $3{\cdot}36 \pm 0{\cdot}95$ min, and then declined biexponentially with a mean initial half-life of $15{\cdot}6 \pm 4{\cdot}8$ min, and a mean terminal half-life of $1{\cdot}6 \pm 0{\cdot}1$ h.

Following i.v. bolus administration Ara-C declined triexponentially, with the initial phase being divided into two exponentials. The mean half-life of the first phase was $1{\cdot}92 \pm 0{\cdot}28$ min, the intermediate phase $14{\cdot}1 \pm 1{\cdot}3$ min and the terminal phase $8{\cdot}5 \pm 3{\cdot}7$ h. (Erratic values were obtained from 2 patients during the terminal phase and these gave rise to the large mean terminal half-life).

Levels were above the steady-state infusion levels for only 40 min after the i.v. bolus and for 100 min after s.c. administration.

The decline in Ara-C was rapid after both routes of administration, and after 5 h levels were $\sim 10\%$ of the steady-state infusion levels.

The results of this study demonstrate that it is not possible to achieve comparable steady-state levels of Ara-C with the same total dose given by s.c. bolus and by continuous i.v. infusion.

**A PHASE I AND II STUDY OF m-AMSA IN ACUTE LEUKAEMIA.** M. L. SLEVIN*, M. S. SHANNON†, H. G. PRENTICE†, A. J. GOLDMAN* & T. A. LISETR*, **Imperial Cancer Research Fund Department of Medical Oncology, St Bartholomew's Hospital, London:* †Academic Department of Haematology, Royal Free Hospital, London

32 patients with relapsed or resistant acute leukaemia were treated with m-AMSA at doses ranging from 50–150 mg/m$^2$ daily for 5 days.

Complete remission was achieved in 3/18 patients with AML, 2/9 patients with ALL and 0/5 patients with CML in blast crisis. In addition, partial remission was noted in 6/18 patients with AML, 4/9 patients with ALL and 2/5 patients with CML blast crisis. The complete remissions all occurred at or above 100 mg/m$^2$/day.

Haematological toxicity occurred in all patients and was dose related. Nausea and vomiting occurred in 8/26 courses at 50 mg/m$^2$ and 12/18 courses at 150 mg/m$^2$, but were generally mild and easily controlled. Alopecia was uncommon at the lower doses, but occurred in all patients receiving the higher doses. Stomatitis was noted in only 2/26 courses at 50 mg/m$^2$ but in 8/16 courses at 150 mg/m$^2$. Mild and transient elevation of liver enzymes was common. No evidence of renal failure or neurotoxicity was seen.

m-AMSA is an active drug in acute leukaemia, with acceptable toxicity. Its place in combination chemotherapy is now being explored.

**AMINOGLUTETHIMIDE IN ADVANCED BREAST CANCER: EFFECTS OF DOSE ON HORMONE LEVELS AND RESPONSE.** A. L. HARRIS*, M. DOWSETT†, I. E. SMITH*, S. JEFFCOATE†, **Royal Marsden Hospital, Fulham Road, †Chelsea Hospital for Women, London*

Aminoglutethimide (AG), combined with hydrocortisone, is a useful drug in advanced postmenopausal breast cancer. It inhibits adrenal steroid synthesis and peripheral conversion of adrenal androgens to oestrone. The minimum effective dose is unknown, the usual dose being 1 g/day, and side effects increase with AG dose. We have therefore studied 28 consecutive postmenopausal patients with advanced breast cancer and measured oestrone levels before treatment, and after 500, 750 and 1000 mg daily, with 20 mg hydrocortisone twice daily. Dehydroepiandrosterone-sulphate (DHEAS), the main adrenal androgen, was also measured. Both hormones were measured by radio-immunoassay. The results are shown below:

| | Pretreatment | Daily AG dose (mg) 500 | 750 | 1000 |
|---|---|---|---|---|
| Oestrone pM | 155 ± 92 | *113 ± 60·9 | *78 ± 52 | *74 ± 44 |
| % of baseline oestrone | | 42 ± 18 | 43 ± 19 | 41 ± 20 |
| DHEAS (μM) | 1·81 ± 1·12 | *0·14 ± 0·08 | *0·15 ± 0·13 | *0·1 ± 0·1 |
| % of baseline DHEAS | | 16 ± 10·9 | 10 ± 8 | 8 ± 7 |

* $P = < 0{\cdot}01$ by paired $t$ test compared to baseline levels.

There were no significant differences between 500, 750 or 1000 mg/day AG in effects on oestrone or DHEAS. 26 patients were assessable for response. 6/8 responders had oestrone <40 pM but only 2/18 non-responders had such levels ($P < 0{\cdot}01$). There was no difference in DHEAS suppression. These results show that increasing doses of AG above 500–750 mg did not suppress oestrone further, and suggest that an extra-adrenal source of oestrone is responsible for higher oestrone in non-responders. AG could be combined with an anti-oestrogen in this group of patients to try and increase their response.

### FAILURE OF CYTOTOXIC-DRUG THERAPY TO REDUCE THE COLONY FORMING ABILITY OF A HUMAN BREAST-CANCER CELL LINE.

H. W. van den Berg†, R. Clarke* & R. F. Murphy*, *Dept. Therapeutics and Pharmacology† and Biochemistry*, The Queen's University of Belfast*

Prior to investigating possible interactions between hormone and cytotoxic-drug therapy using human breast-cancer cells growing *in vitro* as a model, we have assessed the ability of cytotoxic drugs alone to kill such cells. Human breast-cancer cells (MCF-7) were exposed to vincristine, 5-fluorouracil and methotrexate. Clinically achievable extracellular drug concentrations and exposure times were used, these being derived from parallel human pharmacokinetic studies. End points used for determining drug effects were inhibition of incorporation of appropriate precursors into nucleic acids, reduction of colony-forming ability and slowing of population growth rate. Vincristine ($10^{-9}$–$10^{-7}$M), was active by all these criteria following a 24h exposure. In contrast, treatment with 5-fluorouracil (up to $3{\cdot}5 \times 10^{-6}$M) or methotrexate ($10^{-8}$–$10^{-4}$M) for the same period has little effect on colony-forming ability, though DNA synthesis was markedly inhibited. Inhibition of nucleic-acid synthesis correlated with an initial cessation of population growth, but within 3–12 days after treatment control growth rate resumed. Similar results were obtained if cells were grown in medium containing dialysed serum. Exposure of MCF-7 cells to higher concentrations of 5-fluorouracil for 1 h produced a more marked reduction in population growth rate suggesting that extracellular concentrations of this drug mimicking those achievable *in vivo* following i.v. bolus injection, were more effective than those pertaining to 24h infusions. Nevertheless, both 5-fluorouracil and methotrexate appear to exert a cytostatic rather than a cytotoxic effect on MCF-7 cells.

### SERUM FUCOSE IN STAGED BREAST-CANCER PATIENTS.

B. Cantwell, J. J. Fennelly & C. Ryan, *Medical Oncology Service, University College, Dublin and St Vincent's Hospital, Dublin, Ireland*

The concentration of protein-bound fucose in the sera of 144 normal subjects and 56

subjects with staged breast cancer was measured, in order to determine whether raised serum fucose levels in breast-cancer patients reflected tumour stage.

The method used for measuring protein-bound fucose in the sera was that of Dische & Shettles as described by Winzler (1955, *Methods Biochem. Anal.*, **2**, 294). Serum fucose levels were compared with concomitant estimate of CEA and ESR in breast-cancer patients and a positive significant correlation obtained.

When compared to normal subjects significantly higher serum fucose concentrations were found in breast-cancer patients, and showed correlation with tumour stage (UICC) but not specifically to axillary-node status. In 6 of 7 patients with advanced breast cancer who had had serial estimations of serum fucose, a falling level was noted in association with response to systemic treatments.

These results suggest that significantly high serum fucose levels occur in breast cancer and correlate with advancing stage of disease but not with nodal status. Serum fucose estimations are also of value in assessing tumour stage and response to therapy in breast cancer.

### OESTROGEN-RECEPTOR STATUS OF BREAST CANCER IMMEDIATELY BEFORE CHEMOTHERAPY AND RESPONSE TO TREATMENT.

J. F. Stewart, R. J. B. King & R. D. Rubens, *ICRF Breast Unit, Guy's Hospital, London*

There is uncertainty whether oestrogen receptor (RE) content influences the response of advanced breast cancer to chemotherapy. However, the several reports to date have used RE results obtained before the administration of endocrine treatment. It is possible that this treatment could alter receptor status and that this phenomenon could account for the varying results so far reported. Consequently, we have undertaken a prospective study in which REs have been measured immediately before chemotherapy. Patients who have had additive endocrine therapy in the preceding 4 weeks are excluded. Twenty-one patients in this study are so far available for analysis. The preliminary results are as follows:

| | RE < 5fmol/ mg protein | RE ⩾ 5fmol/ mg/protein |
|---|---|---|
| Number | 11 | 10 |
| Objective responses | 5 (45%) | 4 (40%) |
| No change | 3 (27%) | 1 (10%) |
| Progressive disease | 3 (27%) | 5 (50%) |

These early results suggest that RE status immediately before chemotherapy does not influence the frequency of response to chemotherapy, but this study continues.

### *CIS* - DIAMMINEDICHLOROPLATINUM (CDDP) BY INFUSION IN THE TREATMENT OF ADVANCED HEAD AND NECK CANCER.

A. L. Stewart, R. C. S. Pointon, P. M. Wilkinson, *Dept. of Radiotherapy and Oncology, Christie Hospital, Manchester*

The introduction of CDDP has added a further active agent to the treatment of head and neck cancer. The use of this agent is, however, often accompanied by severe gastrointestinal and renal toxicity. This may complicate its use in patients with these tumours, who are often old and in poor general condition. In an attempt to achieve useful tumour regression with acceptable toxicity, we have been evaluating CDDP used as a 24h infusion of 100 mg/m$^2$ in 4 l saline at 3 weekly intervals. 23 patients have received a total of 71 courses of treatment (range 1–7). All except one had received prior radiotherapy, and 17 had received prior chemotherapy with one or more agents. 2 patients had only received one course of CDDP and were not considered assessable for response. No patient achieved a complete response, but 9 (42%) achieved > 50% partial remission. The median duration of partial response was 24 weeks. Toxicity was acceptable, with only one patient declining further treatment. No impairment of renal function was seen after less than 6 courses of therapy, and although most patients experienced some nausea and vomiting, only 5 (22%) described this as severe. This study has demonstrated that CDDP is an effective agent with acceptable toxicity when used as a 24h infusion in the management of head and neck cancer. It should now be considered for use as the initial chemotherapy for recurrent head and neck cancer, and the combined approach of radiotherapy with CDDP should be evaluated as initial definitive therapy.

**PHARMACOKINETIC EVALUATION OF METHOTREXATE IN THE MANAGEMENT OF ADVANCED HEAD AND NECK CANCER.** P. M. WILKINSON, A. L. STEWART, J. MARGISON & R. C. S. POINTON, *Dept of Radiotherapy and Oncology, Christie Hospital, Manchester:* S. B. LUCAS, *Dept Medical Computation, Manchester University*

Methotrexate (MTX) (100 mg/m$^2$) was administered as an i.v. bolus every 14 days to 47 patients with advanced head and neck cancer. Tumour regression was observed in 24/47 (51%) and in 5 (10%) this was complete. The median duration of response was 20 weeks; 2 of the complete responders are alive and disease free 16 and 20 mths after discontinuing drug therapy. Toxicity was mild and acceptable, the commonest side effect being mucositis, which was observed in 12% of treatment cycles. There was a significant correlation for both response and toxicity with the area under the third phase of the concentration-time curve, and an inverse correlation between response and urinary MTX excretion during the first 24 h after drug administration. The metabolite 7-OH MTX was present in serum 2 h after drug administration, and attained peak levels at 6–8 h. No kinetic parameter could be identified that significantly correlated with either response or toxicity. Despite extensive pharmacokinetic analysis it was not possible to produce a reliable model that can identify those patients most likely to benefit from therapy.

**TREATMENT OF ADVANCED HEAD AND NECK CANCER; SYNCHRONOUS THERAPY WITH METHOTREXATE AND IRRADIATION.** R. C. S. POINTON, A. L. STEWART, R. D. HUNTER & P. M. WILKINSON, *Dept of Radiotherapy and Oncology, Christie Hospital and Holt Radium Institute, Manchester*

One option to improve survival in Stage III and IV head and neck cancer is to increase the efficacy of local irradiation by means of drug therapy. There is evidence that Methotrexate (MTX) can act as a radiosensitizer in addition to its known cytotoxic effects. Fifty patients with head and neck cancer (16% Stage III and 84% Stage IV) were treated with radical irradiation (15–16 fractions over 21 days, 4·0–52·5 Gy) concurrently with MTX 100 mg/m$^2$ per i.v. bolus (Days 0 and 14). The first 14 patients received one dose, the remainder both. The minimum duration of follow-up is 2 years. Complete resolution of disease was observed in 24 patients (50%); 4 patients have subsequently relapsed and died (10, 14, 15 and 29 mths), and 1 is alive with disease (25 mths). Toxicity included exaggerated skin reaction (30%), increased mucosal reaction (36%), delayed mucosal healing (50%) and myelo suppression (18/86 courses, 1 fatal). Most exaggerated reactions were predictable by pre-treatment assessment and 24h serum MTX concentrations, however in some this was unpredictable and the precise explanation is at present unclear. These results suggest that synchronous therapy may be superior to radiation alone, and this hypothesis is currently being tested at this Institute by an appropriate clinical trial.

**QUANTITATIVE ASPECTS OF COMBINED INFUSIONAL 5-FU AND RADIATION IN ADVANCED CANCER.** J. E. BYFIELD, *University of California, San Diego, California, U.S.A.*

Our clinic has evaluated infusional 5-FU (72–120 h) coupled with coincident X-ray therapy (XRT) on a fortnightly basis for advanced epithelial cancers (head, neck, lung, GI, and anus). The program is based on pre-clinical studies which have shown that 5-FU and XRT are synergistic only when (a) 5-FU is present post-XRT for $\geq$24 hours and (b) the extra-cellular level of 5-FU is $\geq$400 ng/ml (5 human tumour-cell lines). Pharmacokinetic studies in humans have shown that 5-FU catabolism can be saturated at high 5-FU infusional loads and the serum level then "set" by appropriate infusion rates. Clinical limiting toxicity for 5-FU changes from marrow depression to stomatitis at infusion durations $\geq$72 h. The onset of stomatitis can be closely predicted by the serum 5-FU levels (on a concentration $\times$ time basis). Phase I–II studies of this combination in the above tumours are nearly complete and suggest enhanced response rates and duration of response in several epithelial tumours. The programme is especially promising in its effects on squamous-cell cancers of the head and neck, oesophagus and anus.

In addition it can be combined with permanently implantable arterial infusion pumps for localized infusions (liver, limbs, etc.). In this programme marrow toxicity is seldom a major problem, though idiosyncratic reactions are occasionally seen. 72h infusions appear ideal for combination with XRT.

**A MODIFICATION OF MEGA-DOSE METHOTREXATE THERAPY WITH MINIMAL TOXICITY AND APPARENTLY MAINTAINED THERAPEUTIC EFFICACY IN VARIOUS MALIGNANCIES.** S. M. CRAWFORD, J. A. COX & R. L. TURNER, *Bradford Royal Infimary, Bradford*

Methotrexate (MTX) therapy in the dose range 1–10 g/m$^2$ was introduced in an attempt to induce penetration of the drug across the blood brain barrier and into large tumour masses with poor vascularity. This dose of MTX has usually been administered by i.v. infusion over 4–6 h. Calcium folinate rescue has commonly been started 4–6 h after completion of the infusion. Since MTX is S-phase specific, this early reversal must mitigate against its therapeutic effect.

In this study 130 courses of treatment were given to 26 patients. We have found that by administering a dose of 1·5 g/m$^2$ over 6 h followed by calcium folinate rescue at 24 h after the start of the infusion in patients receiving intensive alkalinization of urine over 3 days, haematological, hepatic and renal toxicity were minimal. Serum MTX levels were 1 $\mu$M at 48 h in only 13 courses and in most of these, further folinate rescue prevented serious toxicity.

Best results were obtained in the non-Hodgkin lymphoma, squamous-cell head and neck carcinomas and neurofibrosarcoma. This regime cost much less than conventional high-dose MTX and folinic acid rescue.

**A PROSPECTIVE RANDOMISED STUDY OF *CIS* PLATINUM AS A SINGLE AGENT AND IN COMBINATION WITH CHLORAMBUCIL IN ADVANCED OVARIAN CARCINOMA.** S. R. KANKIPATI & E. WILTSHAW, *Institute of Cancer Research, Royal Marsden Hospital, London*

Experience at the Royal Marsden Hospital has shown that in patients with ovarian cancer FIGO stages III and IV, together with patients having recurrent disease following radiotherapy, cisplatin + chlorambucil + Adriamycin is no better than cisplatin + chlorambucil. These patients now have more than a 4-year follow-up.

From March 1979 to November 1980 inclusive 94 patients were entered into a new study comparing chlorambucil + cisplatin (Regimen B: cisplatin 20 mg/m$^2$ i.v. Day 1 + chlorambucil 0·2 mg/kg/day p.o. Days 2–8 for 12 courses) with cisplatin alone (Regimen D: cisplatin 100 mg/m$^2$ i.v. with hydration for 5 courses followed by cisplatin 20 mg/m$^2$ i.v. for 7 courses).

All good responders were subjected to second-look laparotomy or laparoscopy. Complete response rates were 22·9% for B and 15% for D, while overall response rates were 66·6% and 74% respectively.

The probability of surviving 12 months was 57% and 35% at 24 months for both regimens.

Patients with complete remission had 100% survival after treatment B and 83% after D at 22 months, whereas patients who had partial response had a survival of 45% for B and 70% for D at 22 months.

It is concluded that in the short term, D is probably more effective than B but also more toxic.

**SINGLE-AGENT *CIS*-DICHLORODIAMMINE PLATINUM (II) IN PREVIOUSLY UNTREATED PATIENTS WITH OVARIAN CARCINOMA.** A. HOWELL, C. E. NEWMAN, K. K. CHAN, G. D. NEWSHOLME, S. R. SMITH & R. A. HURLOW. *Departments of Medicine, Surgery, Radiotherapy and Obstetrics and Gynaecology, University of Birmingham, Queen Elizabeth and Dudley Road Hospitals, Birmingham, U.K.*

Five courses of CDDP (100 mg/m$^2$) were given to 25 patients with Stages IIb, III and IV ovarian cancer. Eighteen patients had second-look laparotomy at the end of treatment and 7 were evaluated by clinical means alone. In the surgical cases 6 (33%) had a complete remission, 9 (50%) a partial remission and 3 (17%) had progressive disease. After completion of surgery, 16/18 (89%) of patients were in complete remission. When the 6 patients not operated upon were included with the assessment of the surgical cases after surgery, 19/25 (76%) had a complete response. One patient could not complete treatment because of tinnitus, and two others developed a reversible peripheral neuropathy. CDDP is highly active as a single agent at this dosage in previously untreated patients with late-stage ovarian cancer.

**TRIAL OF AN AROMATIC RETINOID (AR) IN PATIENTS WITH SOLID TUMOURS.** G. J. S. RUSTIN & K. D. BAGSHAWE, *Department of Medical Oncology, Charing Cross Hospital, London*

Retinoids can inhibit growth of several experimental tumours and stimulate differentiation of mice embryonal carcinoma cells. The effect of hypervitaminosis A induced by an aromatic retinoid (RO 10-9359) was assessed in 18 patients with advanced measurable cancer. Patients received AR for a minimum of 4 weeks. Hypervitaminosis A was judged to be present when the patients exhibited signs such as cheilitis, and was maintained by 25–50 mg of AR per day. All patients had received prior cytotoxic chemotherapy, and their types of tumour were metastatic testicular teratoma (6), ovarian adenocarcinoma (4), metastatic melanoma (2), bronchial adenocarcinoma (1), breast adenocarcinoma (1), colonic adenocarcinoma (1), uterine leiomyosarcoma (1), diffuse lymphocytic lymphoma (1) and Hodgkin's disease (1). There was disease progression whilst receiving AR in all patients except a female with lymphnode metastases from ovarian adenocarcinoma, who had a marginal response for 2+ months, and in a female with cerebral metastases from malignant melanoma. In the latter patient there was no increase in number or size of brain metastases whilst she received AR for 9+ months. In the teratoma patients 3 showed a rise in $\beta$-hCG and 3 a rise in AFP during AR therapy. One teratoma patient had a laparotomy before and a thoracotomy after 9 weeks of AR. Histology showed similar undifferentiated tumour at both operations. Although hypervitaminosis A was well tolerated in these patients, it only stabilized disease in 2/18 patients.

**PRELIMINARY COMMUNICATION ON THE PHARMACOKINETICS OF HUMAN LYMPHOBLASTOID INTERFERON (HLBI) GIVEN BY I.M. INJECTION.** T. J. PRIESTMAN, M. D. JOHNSTON & P. D. WHITEMAN, *Wellcome Research Laboratories, Beckenham, Kent*

The object of this study was to monitor the blood levels of HLBI over a 24h period in 4 patients with advanced malignant disease. Interferon (IF) activity was measured in a bioassay recording the degree of inhibition of Semliki forest virus growing in V3 cells. All patients received 4 to 5 Mu/m$^2$ of Wellcome HLBI (sp. act. $>4\times10^7$ u/mg protein), 2 patients had been given 2·5 Mu/m$^2$ daily for the previous 5 days and 2 had not received IF for at least one week before. HLBI was given by i.m. injection into the gluteus muscle at 09.00. The pattern of response was similar in all patients, peak blood levels being reached at 4–6 h and declining thereafter with an apparent half-life of $\sim$12 h. The relatively slow fall was probably mainly due to continuing absorption from the i.m. depot, and the true elimination half-life may be much shorter. There was still some IF present at 24 h, suggesting that daily dosing might lead to some accumulation. This was reinforced by the finding that the previously treated patients had higher initial levels and higher values throughout the study, peak levels in these 2 being 300–350 u/ml compared to 180 u/ml in the other 2 patients. In one patient a sample of cerebrospinal fluid showed no detectable IF activity at a time when the blood level was 160 u/ml. The pattern of response and blood levels seen in this study are similar to those reported with equivalent doses of leukocyte interferons.

**RESULTS OF TREATMENT WITH A MULTIPLE DRUG SCHEDULE (MDS) IN 26 PATIENTS WITH SOFT-TISSUE SARCOMA.** R. STUART-HARRIS, E. WILTSHAW, C. HARMER, A. MCKINNA & S. CONINX, *Royal Marsden Hospital, London*

Between 1970 and 1980 26 patients with advanced soft-tissue sarcoma were treated with cyclophosphamide (600 mg/m$^2$ max 1 g) 5-fluorouracil (500 mg/m$^2$, max 1 g),Vincristine (1 mg/m$^2$, max 2 mg), actinomycin-D (0·6 mg/m$^2$, max. 1 mg) and methotrexate

(200 mg). The methotrexate was given as a 24h infusion and followed by folinic-acid rescue. Treatment was repeated every 28 days.

5 patients had local disease, 21 had metastatic or locally recurrent disease. There were 2 complete and 5 partial responses (overall response rate 27%). One complete responder and 3 partial responders have relapsed, average duration of response being 9 months. One complete responder remains disease free more than 8 years after treatment. Two partial responders have been converted to complete remission, one with radiotherapy, one with radiotherapy and surgery.

The regime is generally well tolerated, though nausea, vomiting and thinning of the hair may occur.

**ROLE OF PULMONARY FUNCTION TESTS IN THE PREVENTION OF BLEOMYCIN PULMONARY TOXICITY DURING CHEMOTHERAPY FOR METASTATIC TESTICULAR TERATOMA.** H. H. LUCRAFT*, P. M. WILKINSON & T. B. STRETTON†, *Christie Hospital and Holt Radium Institute, Manchester, and †Manchester Royal Infirmary*

36 men were treated for metastatic testicular teratoma with 4 courses of chemotherapy each containing 90 mg bleomycin (BLM). Routine pulmonary-function tests (PFTs) were performed before each chemotherapy course, to determine their value in detecting early BLM pulmonary toxicity at this dose level. PFTs were repeated 2–5 years after completion of chemotherapy in 10 disease-free survivors. Analysis of changes in individual PFT values showed a fall in the carbon monoxide diffusing capacity ($DL_{co}$) after 90 mg BLM ($P<0.002$). The $DL_{co}$ remained depressed with subsequent doses of BLM, but there was no further statistically significant fall. There was no statistically significant change in any other PFT. Late PFT values showed no significant change. There were no cases of BLM pulmonary toxicity detected. There was no correlation between changes in the visible extent of metastases as assessed from the chest radiograph and changes in the PFTs. It was concluded that routine PFTs are unnecessary if the total BLM dose >360 mg, unless there are particular risk factors such as previous chest radiotherapy or age >70 years.

**MANAGEMENT OF PSYCHOLOGICAL STRESS IN CANCER PATIENTS: AN ALTERNATIVE APPROACH.** S. BINDEMANN, K. C. CALMAN, R. A. V. MILSTED & J. M. TROTTER, *Department of Clinical Oncology, University of Glasgow*

There exists at present no incontrovertible scientific evidence that prognosis in the cancer patient is affected by emotional factors. However, there is now considerable evidence in support of the hypothesis that "quality of life will be enhanced by intensive psychological support in the form of directive therapy and antogenic training". (Simonton & Simonton, 1975, *J. Transper. Psych.*, **7**, 29; Meares, 1979, *The Practitioner*, **222**, 119). A group of cancer patients with advanced disease who were all currently receiving cytotoxic chemotherapy were selected on the basis of manifestly high anxiety reaction and/or moderate to severe depression. All patients admitted to the study had agreed to procedures which had been carefully described and explained to them. The value of therapy was assessed subjectively by patients and therapist. Objective evaluation was attempted by means of questionnaire from patients' relatives and from members of the hospital's medical and nursing staff. Consistently high levels of agreement were obtained, which referred to rapid improvement in patients' emotional state. This condition was maintained without relapse in >80% of all members of the group. Such results suggest that therapy, which involves the use of suggestion together with a degree of light hypnosis, is of value in relieving psychological and psychosocial problems associated with malignant disease. Further trials designed to compare results obtained by this method, with results obtained by means of similar procedures involving psychophysiological feedback techniques, are currently being carried out as a stress-reducing adjunct to primary cancer therapy.

**NUTRITIONAL ABNORMALITIES IN CANCER PATIENTS WITH WEIGHT LOSS.** J. M. TROTTER, P. BOYLE, J. MCALLISTER, K. C. CALMAN & S. B. KAYE, *Departments of Clinical Oncology and Biochemistry, Gartnavel General Hospital, Glasgow, and Cancer Surveillance Unit, Ruchill Hospital*

A group of 52 cancer patients with weight loss (>10% over 6 months or 5% over one month) were assessed nutritionally by measuring anthropometric data (mid-arm muscle circumference, MAMC; triceps skin-fold thickness, TSFT; weight and percent weight loss), plasma proteins (albumin, total protein, transferrin, pre-albumin, retinol-binding protein), serum vitamin A and plasma zinc. Mean weight loss was 21·2%, with 53·1% of patients having >20% loss of weight. Mean survival in 44 deceased patients was 2·8 months, and only 6 patients in this advanced-disease population survived >6 months. Patients were evaluated together and in 3 groups: lung cancer (11), gut cancer (18) and miscellaneous (23). There were no significant differences between the 3 groups with respect to age, sex distribution, anthropometric data or plasma proteins. All subsequent data are therefore presented for the group as a whole. Hypoalbuminaemia (82·7%) and low retinol-binding protein (73·9%) were common findings, as was low plasma zinc (97·8%). Of interest is the lack of correlation between those plasma proteins measured, and also between the biochemical and anthropometric data. In addition, none of the tests of nutritional status, including albumin, correlated with survival. It is concluded that in advanced malignant disease, nutritional deficiencies are compound. Anthropometric data alone provide an insufficient evaluation of nutritional status, and selected vitamins and minerals also need monitoring. The data also suggest that the standard biochemical tests of nutritional deficiency used for uncomplicated protein–energy malnutrition, particularly plasma protein measurements, are less applicable in malignant disease. Further analysis using these guidelines in patients with early disease may provide information of therapeutic and prognostic importance.

**DIETETIC EVALUATION OF CANCER PATIENTS.** J. M. TROTTER, J. DUFFY, K. C. CALMAN & J. C. WILLOX, *Departments of Clinical Oncology and Dietetics, Gartnavel General Hospital, Glasgow*

Nutritional deficiencies are common in the cancer patient and abnormalities of carbohydrate, lipid, mineral and vitamin metabolism have been described (Theologides, 1979, *Cancer*, **43**, 2004). Biochemical screening for all potential nutritional abnormalities is expensive and often unrewarding. Manual analysis of the composition of the diet is tedious. In this study, a 24h dietary-recall history was obtained from oncology outpatients, and food composition was analysed by a computer program based on the McCance–Widdowson tables of food composition. Two groups were evaluated: those with symptomatic anorexia and a second group, who had not previously been seen by the dietician, selected at random from the outpatient population. A total of 59 patients were assessed and 15 analyses were repeated on a second occasion. Of the 33 patients selected at random, 13 (39·4%) were eating less than the recommended daily intake, whereas this applied to all of the 26 symptomatically anorectic patients. Commonest food aversions in the anorectic patients were meat (11), tea (7) and coffee (4). Cravings for cheese (5) and eggs (2) may represent substitution of a more palatable protein source for some patients. Dietary analysis revealed frequent deficiencies of vitamins (especially thiamine, $B_6$, C, A and folic acid) and of minerals (particularly Zn, K and Mg). Computer analysis of food intake was found to be rapid and easy and of immediate potential practical benefit, by pinpointing specific dietary deficiencies. In addition, about 20% of asymptomatic patients were found to have unsuspected dietary deficiencies.

**SPINAL STABILISATION IN SECONDARY MALIGNANCY.** A. J. BANKS & C. S. B. GALASKO, *Department of Orthopaedic Surgery, University of Manchester*, and E. DERVIN, *Department of Aeronautical and Mechanical Engineering, University of Salford*

Back pain is commonly seen in patients with malignant disease. In 10% of such patients the pain is due to instability of the spine as a result of the underlying bone destruction (Galasko & Sylvester, 1978, *Clin. Oncol.*, **4**, 273). This complication is comparable to the development of a pathological fracture in the appendicular skeleton. Previous attempts to stabilize the spine have only been partially successful, because the apparatus used has been designed for the stabilization of a scoliotic spine.

This paper describes the development of a new rod which has been designed specifically for the stabilization of malignant spines. Preliminary laboratory tests have demonstrated that the prosthesis should be inherently strong without the need for supplementary cement or bone graft, and should be fixed to the spine in an unstressed condition. These criteria were met by using a square-section bar which could be secured to the spine at multiple levels.

To date 11 patients have been treated. In all patients the extremely severe pre-operative pain, which was exacerbated by any movement, was relieved. Prior to surgery all patients were confined to bed. Following surgery they were all mobilized and could sit, stand or walk without pain. Although some of the patients have now died from their disease, the longest survivor is still pain-free and mobile two years after operation. All patients achieved good palliation.

**SELECTION OF METASTATIC VARIANTS FROM N-METHYL-N-NITROSOUREA-INDUCED RAT MAMMARY TUMOURS.** J. C. Williams, B. A. Gusterson & R. C. Coombes. *Ludwig Institute for Cancer Research (London Branch), Royal Marsden Hospital, Sutton, Surrey*

N-methyl-N-nitrosourea (MNU) has been used to induce mammary adenocarcinomas in inbred strains of female rats; these tumours are hormone sensitive but do not spontaneously metastasize (Williams *et al.*, 1981, *J. Natl Cancer Inst.*, in press). Two methods have been used to derive spontaneously metastasizing tumours from MNU-induced primaries.

A cell line has been isolated *in vitro* from a mammary adenocarcinoma induced in female F344/N rats. This strain of cells forms colonies in lungs of syngeneic animals when injected i.v., and forms tumours when injected i.m., s.c. and into the mammary fat pad. Tumours growing in these 3 sites show a high incidence of metastasis to the lungs, and in the lymph nodes at lower incidence. A cell suspension obtained by enzymatic digestion of the same initial MNU-induced tumour formed lung colonies after i.v. injection. Colonies were tested for metastasis after reimplantation in the mammary fat pad. After 3 passages through the lungs a solid transplantable tumour has been obtained which metastasizes spontaneously to the lungs and lymph nodes. The tumours formed by the cell line and the transplantable tumours were histologically similar, showing glandular differentiation but including a spindle-cell component and areas of squamous differentiation. Metastases were morphologically similar. Both tumours and metastases contain measurable levels of cytoplasmic oestrogen receptor. These systems may therefore constitute a useful model for the study of metastasis.

**EARLIER DETECTION OF CIRCULATING TUMOUR CELLS AND METASTASES IN A HIGH-METASTASIS VARIANT OF LEWIS LUNG CARCINOMA.** M. Magudia, P. Whur, J. Roberts & D. C. Williams, *Cell Biology Unit, Marie Curie Memorial Foundation, Oxted, Surrey*

A stable line of Lewis lung carcinoma was maintained under a set of fixed conditions i.m. passage of cells from pooled primary tumours. A high-metastasis variant was obtained by repeating the procedure but substituting cells from lung metastases (Magudia *et al.*, 1980, *Dev. Oncol.*, **4,** 170) over 8 generations. In order to investigate the difference in metastatic potential, mice injected with one of the 2 lines were killed daily in groups of 5 for the next 26 days and the following parameters monitored: (1) primary tumor size, (2) number and size of overt metastases, (3) total and differential WBC counts for quantitating circulating tumour cells.

The primary tumours of the 2 lines grew at the same rate ($P > 0{\cdot}05$) as did the metastases ($P > 0{\cdot}7$). By Day 26 there were $134 \pm 12$ metastases in the variant compared to $66 \pm 11$ in the stable line ($P < 0{\cdot}01$). They appeared 5 days earlier (Day 5) in the variant and the quantitative differences in metastases were solely attributable to the time factor. Tumour cells in venous blood were detected 8 days earlier in the variant (Day 5) than in the stable line. By Day 20 both had reached a plateau of $\sim 2{\cdot}5 \times 10^5$ cells/ml of blood.

Our results suggest that the higher metastatic potential of the variant is due to the earlier establishment of metastases in the lungs, which may in turn correlate with the earlier appearance of tumour cells in the circulation.

### PLOIDY DISTRIBUTION OF TUMOUR CELLS FROM INDUCED AND SPONTANEOUSLY ARISING METASTASES.

J. REEVE & P. TWENTYMAN, *MRC Clinical Oncology and Radiotherapeutics Unit, Cambridge*

A radiation-induced sarcoma (RIF-1) of the inbred C3H/Km mouse has been used in studies designed to evaluate tumour-cell heterogeneity with respect to a variety of parameters including metastatic potential. The tumour is non- or minimally immunogenic in its syngeneic host, and grows either *in vivo* as a solid tumour or *in vitro* as a monolayer, clones or, under appropriate conditions, as multicellular tumour spheroids. Flow-cytometric analysis of both *in vivo*- and *in vitro*-derived tumour cells has shown that the RIF-1 parent tumour is composed of diploid and tetraploid sub-populations of cells, each being capable of independent proliferation. This finding has been confirmed by chromosome analysis.

Following i.v. injection of $10^5$ tumour-derived cells into mice, artificial "metastases" arise at a variety of body sites, including lung, ovary and chest wall. Flow-cytometric analysis of these "metastatic" sublines revealed that, unlike the parent tumour, all had a single level of ploidy, which remained stable throughout 2 successive *in vivo* or 4 successive *in vitro* passages. Recently, data have been obtained for the ploidy distribution of spontaneously arising metastases after removal of a large primary tumour. Early results again indicate that these metastases are composed of cells of a single level of ploidy. This finding may indicate that, in this tumour system, spontaneous metastasis is a clonal event.

### TUMOUR NECROSIS FACTOR FROM THE RABBIT. *IN VIVO* ACTIVITY AND SITES OF SYNTHESIS.

N. MATTHEWS, *Medical Microbiology Department, Welsh National School of Medicine, Cardiff*

Tumour-necrosis factor (TNF) was first described by Carswell *et al.*, 1975 (*Proc. Natl Acad. Sci*, **72,** 3666) as a factor present in the serum of animals with shock induced by i.v. injection of BCG and endotoxin 2 weeks apart. This serum induced necrosis of some transplantable tumours and was cytotoxic *in vitro* to certain tumour cell lines. As partially purified (×30) mouse TNF had both activities it was suggested that the same factor was responsible for both activities.

We have now shown that μg amounts of rabbit TNF, purified 1000–2000 fold on the basis of *in vitro* activity, can induce necrosis of a transplantable fibrosarcoma in mice.

The cellular source of TNF appears to be the mononuclear phagocyte. Comparison of various tissues from normal and BCG-injected rabbits for capacity to synthesise TNF *in vitro* has shown: (1) increased numbers of mononuclear phagocytes in BCG rabbits; (2) BCG-mononuclear phagocytes have much increased TNF-synthetic capacity; (3) the main sources of TNF are lungs, liver and blood.

### THE PRESENCE OF A TUMOUR PROTECTS MICE UNDERGOING A GRAFT VERSUS HOST REACTION (GVHR) AGAINST THE ACTION OF LIPOPOLYSACCHARIDE ENDOTOXIN (LPS).

D. B. PALMER, T. WHITMARSH-EVERISS & M. O. SYMES, *Department of Surgery, University of Bristol*

(A×CBA)$F_1$ mice injected with A (immune to CBA) spleen cells showed less GVHR (as judged by spleen weight) if an $F_1$ hybrid tumour was present (Whitmarsh-Everiss & Symes 1981, *Br. J. Cancer*, in press). Macrophage reactivity is increased in mice undergoing GVHR (Howard, 1961, *Br. J. Exp. Pathol.*, **42,** 72) and such activated macrophages show increased sensitivity to the action of LPS as judged by a rise in enzyme levels after endotoxin administration (Ferluga & Allison, 1978, *Lancet*, ii, 610; Bradfield *et al.*, 1980, *Br. J. Cancer*, **42,** 900).

In groups of 4–6 $F_1$ mice GVHR was induced in the presence of an $F_1$ tumour, after tumour resection, or in animals not receiving a tumour transplant. Thirteen days later, half the animals in each group received 25 μg LPS i.v., and all animals were killed 24 h later. The plasma levels of amino aspartate transaminase (AST) ornithine carbamoyl transferase (OCT) and β-galactosidase were determined. Injection of LPS produced a rise in AST and OCT levels in all groups of animals.

| | Enzyme levels (iu/l) | | |
|---|---|---|---|
| | AST | OCT | β-galactosidase |
| No tumour | 76·7 ± 28·6 | 11·86 ± 1·56 | 0·16 ± 0·04 |
| GVHR | 231·7 ± 31·2* | 13·65 ± 2·20 NS | 0·45 ± 0·05* |
| Tumour + GVHR | 140·0 ± 35·0 NS | 3·91 ± 2·20** | 0·27 ± 0·06 NS |
| Tumour resected + GVHR | 373·7 ± 35·0* | 14·75 ± 2·70 NS | 0·51 ± 0·06* |

Sig *vs* No tumour: $^*P < 0{\cdot}001$; $^{**}P < 0{\cdot}01$. NS = Not significant.

In the animals receiving LPS, GVHR was associated with a rise in the level of all 3 enzymes. The enzyme levels were reduced when GVHR was ongoing in the presence of a tumour, an effect abolished by resection of the tumour before the GVHR arose.

**SPECIFIC AND NON-SPECIFIC CELLULAR RESPONSES MODULATING GROWTH OF RAT HEPATOMA D192 AND THEIR MANIPULATION IN IMMUNOTHERAPY.** J. A. Jones, G. Robinson & R. W. Baldwin, *Cancer Research Campaign Laboratories, University of Nottingham*

The role of specific immune responses elicited against tumour-associated antigens has been evaluated using an aminoazo-dye-induced hepatoma D192 transplanted into syngeneic WAB/Not rats. Examination of the requirements of tumour-cell vaccines for generating a specific tumour immunity demonstrated that viable tumour cells admixed with BCG so as to prevent progressive tumour growth provided the most effective tumour-rejection response. Under these conditions, effective therapy could be initiated up to 6 days after tumour challenge, so causing rejection of tumour at a contralateral site. A major component of the host response generated by tumour-cell vaccines involved the production of sensitized lymphocytes in the lymph nodes draining the vaccine, since systemic anti-tumour responses were abrogated by lymphadonectomy. In contrast, removal of the lymph node draining the tumour-challenge site had no effect. Lymphocytes stimulated by tumour-cell vaccines transferred tumour immunity, this being specific for the immunizing tumour. Vaccine treatment also leads to the generation of natural killer cells, suggesting that specifically sensitized effector cells are necessary to elicit a tumour rejection response, even though these cells may not be directly cytotoxic.

**SPECIFIC AND NON-SPECIFIC LYMPHOCYTE CYTOTOXIC FUNCTION IN COLON CARCINOMA.** P. Gallagher*, B. M. Vose, M. Moore & P. F. Schofield*, *Department of Immunology, Paterson Laboratories, Christie Hospital and Holt Radium Institute, and *Department of Surgery, Withington Hospital, Manchester*

The cytotoxic activity of peripheral blood (PBL), lymph node (LNC) and tumour-infiltrating lymphocytes (TIL) from 47 patients undergoing surgery for colon carcinoma (Duke's Stage A, 1 patient; B, 24; C, 15 and C with metastases, 7) was examined in short-term Cr-release assays, against fresh autologous tumour cells, allogeneic colon cells and the erythroleukaemia cell line, K562. Cytotoxicity against autologous cells was detected in at least one effector population in 23/47 (49%) patients, with overall frequencies which did not differ significantly for patients in different Duke's stages of disease. By contrast, lysis of allogeneic tumour cells was an infrequent event (< 11%) regardless of the effector population to which they were exposed. Cytotoxicity against K562, cells highly sensitive to NK activity, though variable, was detected in the PBL of normal donors (93%) and patients (83%), and among the latter showed no evidence of significant decline with advancing disease. However, LNC and TIL anti-K562 activity was detected only rarely (< 17%), agreeing with previous reports. There was no correlation between the activity of patients' PBL to lyse autologous tumour and K562 cells. The independence of these 2 cytotoxic functions was further explored in lymphocyte fractionation studies: autologous tumour killing was augmented in T-enriched PBL; whilst greatest anti-K562 activity resided in the corresponding non-T fraction. Lymphocyte cytotoxicity in colonic neoplasia is thus manifest in 2 apparently independent lymphocyte populations; a relatively specific killer T-cell population, detectable in PBL,

LNC and TIL, which is preferentially reactive with autologous cells; and a non-specific killer population, largely limited to PBL, with the properties of NK cells. The activity of neither population reflects the clinical status of patients with this disease.

### NK SENSITIVITY OF CELLS FROM PRIMARY AND METASTATIC DEPOSITS OF SPONTANEOUS TUMOURS.

G. R. FLANNERY, C. G. BROOKS, E. B. AUSTIN & R. W. BALDWIN, *Cancer Research Campaign Laboratory, University of Nottingham*

If natural killer (NK) cells play a role in immunosurveillance it might be expected that during the metastatic process, selection would occur for tumour cells with reduced NK sensitivity. This hyposthesis was tested in the rat by measuring the NK sensitivity of cells freshly isolated from metastases of 3 syngeneic transplanted spontaneous mammary carcinomas. Lysis was measured in a 6h Cr-release assay using normal syngeneic spleen cells as effectors. Cells from 6/13 draining lymph-node metastases and from 5/8 lung metastases were significantly ($P<0{\cdot}01$) less sensitive to syngeneic NK cells than cells from the corresponding primary tumours, but pericardial metastases showed normal or raised sensitivity. Cold-target competition assays indicate that the changes in NK sensitivity of metastatic variants were generally due to changes in intrinsic lysability rather than in NK target structure, since some of the most resistant preparations had normal competitive activity. When placed in culture, metastasis-derived cells regained normal NK sensitivity within a few days.

These studies show that during metastasis, selection for tumour cells expressing reduced NK sensitivity can occur, and the tissue distribution of metastases containing NK resistant cells suggests that this takes place primarily within the target organ rather than at the site of the primary tumour or in the blood stream. The results strengthen the hypothesis that NK cells play a role in immune surveillance, particularly in controlling the metastatic spread of cancer.

### THE CYTOTOXIC ACTIVITY OF TUMOUR-INTRINSIC AND PERIPHERAL-BLOOD LYMPHOCYTES AGAINST AUTOPLASTIC COLORECTAL CARCINOMA CELLS.

D. HEINEMANN, G. H. HUTCHINSON, M. O. SYMES & R. C. N. WILLIAMSON, *Department of Surgery, University of Bristol*

Tumour digests were prepared using collagenase and DNAse, from 16 colorectal cancers. Neoplastic cells were separated by centrifugation at 60 *g* for 10 min and tumour-intrinsic lymphocytes (TIL) by passage of the resulting supernatant through a nylon-wool column. Lymphocytes were also separated from peripheral blood (PBL) by density-gradient centrifugation, followed by passage of the cells through a nylon column. The neoplastic cells were labelled with 100 $\mu$Ci of $^{51}$Cr for 2 h, and then co-cultured with either TIL or PBL for 2 h to determine lymphocyte cytotoxicity.

In 11 of 16 patients PBL showed cytotoxicity, whereas in only 5/16 cases were TIL reactive. However, in 11 cases the cytotoxicity of TIL was compared before and after washing the lymphocytes $\times 6$ in Medium 199. Cytotoxicity of unwashed TIL was found in 3/11 patients, whereas for washed TIL the proportion was 9/11 ($P<0{\cdot}02$). Furthermore at the 10:1 and 20:1 effector target ratios the level of cytotoxicity using washed cells was greater ($P<0{\cdot}05$ and $P<0{\cdot}001$ respectively). The greater cytotoxicity of PBL than TIL is correlated with a higher percentage of T cells in the PBL population ($50{\cdot}6 \pm 15{\cdot}5$ *vs* $18{\cdot}2 \pm 13{\cdot}6$; $P<0{\cdot}001$). Washing of TIL may remove tumour antigen which blocks cytotoxicity (Currie & Basham, 1972, *Br. J. Cancer*, **26,** 427).

### LYMPHOCYTE REACTIVITY TO TUMOUR-ASSOCIATED ANTIGENS OF HUMAN COLORECTAL CANCERS.

G. H. HUTCHINSON, D. HEINEMANN, M. O. SYMES & R. C. N. WILLIAMSON, *Department of Surgery, University of Bristol*

It has previously been found that lymphocytes separated from the tumour mass in patients with colorectal cancer, show only limited cytotoxicity on co-culture with $^{51}$Cr-labelled autoplastic tumour cells. Lymphocyte cytotoxicity was significantly increased by washing ×6 in Medium 199.

In 5 patients with a benign polyp in addition to colorectal carcinoma, unwashed lymphocytes from the polyp showed marked cytotoxicity towards cells of the carcinoma.

Incubation of washed lymphocytes, obtained from a carcinoma, in the patients' own plasma significantly reduced their cytotoxicity ($n=8$; $P<0{\cdot}001$). A similar reduction was seen after incubation in plasma from a second patient with colorectal cancer ($n=6$; $P<0{\cdot}001$). Plasma from a patient with carcinoma of the breast also abrogated cytotoxicity, but the effect was significantly less than for patients with colorectal cancer.

It is suggested that colorectal cancers possess a common tumour-associated antigen which blocks the cytotoxicity of lymphocytes by coating their membranes. The antigen can be removed by washing the lymphocytes. The antigen is also shed into the plasma, hence incubation of washed lymphocytes in same plasma abrogates their cytotoxicity. The antigen is present on the cells of polyps, but is less readily shed, hence unwashed lymphocytes from a polyp react to a concomitant carcinoma.

**IMMUNE-DIRECTED THERAPY OF A TRANSPLANTED RAT MAMMARY CARCINOMA OF SPONTANEOUS ORIGIN (SP4).** J. A. JONES & R. W. BALDWIN, *Cancer Research Campaign Laboratories, University of Nottingham*

A monoclonal antibody to a syngeneically transplanted tumour has been used as a carrier of a chemotherapeutic drug, to investigate its effect on *in vivo* tumour growth and host survival.

Monoclonal antibody (rat IgG2b, mouse $\kappa$ chain) was isolated from hybridoma supernatants by immunoadsorbent purification (Sepharose goat anti-rat Ig) and conjugated to Adriamycin *via* a dextran bridge. Sp4 tumour cells are susceptible to ADM *in vitro* as shown by colony inhibition and $^{125}$IUdR uptake. In preliminary experiments rats were implanted s.c. with $2\times10^4$ Sp4 cells in admixture with 1 $\mu$g ADM either free or conjugated to normal rat Ig or Sp4 MoAb (drug:protein molar ratio $\sim$15:1). Significant inhibition of tumour growth by the drug-MoAb conjugate was found.

Rats implanted s.c. with $2\times10^4$ Sp4 cells were treated i.p. 7 days later with similar conjugates, each rat receiving 10 $\mu$g drug at a molar ratio to protein of $\sim$20:1; up to 3 more treatments were given weekly. Tumour growth was retarded by free ADM and drug-NIg relative to untreated controls but the drug-MoAb group showed significant tumour inhibition and increased survival times. This was not seen when an irrelevant spontaneous tumour (Sp15) was used.

These findings indicate that a monoclonal antibody has potential in the systemic treatment of tumour growth when used as a carrier to therapeutic agents.

***IN VIVO* TUMOUR LOCALIZATION OF MONOCLONAL ANTIBODY TO A TRANSPLANTED RAT MAMMARY CARCINOMA OF SPONTANEOUS ORIGIN.** M. V. PIMM & R. W. BALDWIN, *Cancer Research Campaign Laboratories, University of Nottingham*

In view of the current interest in antibodies as carriers of diagnostic and therapeutic agents, studies have been carried out to examine the *in vivo* tumour-localizing potential of a monoclonal antibody (MoAb) to the transplanted rat mammary carcinoma Sp4 (Gunn *et al.*, 1980, *Int. J. Cancer*, **26**, 325).

Monoclonal antibody (rat IgG2b, mouse $\kappa$ chain) was isolated from hybridoma supernatants by immunoabsorbent purification (Sepharose goat anti-rat Ig) and labelled with $^{125}$I using Iodogen to 1$\mu$Ci/$\mu$g. On i.v. injection into rats with pulmonary growths of Sp4, labelled MoAb (0·5 $\mu$g/rat) showed greater pulmonary uptake than with lungs of normal rats, or rats with pulmonary growth of an unrelated tumour (sarcoma Mc7). Uptake of $^{125}$I normal Ig was not significantly increased in Sp4-bearing lungs. In rats with s.c. tumours, $^{125}$I-Sp4 MoAb (0·2 $\mu$g/rat) showed a 2–6-fold higher uptake in Sp4 growths (0·6% injected activity/g) than in lung, liver, spleen, kidney or heart (0·1–0·2% uptake/g) and this was maintained over 5 days' observation. There was no increased uptake into growths of other tumours (mammary carcinoma Sp15, hepatoma D192A, sarcomas Mc7 and Mc96A) and with $^{125}$I-Ig there was no more uptake into Sp4 growths than with the other tumours.

These findings indicate that a monoclonal antibody to a syngeneically transplanted tumour has *in vivo* localizing potential, and could be used for tumour detection and as a carrier for therapeutic agents.

**DEMONSTRATION OF ACTIVITY OF MONOCLONAL ANTIBODIES ON COLONIC TISSUE USING AN INDIRECT IMMUNOPEROXIDASE TECHNIQUE.** R. M. GRANT*, P. J. FINAN*, E. LENNOX† & N. M. BLEEHEN*, **MRC Clinical Oncology and Radiotherapeutics Unit, Cambridge, †MRC Laboratory of Molecular Biology, Cambridge*

Immunoperoxidase staining techniques are now widely used for localizing cellular antigen. Methods previously described (Heyderman & Munro Neville, 1977, *J. Clin. Pathol.*, **30**, 138) have used conventional antisera. The introduction of monoclonal antibodies to this technique may well allow for more specific localization of these antigens.

Using an indirect immunoperoxidase technique we have screened 22 rat monoclonal antibodies, isolated after immunization with human colonic carcinoma membranes (Takei & Lennox, unpublished), on 20 formalin-fixed paraffin-embedded specimens of colonic carcinoma. Two distinct patterns of activity have been noted. Three antibodies showed activity on all normal colonic epithelium, both as an intracellular granular stain and on the surface of the epithelial cells, filling the goblets and staining the mucus secretions. A further 2 monoclonal antibodies showed maximal activity in colonic tumours, with apical intracellular staining of the tumour cells and dense staining in the lumen of malignant tumour glands. This pattern of staining was present in 19 of the 20 tumour specimens. Further work is required to determine the exact nature of antigens demonstrated by this technique.

The immunoperoxidase technique is a suitable method for initial screening of monoclonal antibodies on tissues of interest. As more monoclonal antibodies become available this technique will allow for better localization of tumour antigens and potential marker substances.

**IDENTIFICATION OF ANTIBODIES TO HUMAN PANCREATIC CANCER CELLS IN IMMUNOCOMPETENT HAIRY LITTER MATES IMMUNIZED WITH SERUM FROM TUMOUR-BEARING NUDE MICE.** A. G. GRANT, D. DUKE & J. HERMON-TAYLOR, *Department of Surgery, St George's Hospital Medical School, London, and the Department of Cancer Chemotherapy, Imperial Cancer Research Fund, London*

Using the concept that tumour cells synthesize and release specific membrane-associated proteins during growth, antibodies have been raised in immunocompetent hairy litter mates to sera from nude mice bearing human pancreatic tumour. No antibodies were detected in tumour-bearing nude-mouse serum. Antisera raised against sera from 2 different pancreatic cancer xenografts showed a titre of activity $>1{\cdot}625$ against cultured pancreatic tumour cells by an $I^{125}$-binding assay. Five out of the 14 hairy litter mates immunized with serum from the same tumour (GER) produced antisera that bound more strongly to pancreatic cancer cells (binding ratio $>2$) when tested against human foetal pancreatic fibroblasts, normal and EBV-transformed lymphocytes, myeloid, lymphoblastoid, mammary and urinary bladder human tumour cell lines, and a murine tumour cell line. Binding ratios of $<2$ were found with a fibroblast cell line derived from the same pancreatic tumour (GF) and a colon tumour cell line HT-29. Adsorption of the antisera with CEA reduced the level of binding by 11–24% without affecting the specificity for pancreatic tumour cells. Immunofluorescent staining of pancreatic tumour sections indicated that the antibody was localized on the membrane of ductular epithelial cells. Challenge of immunocompetent mice using this procedure may provide a route to the production of antibody for the characterization of selected tumour components.

**LEVAMISOLE AS AN INHIBITOR OF ENDOGENOUS ALKALINE PHOSPHATASE (AP) IN IMMUNOHISTOCHEMISTRY WITH AP CONJUGATES.** B. A. J. PONDER & M. WILKINSON, *Institute of Cancer Research, Sutton, Surrey*

We wished to use AP conjugates to demonstrate H2 antigens in mouse tissue sections by immunohistochemistry. A major difficulty was to inhibit endogenous tissue AP without interfering with the specific staining. Standard methods, such as exposure of sections to 20% acetic acid, destroyed the antigens we hoped to demonstrate.

Levamisole (1-tetramisole) inhibits the non-intestinal form of AP, but is without effect on the intestinal form. We have exploited this difference by using conjugates made with calf intestinal AP. The AP staining is performed in the presence of 1 mmol/l levamisole, which inhibits endogenous enzyme in all tissues other than small intestine and stomach, without loss of specific staining by the conjugate.

**SEPARATION OF HUMAN BREAST-CANCER CELLS.** R. Buckman, D. P. Dearnaley, R. C. Coombes & A. M. Neville, *Ludwig Institute for Cancer Research (London Branch), Royal Marsden Hospital, Sutton, Surrey*

We have developed a simple and reliable rosetting technique to separate malignant breast epithelial cells from host stromal cells at various metastatic sites. The method has 3 applications.

(A) In order to increase the detection rate of malignant cells in marrow, we have obtained 6 aspirates from each patient. The large number of smears generated prompted us to develop a simplified screening procedure. The monoclonal antibody anti-HLe-1 (gift of Dr P. Beverley) (Bradstock *et al.*, 1980, *J. Natl Cancer Inst.*, **65,** 33) binds to most normal marrow cells, which are then removed by rosette formation. This method rosettes out an average of 95% (range 85–98% N = 10) of normal marrow cells, leaving the malignant cells in the layer which can then be examined with only one or two smears.

(B) In order to free the marrow of small numbers of malignant cells, we have used an antiserum to epithelial membrane antigen (Heyderman *et al.*, 1979, *J. Clin. Pathol.*, **32,** 35) to rosette out the malignant cells, and are currently investigating the effect of this procedure on marrow function *in vitro*.

(C) We have prepared pure tumour-cell populations from digests of involved lymph nodes removed at surgery. Using anti-HLe-1 and the anti-fibroblast monoclonal 86·3 (Edwards, 1980, *Cell Biol. Int. Rep.*, **10,** 917) we have obtained $10^5$–$10^6$ tumour cells from 6 nodes with no detectable contamination by host cells.

We feel that rosetting techniques using monoclonal and conventional antibodies may be useful in cell separation for diagnostic and other purposes.

**PERIPHERAL-BLOOD INVOLVEMENT (PBI) IN LYMPHOMA: DETECTION USING LECTIN BINDING.** G. Blackledge, A. Morris, D. Crowther & J. Gallagher, *CRC Dept of Medical Oncology, Christie Hospital, Manchester*

PBI in lymphoma may be difficult to detect by morphological methods, since abnormal cells may resemble normal peripheral-blood lymphocytes (PBL). PBL have a characteristic lectin-binding pattern using fluorescent Concanavalin A (Con. A), *Lens culinaris* Lectin (LCA) wheat-germ lectin (WGA) and peanut lectin (PNA) when measured by flow cytometry. The PBL of over 60 patients, 13 with Hodgkin's disease and 47 with Non-Hodgkin's lymphoma, have been studied using lectin binding. The different histological subtypes of disease showed characteristic lectin-binding patterns which have enabled recognition of PBI in these diseases. The results suggest that even in cases where PBI is unexpected, abnormalities may often be found. No evidence was found for an increased PNA-reactivity or decreased sialic-acid levels in malignant cells. Further investigation of these abnormalities is continuing by correlating these findings with those using immunological surface markers.

**NEUTROPHIL FUNCTION IN NEUTROPENIC PATIENTS.** M. A. Cornbleet & G. A. Currie, *Divisions of Tumour Immunology and Medicine, Institute of Cancer Research, Sutton, Surrey*

Emission of light (chemiluminescence) during phagocytosis by neutrophils is correlated with increased glucose oxidation and the generation of bactericidal excited oxygen species (Allen *et al.*, 1972, *Biochem. Biophys. Res. Comm.*, **47,** 679). Addition of the nontoxic cyclic hydrazide, luminol increases the light yield by several orders of magnitude permitting a reduction in the required number of cells, so that the response of neutrophils from profoundly neutropenic patients can be assessed. Thirty-one patients with disseminated solid tumours who were neutropenic (total white count $< 10^9$/l) as a result of chemotherapy, were divided into 3 groups. Eight afebrile patients had chemiluminescence (CL) responses which did not differ from those of a control group of patients with normal white counts. Seven febrile patients with antibiotic-resistant, presumptively "non-bacterial" fevers, also had normal CL responses, but 16 patients with proven or presumed bacterial infection (antibiotic-responsive fevers) had significantly more active neutrophils. Two false-negative results occurred in this group, while one false-positive occurred in both the afebrile and the "non-bacterial" groups, the latter in a patient with a *Candida albicans* septicaemia. The overall misdiagnosis was 13%. Normal luminol-dependent CL activity after a trial of antibiotics in the neutropenic patient with a pyrexia of unknown origin, should suggest that potentially toxic treatment may safely be withdrawn, whereas increased activity in the presence of an antibiotic-resistant fever might suggest a fungal aetiology.

### $\alpha_1$ ANTITRYPSIN, A MARKER FOR HUMAN REACTIVE AND NEOPLASTIC MACROPHAGES.

D. B. Jones, P. Isaacson & K. M. Higginson, *University Department of Pathology, Southampton General Hospital*

The classification of malignant lymphoma has benefited greatly from the development of immunologically defined markers of cell lineage. Thus with regard to lymphocytic lymphoma, the malignant cell type can be related to normal counterparts present within the lymphocyte maturation sequence (Isaacson *et al.*, 1980, *J. Histochem. Cytochem.*, **28**, 761) .

Reliable markers for the identification of tumours of macrophage origin have, however, proved difficult to establish. Preliminary studies in this laboratory have suggested that $\alpha_1$ anti-trypsin ($\alpha_1$ anti-T) is a useful immunohistochemical marker of cells of the monocyte–macrophage series (Isaacson *et al.*, 1979, *Lancet*, ii, 964). We have extended the study of macrophage $\alpha_1$ anti-T to cultures of normal human monocytes and to the histiocytic cell line U937 (Sundstrom & Nilsson, 1976, *Int. J. Cancer*, **17**, 565).

Studies involving immunodiffusion and isotopic labelling have confirmed that macrophage $\alpha_1$ anti-T shows immunological identity with $\alpha_1$ anti-T present in serum, and isoelectric focusing suggests that this material is synthesized by cells of histiocytic lineage.

Positive staining for this glycoprotein is therefore a reliable marker for lymphoreticular neoplasms of histiocytic origin.

### CORRELATION OF 3 TUMOUR MARKERS (CALCITONIN, CEA AND $\beta$-hCG WITH RESPONSE TO THERAPY AND EXTENT OF DISEASE IN SMALL-CELL LUNG CANCER.

A. P. Sappino, M. L. Ellison, S. C. Carter & I. E. Smith, *The Royal Marsden Hospital and Ludwig Institute for Cancer Research (London Branch), Sutton, Surrey*

Plasma levels of 3 tumour markers (calcitonin, CEA and $\beta$-hCG were measured at presentation in 40 patients with small-cell lung carcinoma, to investigate whether there was any correlation with response to chemotherapy and extent of disease.

10 patients (25%) had raised calcitonin, 15 (38%) raised CEA and 7 (18%) raised $\beta$-hCG levels. 20 patients (50%) had no abnormal markers, 10 (25%) had one marker abnormal and 10 (25%) had 2 or 3 abnormal markers.

The response rate achieved with chemotherapy was related to the number of raised markers: of the 22 patients who responded, 19 had 0–1 marker raised, whereas only 2 had 2–3 markers raised. Strikingly only 2 of the 10 patients with high calcitonin responded to therapy.

The extent of disease at presentation correlated with the number of raised markers: 15/30 patients with 0–1 marker raised had extensive disease, compared with 8/10 with 2–3 markers raised. In particular 12/15 with high CEA had extensive disease.

These results indicate that certain tumour markers or combinations of markers may help to predict both response to therapy and extent of disease in patients with small-cell lung cancer, with important prognostic implications.

**THE "HOT SPLEEN" PHENOMENON IN ADVANCED MALIGNANT MELANOMA.** J. WAGSTAFF, K. PHADKE, N. ADAMS*, N. THATCHER & D. CROWTHER, *CRC Dept of Medical Oncology, Christie Hospital, *Dept of Diagnostic Radiology, University Hospital of South Manchester*

Technetium-99M Sulphur Colloid liver scans were performed in a series of patients with advanced malignant melanoma (MM), (Stages II and III) prior to treatment with immunotherapy or chemotherapy. Patients with metastatic and non-metastatic liver disease, anaemia, infection and diabetes mellitus were excluded from the analysis. Review of 124 cases showed that 36% with Stage II disease and 43·6% with Stage III displayed a "hot spleen", with greater density of counts over the spleen than the liver. The feature increased in frequency with stage of disease, but was not associated with a shorter disease-free interval or worse survival. Others (Sober *et al.*, 1979, *J. Nucl. Med.*, **20**, 1232) have shown a higher relapse rate in Stage I patients with "hot spleens". The phenomenon was commoner in females than males and there was a significant association with a high serum IgM level ($P = 0{\cdot}02$).

The feature seems to be due to augmented activity of splenic macrophages due to the presence of tumour, possibly due to stimulation by tumour-associated antigen, immune complexes or both. It has resolved after surgical excision of localized tumour (Klingensmith, 1974, *J. Nucl. Med.*, **15**, 1203) and its persistence after primary excision of tumour in Stage I patients may reflect the presence of residual disease. As disease advances, macrophage function becomes impaired. This may explain why this feature fails to be of prognostic value in Stages II and III. Oestrogens have been shown to stimulate phagocytic activity in mice, and this may account for the increased incidence in female patients.

### POSTERS

**9,10-DIMETHYL - 1,2 - BENZANTHRACENE (DMBA) AND 12-O-TETRADECANOYL PHORBOL 13-ACETATE (TPA) INDUCED CHROMOSOME CHANGES IN PRIMARY CULTURES OF MOUSE AND HUMAN EPIDERMAL CELLS.** J. BROADHEAD & A. R. KINSELLA, *Paterson Laboratories, Christie Hospital and Holt Radium Institute, Manchester*

The classical system for the study of 2-stage carcinogenesis *in vivo* has been mouse skin. Primary cultures of epidermal cells isolated from neonatal BALB/c or SENCAR mouse skin are being studied *in vitro*. Results from this system are compared with those obtained from human keratinocytes *in vitro*.

Chromosome changes have often been implicated in the process of neoplastic transformation. *In vitro* epithelial-cell systems allow this process to be followed through the stages of initiation and promotion, eventually giving rise to fully transformed cells. It is therefore possible to study chromosome changes associated with the progression towards malignancy. Primary mouse and human epidermal-cell cultures were treated with DMBA (1–200 ng/ml) or TPA (1–1000 ng/ml) and fixed *in situ* for chromosome analysis at later stages. Within one day of treatment with a single dose of DMBA, mouse epidermal-cell chromosomes show altered configurations such as metacentric chromosomes and chromatid fragments. Five days after treatment triradial and quadriradial formations are seen. At similar concentrations of DMBA, human epidermal-cell chromosomes show no such aberrations, but there appears to be an increase in the level of aneuploidy above that in control cultures. Addition of a single dose of TPA to any of the epidermal-cell cultures has no effect on chromosome configuration, aneuploidy or polyploidy. Autoradiographic studies are being conducted to provide data concerning the growth characteristics of the cultures at different stages following treatment.

**VOLUMETRIC INCREASES IN BLOOD VESSELS DURING EPIDERMAL CARCINOGENESIS.** B. AL-AZZAWI & F. H. WHITE, *Department of Human Biology and Anatomy, University of Sheffield.*

The aim of the present investigation is to quantify changes in blood-vessel volume in skin after the topical application of 7,12 dimethyl-benz(α)anthracene (DMBA). The dorsal skin of 20 male Syrian hamsters was shaved and they were assigned to 4 groups of 5 animals. The first group received no DMBA applications and served as a control, whilst the others were treated 3 × /week with a 0·5% solution of the chemical carcinogen DMBA. Five animals were sacrificed after 9, 12 and 16 weeks of application. Tissue samples were obtained and routinely processed for light microscopy. Representative samples were analysed using a MOP AMO3 (Kontron) semi-automatic image analyser. The areas of blood vessels in both dermis and hypodermis were estimated for the normal and for each experimental group, as were the areas of dermis and hypodermis. From this data volume densities could be calculated. The blood-vessel volume densities for both dermis and hypodermis of the normal control group were 0·0009 and 0·0040 respectively. The results show progressive increases in volume densities in both dermis and hypodermis after both 9 and 12 weeks application of DMBA, and there was a pronounced increase in vascularity in animals after 16 weeks treatment. Our results support the concept that DMBA application increases the volume of blood vessels in the adjacent connective tissue. However, as yet we do not know whether this is due to an increased production (angiogenesis) or is simply a reflection of the inflammatory response which invariably accompanies carcinogenesis.

### STEREOLOGICAL INVESTIGATIONS OF DESMOSOME FREQUENCY DURING ORAL CARCINOGENESIS.

F. H. WHITE & K. GOHARI, *Department of Oral Pathology, University of Sheffield*

Desmosomes are membrane specializations which are responsible for intercellular attachment. There are many subjective reports describing alterations in desmosomes in malignant lesions. The development of stereological methods (Weibel, 1969, *Int. Rev. Cytol.*, **26,** 235) now enables morphology to be placed on a quantitative basis. Thus the aim of this investigation was to quantify desmosome frequency using stereological methods during sequential DMBA carcinogenesis in hamster cheek pouch. After DMBA treatment, pouch samples were removed and epithelial lesions were classified into hyperplasia, dysplasia and carcinoma groups. Untreated pouch epithelium served as a control group. Representative samples from 5 animals in each group were used to obtain electron micrographs of defined basal, spinous and granular cells. Using a test lattice comprising parallel lines, intersections of the lattice lines with desmosomes and plasma membrane were counted. Using these data the number of desmosomes per unit area of plasma membrane ($N_S$) was estimated from the formula $N_S = N|A|\bar{\Delta}$ where N is the number of desmosomes, A the surface area on which they are present and $\bar{\Delta}$ the mean desmosome diameter. $N_S$ values were obtained for basal, spinous and granular layers of each group. The results indicate that during chemical carcinogenesis the parameter $N_S$ decreases by more than 50% in basal and spinous cells. This decrease in desmosomal frequency may be a requirement for carcinoma cell invasion and metastasis.

### ALTERATIONS IN THE FREQUENCY OF GOLGI COMPLEXES DURING ORAL CARCINOGENESIS IN THE HAMSTER CHEEK POUCH.

F. H. WHITE & K. GOHARI, *Departments of Oral Pathology and Human Biology and Anatomy, University of Sheffield*

The metabolic characteristics of tumours are altered from their tissue of origin and it is possible that such alterations are accompanied by changes in the morphology of intercellular organelles. The present study evaluates the possibility that the Golgi complex, which is actively involved in the synthesis of cell-surface carbohydrates, is altered in frequency during the process of chemical carcinogenesis. This work is part of a larger morphometric investigation to determine whether any specific ultrastructural morphological alterations exist which might be of value in the detection of oral precancer. After application of 0·5% DMBA to cheek pouches of Syrian hamsters, tissue samples were obtained, processed for electron microscopy and on the basis of light-microscopical evaluation of Araldite-embedded material were assigned to hyperplasia, dysplasia and carcinoma stages by strict criteria. Untreated epithelium served as a control. Following a stratified sampling procedure, micrographs from defined basal, spinous and granular layers were obtained from normal and pathological stages, and using point counting methods, the number of Golgi complexes per unit volume of cytoplasm ($N_{VGOL}$) was determined for each cellular lyaer at each stage. Values for $N_{VGOL}$ were similar in basal cells in normal and all pathological stages. However in spinous and granular layers, $N_{VGOL}$ decreased progressively between normal and carcinoma stages. These results may reflect the failure of normal differentiation in carcinogen-treated epithelium, and further morphometric information on a variety of non-malignant epithelial conditions is required before the value of this parameter as a diagnostic indicator can be determined.

**QUANTITATIVE ALTERATIONS IN NUCLEAR PORES DURING EXPERIMENTAL ORAL CARCINOGENESIS.** R. M. Codd, F. H. White & K. Gohari, *Departments of Human Biology and Anatomy and Oral Pathology, University of Sheffield*

Nuclear pores are small channels in the nuclear envelope which permit the passage of large molecules between the nucleoplasm and the cytoplasm. We have previously described methods for quantifying these structures in normal hamster cheek-pouch epithelium, and the present study was designed to establish whether there are any quantitative changes in the relative surface areas of nuclear pores in premalignant epithelial tissues. Following application of DMBA to hamster cheek-pouches, tissue was removed, processed for electron microscopy and semithin sections examined in order to assign lesions to hyperplasia and dysplasia stages. Following a strict sampling procedure, micrographs were obtained from basal, spinous and granular-layer nuclei for each pathological stage. Stereological intersection counting was performed to estimate the relative surface area of nuclear pores present on the nuclear membrane ($Ss_{np,nm}$) for each cell layer at each pathological stage. Initial results suggest that the $Ss_{np,nm}$ is lower in both DMBA-treated groups when compared to the untreated controls. The observed decrease may reflect alterations in cellular differentiation during pre-cancer, such as decreased frequency of tonofibrils and keratohyaline granules. We are currently using similar methods to quantify relative surface areas in carcinomas, and are expanding the study to include parameters such as number of pores per unit surface and mean pore diameter, in order to characterize these morphological changes more precisely.

**LOSS OF BODY WEIGHT MODIFIES COLONIC CARCINOGENESIS AFTER SUBTOTAL JEJUNO-ILEAL BYPASS IN RATS.** J. B. Bristol & R. C. N. Williamson, *University Department of Surgery, Bristol Royal Infirmary*

Although small-bowel resection promotes experimental intestinal carcinogenesis, subtotal bypass may have a protective effect (Williamson *et al.*, 1980, *Cancer Res.*, **40**, 538). Since changes in body weight might explain the discrepancy, colorectal carcinogenesis was studied after different types of enteric bypass. Male Sprague-Dawley rats weighing $117 \pm 0.8$ g (s.e.) were given 6 s.c. weekly injections of azoxymethane (15 mg/kg). One week later each rat was subjected to 85–90% jejuno-ileal bypass (3 groups) or sham bypass (SB), comprising jejunal transection, ileotomy, and resuture. Bypass was (1) end-to-side; (2) end-to-end with a Thiry-Vella fistula (TVF) or (3) end-to-end with drainage of the bypassed loop into the descending colon (LDC). Twelve weeks after the initial operation, the self-emptying blind loop was resected in half the rats with end-to-side bypass. At 30 weeks the 61 surviving rats were killed. SB rats weighed $586 \pm 23$ g and had $3.9 \pm 1.0$ colorectal tumours per rat. End-to-side bypass more than doubled the number of colorectal tumours to $10.3 \pm 1.4$ ($P < 0.01$), despite reducing body weight to 73% that of SB rats ($P < 0.001$). Bypass with TVF or LDC caused even greater weight loss (55% of SB). Colorectal tumour yields of $5.3 \pm 0.8$ (TVF), and $5.8 \pm 1.2$ (LDC) were not significantly different from SB rats. In rats with end-to-side bypass, resection of the blind loop did not alter the final body weight; the number of tumours ($7.0 \pm 2.7$) was again greater than in SB rats ($P < 0.05$).

Jejuno-ileal bypass enhances colorectal neoplasia. This effect is abolished by profound reduction of body weight, but not by mid-term resection of the bypassed loop.

### LABORATORY USAGE OF SOME SUSPECT CARCINOGENS.

F. DEWHURST, *School of Life Sciences, Leicester Polytechnic*

Recent studies by Olin *et al.* (1980, *Env. Res.*, **22,** 154) on Swedish chemists and by Searle *et al.* on British chemists have produced some evidence of a greater than expected incidence of tumours. There is little or no information on exposure of chemists to suspected carcinogens.

A questionnaire safety survey of British laboratories was carried out through "Laboratory News" in 1973. A total of 1178 replies was received covering all types of laboratory. Questions were asked concerning the usage of certain types of suspect chemical carcinogen.

Less than 1% of replies stated sulphur mustards were used, 1–5% of replies stated nitrogen mustards, ethylene imines, beta propriolactone thioacetamide and nitrosamines were used, whilst 5–10% stated that asbestos (finely powdered), beryllium, and urethane were used. Between 10 and 20% of replies reported epoxides, thiourea, diazomethane, acetamide, hydrazine, nickel powder, cadmium and arsenic and their compounds. Chromium and its compounds were used by 34% of those replying, whilst the use of lead and its compounds was reported by 40%. The most commonly reported carcinogens were the solvents chloroform (74% of replies), carbon tetrachloride (66%) and benzene (48%). Weekly or more frequent usage of benzene was reported in 10% of replies, of carbon tetrachloride in 17% and of chloroform in 24%.

Evidence in the survey showed serious contamination of the laboratory atmosphere with solvent vapour in about a third of all cases. In about 15%, formaldehyde levels high enough to cause eye irritation were noted.

### CHLORAMBUCIL-INDUCED CHANGES IN CHROMATIN METHYLATION.

M. L. RAMIREZ, R. SHEPHERD, S. PINSKY, K. MCGHEE & K. R. HARRAP, *Department of Biochemical Pharmacology, Institute of Cancer Research, Sutton, Surrey*

Post-synthetic methylation of nucleic acids and nuclear proteins is a putative regulatory event in the function and structure of chromatin. Changes in methylation may also be determinants of alkylating-agent toxicity, in the same way as has been shown for chromatin protein phosphorylation (*Cancer Res.*, 1979, **39,** 4256). We studied the effects of chlorambucil treatment on DNA and nuclear protein methylation in sensitive (S) and resistant (R) Walker 256 carcinosarcoma cells *in vitro* (2h treatment) and *in vivo* (24h treatment). Incorporation of [$^3$H-methyl]-methionine (Met) into whole cells or [$^3$H-methyl] S-adenosylmethionine (SAM) into isolated nuclei was measured. At an $ID_{50}$ dose, DNA, histone and non-histone methylation was stimulated in S cells but not in R cells (Met assay). An $ID_{90}$ dose inhibited methylation in both S and R cells. Similar results were obtained using isolated nuclei, where only histone methylation was detected (non-histone methyl-transferases are cytoplasmic enzymes). DNA methylation at the 5 position of cytosine was inhibited at $ID_{90}$ doses. The transcriptional inducer, sodium butyrate, at an $ID_{90}$ dose *in vitro* (5 mmol/l for 48 h) inhibited methylation by 50–70%, whilst an $ID_{10}$ dose (2mmol/l, 24 h) caused less inhibition. Pretreatment with butyrate inhibited chlorambucil-mediated enhancement of methylation. These data indicate that changes in methylation of chromatin proteins and DNA paralleled alkylating-agent toxicity. Nuclear protein methylation has been associated with chromatin condensation, and its inhibition at $ID_{90}$ doses correlated with the loss of heterochromatin observed in these cells.

### PHARMACOKINETIC INVESTIGATIONS OF 5-FLUOROURACIL IN BREAST CANCER PATIENTS.

B. J. MCDERMOTT, H. VAN DEN BERG* & R. F. MURPHY, *Departments of Biochemistry and *Therapeutics and Pharmacology, Queen's University, Belfast*

Studies on the urinary excretion kinetics of 5-fluorouracil (FU) were performed using an ion-specific electrode (ISE) technique. FU and metabolites contain organically bound fluorine, which is degraded by oxygen-flask combustion to fluoride ion for detection by the electrode. Prior to estimation, FU and metabolic products may be separated by gel filtration on Bio-Gel P-2. Five patients receiving i.v. bolus injections of FU (300–500 mg) on each day of a 5-day combination chemotherapeutic regime, were investigated. Analysis of 24h specimens showed decreasing excretion of total organic fluorine with successive doses, indicating accumulation of the drug. Two patients, however, eliminated the greatest amounts of FU and metabolites into the urine on Day 3 of therapy, when the drug was administered alone. Sampling of 3 patients at timed intervals on Day 3 allowed determination of their elimination kinetics for FU ($t\frac{1}{2}$ = 37·4, 43·9 and 18·8 h). These values are inconsistent with results from our previous studies of plasma pharmacokinetics. Using a gas-liquid chromatographic-electron capture (GLC-EC) method, 2 phases of elimination were described with $t\frac{1}{2}^{\alpha} = 12{\cdot}1 \pm 1{\cdot}0$ (s.e.) min and $t\frac{1}{2}^{\beta} = 123{\cdot}5 \pm 22{\cdot}2$ min. Assessment of the terminal portion of the decay function has important therapeutic considerations as it probably reflects clearance of the active form of the drug from tissues. Due to limitations in sensitivity of the GLC-EC technique (20 ng/ml), interpretation of plasma profiles is difficult, and it is suggested that the kinetic parameters estimated from urinary-excretion data are more accurate. Using a combination of the ISE and GLC-EC methods, the excretion patterns of the parent drug and individual metabolites may be determined. The characterization of metabolites separated by gel filtration has yet to be completed.

### FOLATE CATABOLISM: ALTERATIONS IN MALIGNANT DISEASE.

A. M. SALEH, A. E. PHEASANT, J. A. BLAIR, R. N. ALLAN & J. WALTERS, *Department of Chemistry, University of Aston in Birmingham*

In rat, guinea-pig and man, folates are catabolized to p-acetamidobenzoyl L-glutamate (p-AcBG), p-acetamidobenzoate (p-AcBA) and various pteridine fragments (Choolun *et al.*, 1980, *Biochem. Soc. Trans.*, **8,** 568; Saleh *et al.*, 1980, *Biochem. Soc. Trans.*, **8,** 566). Folate catabolism is decreased in tumour-bearing rats (Barford & Blair, 1978, *Br. J. Cancer*, **38,** 122). In this study, consenting hospital in-patients received an oral dose of [2–$^{14}$C]+[3′,5′,7,9-$^{3}$H] folic acid (5 mg folic acid) and urine was collected for 24 h. Ten patients with malignant disease were compared with a control group (5) suffering from other disorders. Cancer patients excreted significantly ($P < 0{\cdot}001$) less radioactivity in urine; 14·7% $^{3}$H, 12·4% $^{14}$C of the dose compared to 32·0% $^{3}$H, 26·2% $^{14}$C for the controls. Where determined, faecal radioactivity was similar in the 2 groups (0·2–10·8% $^{3}$H, 0·3–28% $^{14}$C). Total urinary scission-product excretion (p-AcBG+pAcBA) was decreased in cancer patients; 2·0% of the dose compared to 4·3% by the controls. p-AcBG is derived from tissue folate polyglutamates. Based on the radioactivity retained in the tissues, the difference in catabolism by the 2 groups is even more marked: 1·0% of the retained $^{3}$H being excreted as p-AcBG by the cancer patients com: pared to 2·4% by the controls. The mechanism of folate breakdown may involve the oxidation of labile reduced folate. Thus the decrease in catabolism associated with malignancy may be explained by the altered redox state and anoxia of tumour cells.

### INCREASED THERAPEUTIC ACTIVITY OF THIOTEPA IN EXPERIMENTAL MOUSE COLON TUMOURS FOLLOWING NANDROLONE DECANOATE PRE-TREATMENT.

J. A. DOUBLE, M. C. BIBBY & M. A. MUGHAL, *Clinical Oncology Unit, School of Medical Sciences, University of Bradford*

We have previously reported (Double & Bibby, 1981, *Br. J. Cancer*, **42,** 171; Bibby *et al.*, *Br. J. Pharmacol.*, in press) that pretreatment with nandrolone decanoate (N.D.) raises the $LD_{50}$ of CCNU and 5FU in NMRI mice without altering the anti-tumour activity against transplantable mouse colon tumours. The present study reports an extension of this work, using 2 tumour lines with differing histology and growth characteristics, both of which are sensitive to Thiotepa. MAC 26 is a slow-growing well differentiated adenocarcinoma, whereas MAC 13 is less well differentiated and grows more rapidly. The differing growth rates of these tumour lines necessitated the use of 2 different treatment protocols. The growth of MAC 26 can be easily followed by serial caliper measurements. Chemotherapy begins ~14 days after transplantation, when tumours have a mean volume of ~250 mm$^{3}$. The rapid growth of MAC 13 makes this method impractical. Chemotherapy in this line is administered 2 days after implantation, and the effects are determined 14 days later by comparison of tumour weights. Peripheral WBC counts in normal mice and mice receiving ND and thiotepa were measured with a Coulter S plus. In both systems ND had no significant effect on tumour growth or on the anti-tumour action of thiotepa, but reduced toxicity, leading to an increase in therapeutic index. Although this reduction in toxicity was less than we have previously reported for CCNU and 5FU, the dose-response curve for thiotepa is much steeper than for these other agents. Peripheral WBC counts indicate that there is a significant protection of the haemopoietic system, which in a corresponding clinical situation would be of importance to patients undergoing cytotoxic therapy with thiotepa.

### FACTORS INFLUENCING KILLING OF HUMAN TUMOUR CELLS BY MELPHALAN *IN VITRO*.

V. D. Courtenay & Judith Mills, *Radiotherapy Research Unit, Institute of Cancer Research, Sutton, Surrey*

*In vitro* sensitivity testing of chemotherapeutic drugs against tumour cells from biopsy specimens is being widely investigated as a possible means of predicting the clinical sensitivity of tumours in individual patients. It is therefore important to determine *in vitro* test conditions such that cell survival at a given drug dose may be directly related to *in vivo* response.

In these studies using cell suspensions prepared from a human pancreatic adenocarcinoma xenograft (HX32) and treated *in vitro* with melphalan, survival was assayed by an agar colony technique. The effect of cell disaggregation technique, cell sedimentation, cell concentration, serum concentration and pH during treatment was examined. A dose modification factor greater than 3 was obtained, depending on the conditions of treatment and recovery. Tumour cell survival was found to depend on serum concentration during treatment and on the type of serum used both during and after exposure to drug. Incubating disaggregated cells at 37°C for 1 h before treatment also affected survival. The shape of survival curves was dependent on whether cells were allowed to settle out during treatment or maintained in suspension.

These studies indicate some possible sources of error in using "*in vitro*" tests to rank "*in vivo*" effectiveness of drugs with different pharmacological properties and modes of action·

### THE EFFECTS OF CYCLOPHOSPHAMIDE AND ITS DERIVATIVES ON ADENYLATE CYCLASE ACTIVITY AND PROTEIN SYNTHESIS.

L. A. Fitton, G. J. Hunter & B. E. P. Swoboda, *Department of Chemistry and Molecular Sciences, University of Warwick, Coventry*

Nitrogen mustard chemotherapeutic agents are considered to obtain their effect by cross-linking DNA, though other cellular targets have been implicated (*Eur. J. Cancer*, 1977, **13**, 1363). We have examined the effects of cyclophosphamide (CP) and its metabolites on the enzymes of cyclic-nucleotide metabolism and protein synthesis.

CP, 4-keto-CP (KP) and phosphoramide mustard (PM) ($\leq 15$ mmol/l) were found to have no effect on basal or stimulated hepatic adenylate cyclase activity. 4-hydroperoxy-CP (HP, 5 mmol/l), was found to inhibit enzyme activity (50%) and completely abolished the effect of glucagon but only partially abolished the stimulatory effect of fluoride. Whereas CP and KP are "inactive", PM is a potent alkylating agent and is considered as the "ultimate" alkylating metabolite of CP. These results may be explained by the unique reactivity of HP (*Cancer Treat. Rep.*, 1976, **60**, 355). Similar specificity has been observed in the sensitivity of cAMP phosphodiesterase and protein kinase for HP (*Biochem. Pharmacol.*, 1977, **26**, 1469).

In a separate series of experiments, we have found that protein synthesis is inhibited by low concentrations of CP metabolites. HP (10 mmol/l), inhibited protein synthesis in rabbit reticulocyte lysate by 50%. PM and $HN_2$ showed only a slight effect at this concentration (up to 10% inhibition).

### DIFFERENTIAL RESPONSES TO X-IRRADIATION, 5-FLUOROURACIL OR METHOTREXATE IN A RANGE OF HUMAN AND MURINE TUMOUR CELLS *IN VITRO*.

A. S. Bellamy, R. D. H. Whelan & B. T. Hill, *Laboratory of Cellular Chemotherapy, Imperial Cancer Research Fund, London*

Clinical studies have indicated that prior radiation reduces response to subsequent chemotherapy. Studies were undertaken to determine whether there is any correlation between *in vitro* sensitivities to X-irradiation and response to methotrexate (MTX) or 5-fluorouracil (FU) in a range of mammalian cell lines. Sensitivity was assessed by colony-forming assays, in soft agar or on plastic.

Drug-resistant lines were derived *in vitro* from murine L5178Y lymphoma cells by continuous exposure to either MTX, FU or both. A radiation-treated line was obtained by exposure to 10 fractions of ~2 Gy. The results showed a range of sensitivities to radiation, with $D_0$ values of 32–58 rads, which appeared inversely correlated with response to MTX. No such relationship was seen in the case of FU.

Four human tumour-cell lines showing a range of sensitivities to MTX were then examined. SCC-T/G and Hep2 were derived from primary squamous-cell carcinomas of the tongue and larynx respectively, and LAN-1 and CHP-100 derived from neuroblastomas. These human lines also showed a range of $D_0$ values for radiation of 0·6–1·8 rads. No correlations involving FU were noted, as for the murine cells. In addition, no relationship was seen between the responses to MTX and FU in any of the cell lines tested. Furthermore, no clear relationship between sensitivity to MTX and resistance to radiation was shown. This aspect is being investigated further, using specific drug-resistant and radiation-resistant human tumour-cell lines.

### A CYTOFLUORIMETRIC STUDY OF THE COLLATERAL SENSITIVITY OF A METHOTREXATE-RESISTANT CELL AGAINST THE VINCA ALKALOIDS.

A. McGown, D. G. Poppitt & B. W. Fox, *Paterson Laboratories, Christie Hospital and Holt Radium Institute, Manchester*

L1210 cells sensitive and resistant to MTX exhibit a collateral sensitivity towards the Vinca alkaloids. The cell cycle perturbations induced by the Vinca alkaloids were examined by flow cytometry and it was shown that both the sensitive and resistant cell lines became blocked in G2, but that the resistant line remained blocked at much lower levels of vinca alkaloid than the sensitive. It is concluded that drug resistance due to gene reduplication may be accompanied by an increased difficulty in undertaking mitosis, and that the Vinca alkaloids may exaggerate this effect.

### CARBOHYDRATE-LINKED MELAMINE DERIVATIVES.

S. P. Langdon, R. J. Simmonds & M. F. G. Stevens, *C.R.C. Experimental Chemotherapy Group, Department of Pharmacy, University of Aston in Birmingham, and G. Atassi, Institut Jules Bordet, 1000 Brussels*

Efforts to improve the water solubility of hexamethylmelamine (HMM, 1) by molecular modification generally produce dyschemotherapeutic effects (Cumber & Ross, 1977, *Chem. Biol. Interactions*, **17**, 349). We have developed a synthetic method to conjoin sugars and cytotoxic melamine fragments: for example interaction of the quaternary salt (2) with glucosamine or D-glucose yields the "sweet melamines" (3) and (4) respectively.

(1) R = $NMe_2$
(2) R = $NMe_3Cl$

(3)

(4)

Despite their improved solubilities these new agents have no inhibitory effects on the mouse M-5076 ovarian carcinoma, whereas HMM and related cogeners are active (Table).

| | Compound | TVI (%)* | Optimal dose† (mg/kg) |
|---|---|---|---|
| (1) | R = $NMe_2$ | 70 | 150 |
| (1) | R = NHMe | 65 | 150 |
| (1) | R = N(Me)$CH_2OH$ | 67 | 160 |
| (3) | | inactive | 200‡ |
| (4) | | inactive | 300‡ |

* Tumour volume inhibition on Day 24.

† Drugs injected on alternate days over 21-day period.

‡ Maximum dose tested.

**SERUM 5α-ANDROSTANE-3α, 17β-DIOL IN PATIENTS WITH PROSTATIC TUMOURS.** R. GHANADIAN, C. M. PUAH & G. WILLIAMS, *Prostate Research Laboratory, Royal Postgraduate Medical School, Ducane Road, London, and Institute of Urology, University of London*

Concentrations of serum 5α-androstane-3α, 17β-diol (diol) were estimated in patients with benign and malignant tumours of the prostate. This androgen which is a metabolite of 5α-dihydrotestosterone (DHT) has been implicated in the growth and functional activities of the prostate. Thirty-two patients with proven prostatic cancer (Ca) aged 51–85 years, 32 patients with benign prostatic hypertrophy (BPH) aged 54-84 years and 24 normal subjects aged 51–80 years were investigated. The mean $\pm$ s.e. for serum concentrations of diol for BPH, Ca and normal subjects were $813 \pm 43$, $524 \pm 35$ and $685 \pm 28$ pM respectively. Statistical analyses of the results showed that the level of this steroid in BPH patients was significantly higher than either the normal subjects ($P < 0{\cdot}05$) or Ca patients ($P < 0{\cdot}001$). Furthermore, the level of this androgen in Ca patients was significantly *lower* than that of the normal group ($P < 0{\cdot}005$). The higher level of diol in BPH patients than in the aged-matched control group corresponds to that reported for 5α-dihydrotestosterone, which is an immediate precursor of this stero id (Ghanadian *et al.* 1977, *Br. J. Urol.*, **49**, 541). Although a significant difference has been found between the levels of this androgen in patients with GPH and Ca, the scattered values within each group does not provide an index to differentiate the 2 types of tumours.

**CORRELATIVE STUDIES BETWEEN ENDOGENOUS STEROIDS AND STROMAL-EPITHELIAL COMPOSITION IN HUMAN BENIGN HYPERTROPHIED PROSTATE.** C. M. PUAH & R. GHANADIAN, *Prostate Research Laboratory, Royal Postgraduate Medical School, Ducane Road, W.12 OHS, and Institute of Urology, London WC2.*

The relationship between endogenous steroids and the compositions of stromal and epithelial cells in prostatic tissues were investigated in order to evaluate differential localization of steroids within cell types. Tissues were obtained from 14 patients aged 54–79 years with benign prostatic hypertrophy (BPH). Prostatic tissues were removed by retropubic prostatectomy. The clinical diagnosis of the disease was confirmed by histological examination of the operative specimen. Testosterone (T), 5α-dihydrotestosterone (DHT), 5α-androstane-3α, 17β-diol (diol) and oestradiol-17β, ($E_2$) were measured by radioimmunoassay developed in our laboratory. Representative sections of each tissue were stained with H & E. Morphometric analyses were carried out by point counting with an aid of a superimposed ocular grid (100 Sq.). The results showed that $E_2$ is predominantly localized in the stroma ($r = 0{\cdot}63$, $P < 0{\cdot}005$), whereas diol is mainly associated with the glandular fraction, epithelial + acinar ($r = 0{\cdot}53$, $P < 0{\cdot}05$). Further associations were also found between the ratios diol/$E_2$ with both epithelial ($r = 0{\cdot}57$, $P < 0{\cdot}05$) and glandular fractions ($r = 0{\cdot}70$, $P < 0{\cdot}01$). Neither T nor DHT revealed any preferential localization with epithelial or stromal elements. It would appear from this study that an androgen–oestrogen balance may be involved in the changes of stromal-epithelial composition and hence in the secondary growth of the prostate.

**RECTAL BIOPSY IN THE STAGING OF NON-HODGKIN'S LYMPHOMAS.** R. C. F. LEONARD & C. MCCORMICK, *Oxford Lymphoma Group, Churchill Hospital, Oxford*

In a study of non-Hodgkin's lymphoma, patients were routinely subjected to rectal biopsy as a part of the staging procedure at presentation. In every case the mucosa was macroscopically normal, but histological examination revealed evidence of lymphoma deposits in 16/80 patients biopsied. The site of disease was lamina propria, often with extension through the muscularis mucosa. Analysis of patients by (Kiel) histopathology group, site of clinical disease and stage showed that microscopic invasion of the bowel mucosa occurred in most sub-groups except localized high-grade lymphomas. Thus in clinical Stage III or IV low-grade lymphomas the rate of detection was much higher, particularly in the centroblastic–centrocytic lymphomas and lymphoplasmacytoid lymphomas. These findings give further support to the concept of disease being widespread at presentation among low-grade non-Hodgkin's lymphomas.

**THE PRESENCE OF RETINOIC ACID RECEPTOR IN HUMAN BLADDER TUMOURS.** S. L. Fagg, A. Hughes, P. Dawson-Edwards & M. A. Hughes, *Department of Surgical Immunology, Department of Medicine and Department of Urology, Queen Elizabeth Hospital, Birmingham*

Specific intracellular receptors for retinoic acid (RAR), analogous to receptors for steroid hormones, are found in a variety of malignant tissues including human lung, breast, cervical, endometrial and ovarian tumours (Ong *et al.*, 1975, *Science*, **190**, 60; Kung *et al.*, 1980, *Cancer Res.*, **40**, 4265. Palan *et al.*, 1980, *Cancer Rcs.*, **40**, 4221). There is no previous report of RAR in bladder tumours. In this study RAR was measured in human bladder tumour specimens obtained by transurethral resection. Tumour specimens were frozen in liquid $N_2$, and pulverized with a Mikro-Dismembrator. The powder was suspended in 4 vols (w/v) of cold buffer (10mM Tris, 1·5mM EDTA, 10mM DTT, 1·4M glycerol, pH 7·4) and cytosol prepared by high-speed centrifugation (240,000 $g \times 1\frac{1}{2}$ h). Specific binding was assessed using an agarose-gel electrophoresis system (Huber *et al.*, 1978, *J. Natl Cancer Inst.*, **61**, 1375), following incubation with labelled RA (40 Ci/mmol—a gift from Roche, Nutley, N.J.) together with the appropriate controls containing an excess of unlabelled RA. Retinol did not compete in this system nor was there any specific binding of RA in serum. RAR was detectable in the cytosols of 6 specimens (ranging from 0·15–1·13 pmol RA/mg cytosol protein). There was no detectable RAR in 3 specimens. These results suggest that RA and its synthetic analogues may be useful in the treatment of some bladder tumours. This study is continuing, and will be combined with a clinical trial using retinoids in the treatment of patients with bladder tumours.

**REDUCED GVHR in $F_1$ HYBRID MICE, INJECTED WITH PARENTAL LYMPHOID CELLS, IN THE PRESENCE OF AN $F_1$ TUMOUR.** T. Whitmarsh-Everiss & M. O. Symes, *Department of Surgery, University of Bristol*

A graft *vs* host reaction (GVHR) was induced in (A × CBA(T6)) $F_1$ mice by injection of A-strain spleen cells. The magnitude of the resulting GVHR was measured by an increase in spleen weight. The presence of an $F_1$ tumour less the GVHR in 5 experiments. At the same time tumour size was reduced in animals undergoing GVHR than in animals receiving tumour alone. These effects were not seen in $F_1$ hybrid animals bearing an A-strain tumour. Excision of an $F_1$ tumour from $F_1$ hosts, which then received A-strain spleen cells, led to a greater GVHR than in animals from which the tumour was not excised. Thus the reaction of A spleen cells against an $F_1$ tumour may protect an $F_1$ host from GVHR by preoccupation of the A cells.

Spleen cells from $F_1$ mice were transferred to 3–8-day $F_1$ litter mates, at various intervals after induction of GVHR by injection of A-strain spleen cells. Other $F_1$ mice from the same litters received cells from $F_1$ mice undergoing GVHR in the presence of an $F_1$ tumour. A spleen cells from the tumour-bearing $F_1$ hybrids induced less GVHR in $F_1$ litter mates, than parental cells from non-tumour-bearing hybrids. Thus the presence of a tumour also depressed injected parental cell reactivity.

**APPLICATION OF A MONOCLONAL ANTIBODY TO RAT HEPATOCYTES IN THE STUDY OF RAT LIVER CARCINOGENESIS.** C. Holmes, B. Gunn, E. B. Austin and M. J. Embleton, *Cancer Research Campaign Laboratories, University of Nottingham*

BALB/c mice were immunized with rat hepatocytes, isolated from regenerating rat liver by perfusion and disaggregation with collagenase. Spleen cells from an immunized mouse were fused with P3NS1 mouse myeloma cells to produce hybridomas secreting antibodies to rat hepatocytes. After screening against cells derived from different rat tissues, one hybridoma was selected which produced antibody reacting preferentially with cells from regenerating and normal syngeneic liver, allogeneic liver and foetal liver. This antibody showed a borderline reactivity with normal rat kidney, but was completely negative for a range of other rat tissues and guinea-pig hepatocytes. The hybridoma was cloned, and is now producing a monoclonal antibody with identical specificity.

Preliminary tests have been carried out to determine whether the expression of the antigen detected by this antibody is altered during liver carcinogenesis, and the results indicate that it is reduced or absent in some rat hepatomas. These studies are being extended to establish the stage of carcinogenesis at which this change in antigen expression occurs.

### A DIFFERENCE IN THE GROWTH RESPONSE OF CAPILLARY AND AORTIC ENDOTHELIAL CELLS TO TUMOUR ANGIOGENESIS FACTOR (TAF).

A. L. BRIERLEY, *Christie Hospital, Manchester*

Capillary-derived, and aorta-derived endothelial cells were grown on plastic dishes and native collagen gels. Addition of TAF to these cultures and examination of subsequent cell numbers revealed: (a) Capillary cells growing on native collagen gels were stimulated to proliferate by addition of TAF; (b) No stimulation was observed for either aorta-derived cells growing on plastic or native collagen gels, or for capillary cells on a plastic substrate.

This data suggests a functional difference between large and small-vessel endothelial cells, a difference which has wide implications when undertaking studies on diseases involving endothelial-cell disorders.

### DETECTION OF HUMAN OSTEOGENIC-SARCOMA CELL-SURFACE ANTIGENS USING RADIOLABELLED ANTI-TUMOUR MONOCLONAL ANTIBODIES.

F. A. DAWOOD, M. R. PRICE, M. J. EMBLETON & R. W. BALDWIN, *Cancer Research Campaign Laboratories, University of Nottingham*

Monoclonal antibodies against an osteogenic-sarcoma cell line (791T) were prepared by production and cloning of a somatic-cell hybrid between spleen cells from 791T-immunized mice and the mouse myeloma P3-NS1. Antibodies (IgG2 subclass) produced by 2 clones (36/3 and 48/15) were selected on the basis of their preferential reactivity with osteogenic-sarcoma cells, in the $^{125}$I-Protein A cell-binding assay (Embleton *et al.*, 1981, *Br. J. Cancer*, **43**, 582). After purification of antibodies by their affinity to Sepharose-Protein A, the preparations were radiolabelled with $^{125}$I using chloramine T. The pattern of rebinding of labelled monoclonal antibodies to tumour cell lines was similar to that demonstrated using the $^{125}$I-Protein A cell-binding test, though the sensitivity of the direct binding test was less than that of the indirect-binding assay. Cold-antibody inhibition of binding of labelled antibodies established that the 2 antibodies from hybridomas 36/3 and 48/15 detected 2 different surface antigens on 791T cells. Serum samples of known antibody reactivity from the 791T sarcoma donor failed to inhibit the binding of radiolabelled monoclonal antibody, suggesting that autochthonous host reactivity was directed against different antigenic targets.

### A COMPARISON OF THE CYTOTOXIC EFFECTS OF ADRIAMYCIN AND mAMSA ON MAMMALIAN CELLS *IN VITRO*.

C. WEST, E. SMITH, N. BARRASS, I. STRATFORD & G. ADAMS, *Physics Department, Institute of Cancer Research, Sutton, Surrey*

Adriamycin (ADM) and the anilinoacridine mAMSA are thought to be cytotoxic because of their ability to interchelate between adjacent base pairs in DNA. Whereas ADM has been in use for many years, mAMSA is only in the initial stages of clinical testing, and has been considered by some as an alternative to ADM.

In this work we have compared the actions of ADM and mAMSA in Chinese hamster V79 cells *in vitro*, using cell survival and sister-chromatid exchange as end-points. Equimolar concentrations of ADM and mAMSA show similar toxicities towards exponentially growing cells, and both drugs are less effective in killing chronically hypoxic and plateau-phase cells. Cytotoxicity to thermotolerant cells (41°C for 16 h previously) and cells held at a low pH, show little difference from that for exponential cells. Pretreating cells with misonidazole under hypoxic conditions reduces the toxicity of both ADM and mAMSA. In addition, an ADM resistant cell line, V79–177 (Harris *et al.*, 1979, *Int. J. Radiat. Oncol. Biol. Phys.*, **5**, 1235) was cross-resistant to mAMSA. Finally, low equimolar doses of both drugs were found to cause similar increases in the levels of SCE in V79 cells.

**THE CYTOTOXIC AND RADIOSENSITIZING EFFECTS OF Rh(II) CARBOXYLATES.** R. CHIBBER, I. STRATFORD, B. LEE & G. ADAMS, *Physics Department, Institute of Cancer Research, Sutton, Surrey, and School of Natural Sciences, Hatfield Polytechnic, Hatfield, Herts*

Three Rh(II) carboxylates have been synthesized and tested as cytotoxic and radiosensitizing agents in Chinese hamster V79 cells *in vitro*. These compounds were the butyrate, propionate, and acetate. Survival curves were generated for each compound as a function of drug concentration, contact time and temperature. These data showed that toxicity was in the order butyrate > propionate > acetate. The magnitude of the differences in toxicity is indicated by the concentration of each compound required to give a surviving fraction of 0·1 in 2 h; *viz.*: $4{\cdot}2\times10^{-7}$, $4{\cdot}5\times10^{-6}$ and $4{\cdot}4\times10^{-5}$M for the butyrate, propionate and acetate respectively. For comparison $10^{-5}$ *cis*-$PtCl_2$ $(NH_3)_2$ is required to give the same level of toxicity. All 3 compounds studied showed greater toxicity to hypoxic cells. Of the 3 carboxylates, *only* the acetate showed any ability to radiosensitize hypoxic cells. At non-toxic concentrations, enhancement ratios up to 1·5 were obtained.

These results suggest that the mechanism(s) of toxicity and radiosensitization by the Rh(II) carboxylates are different.


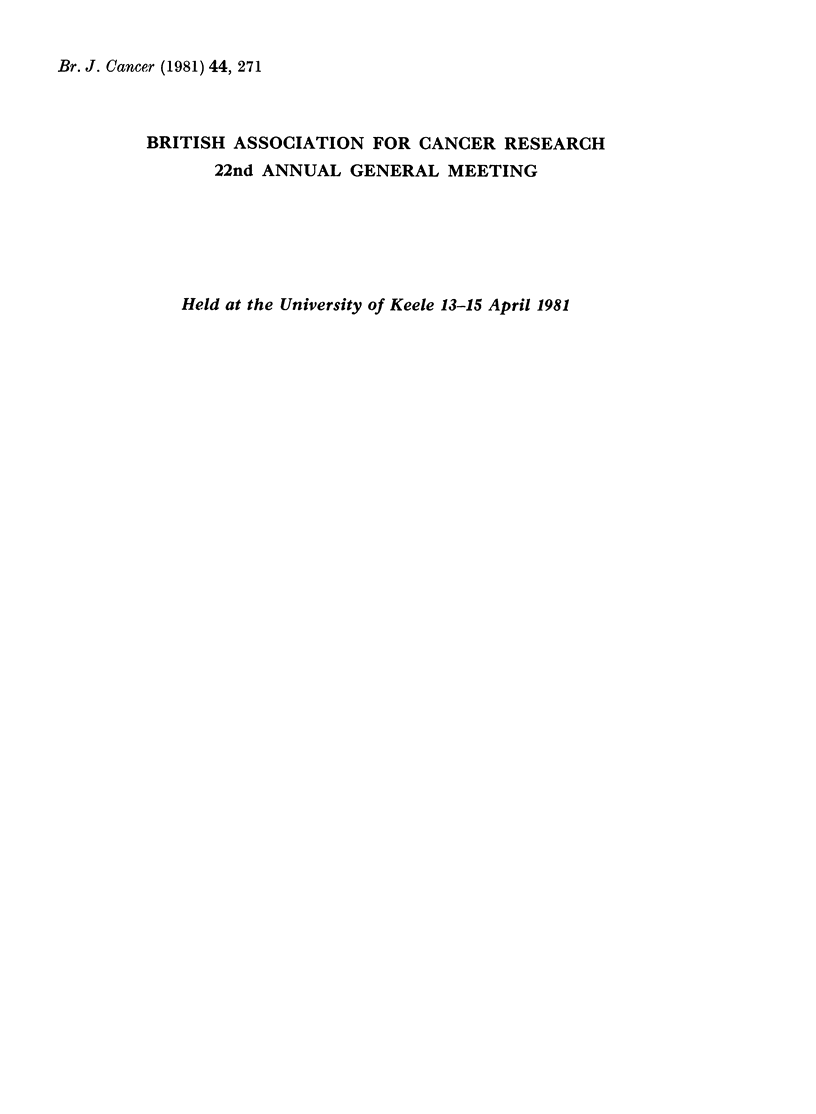

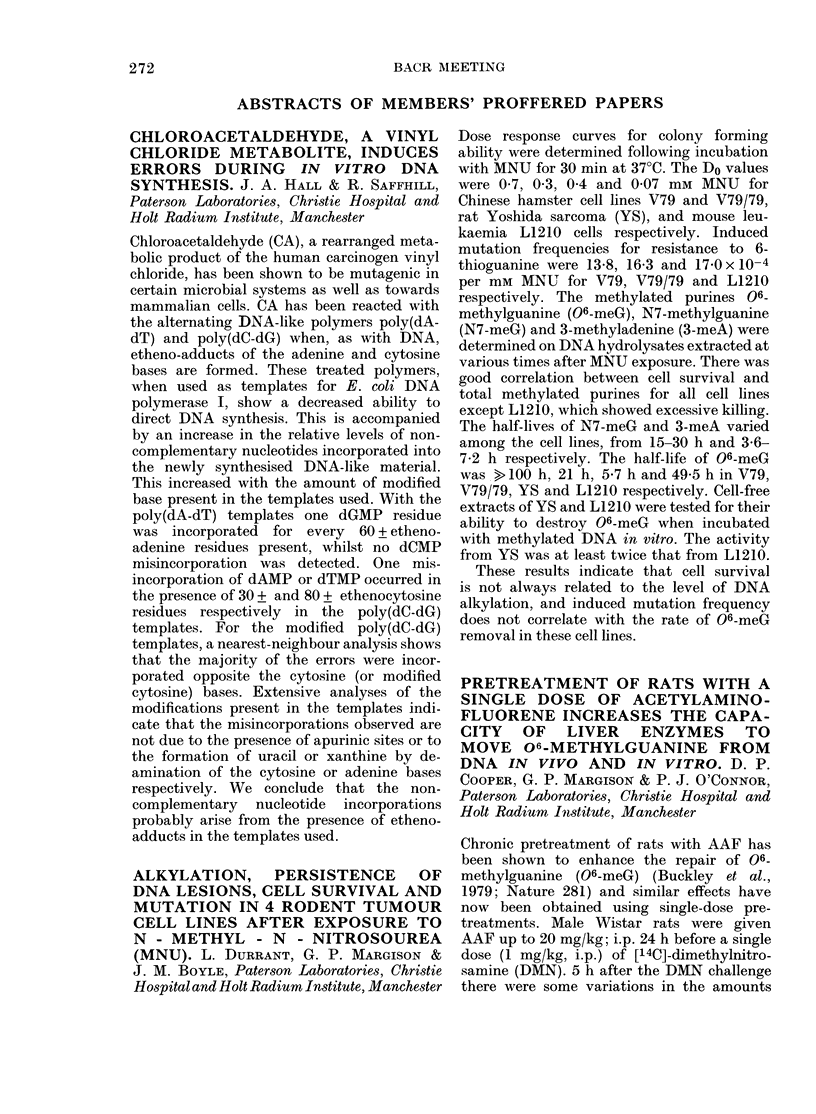

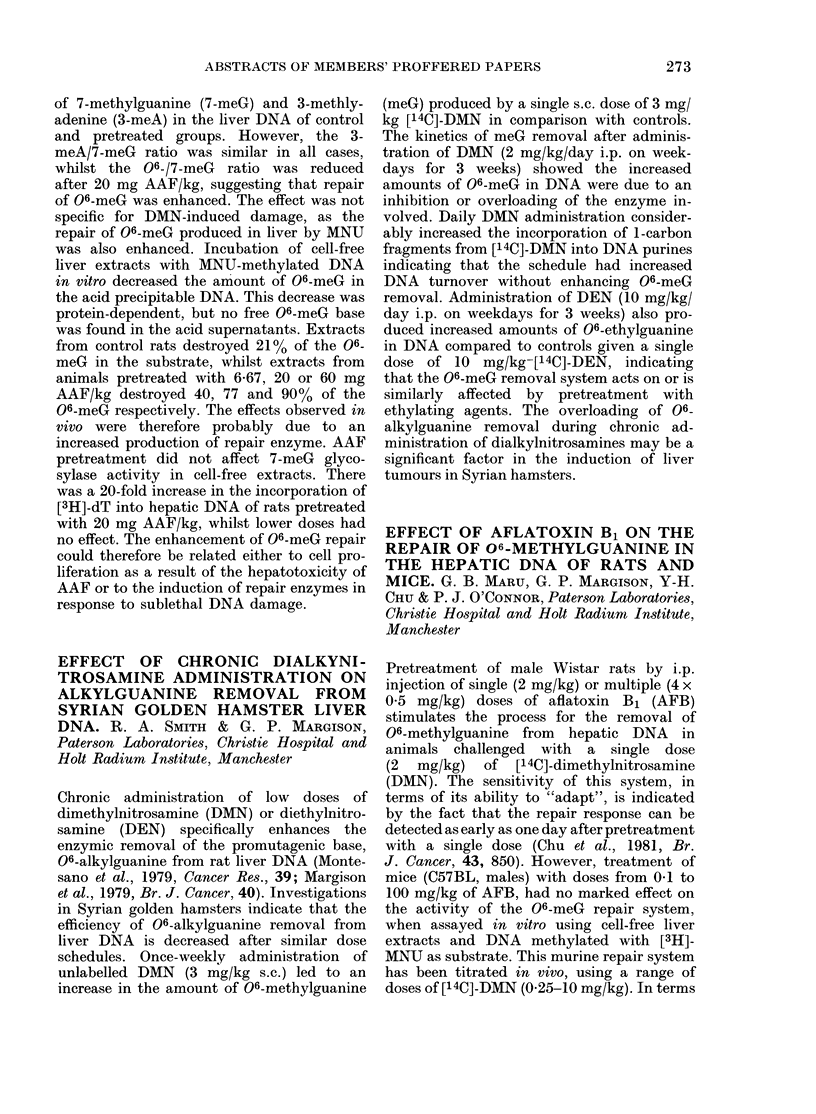

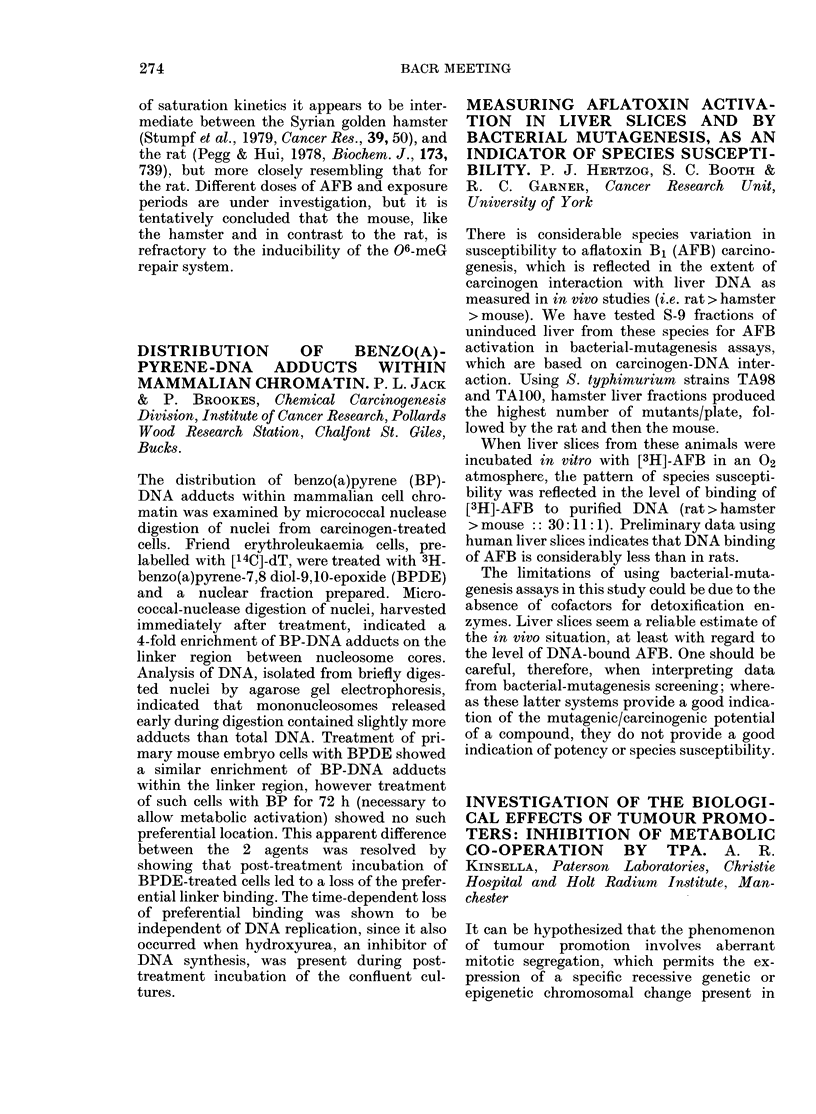

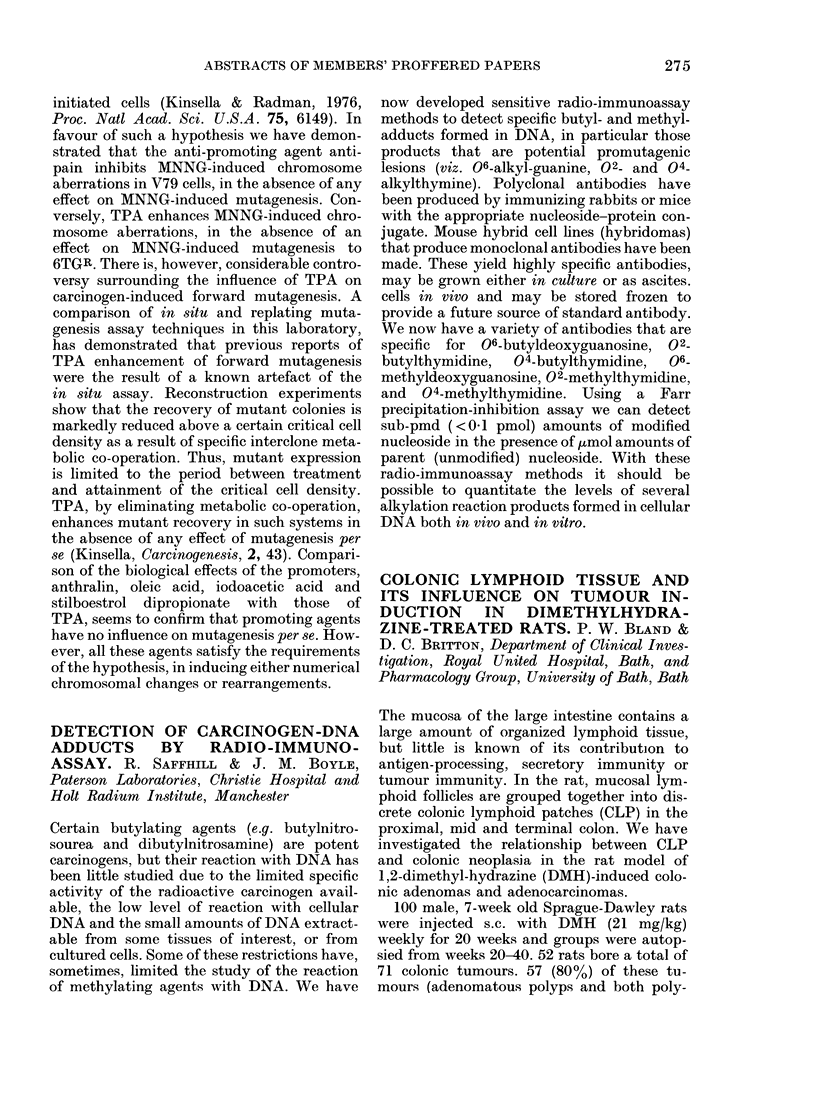

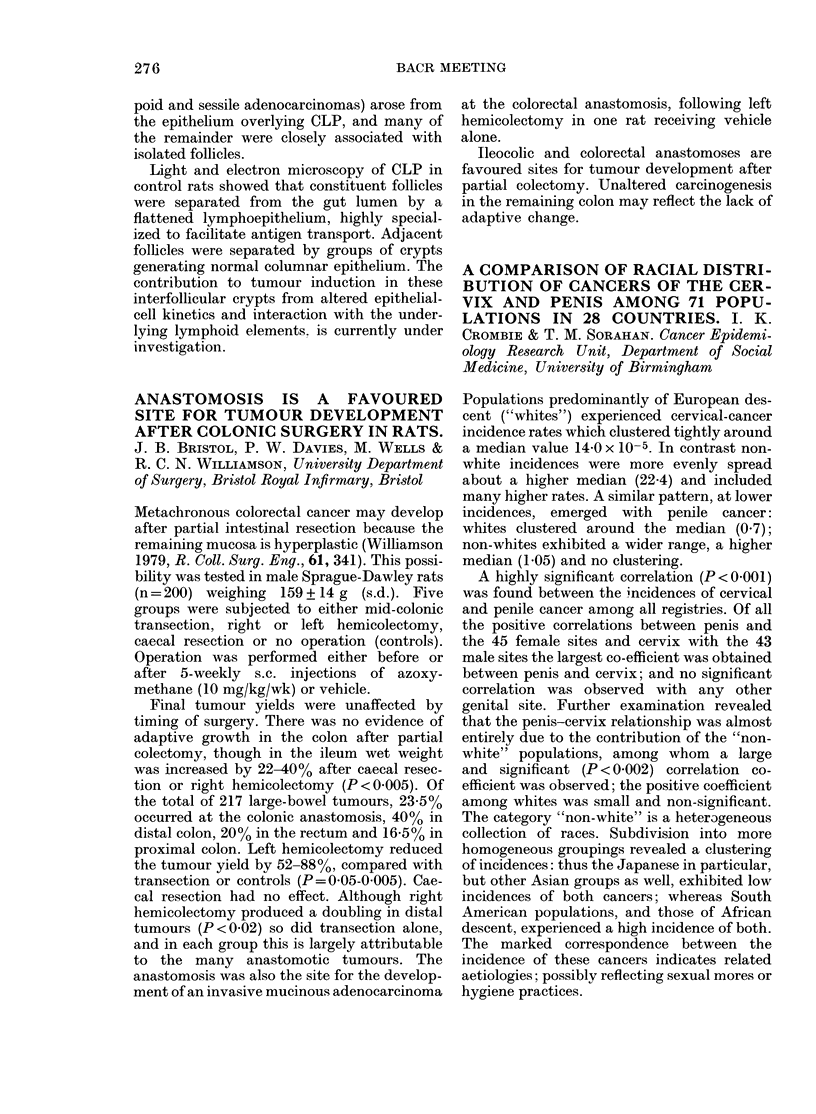

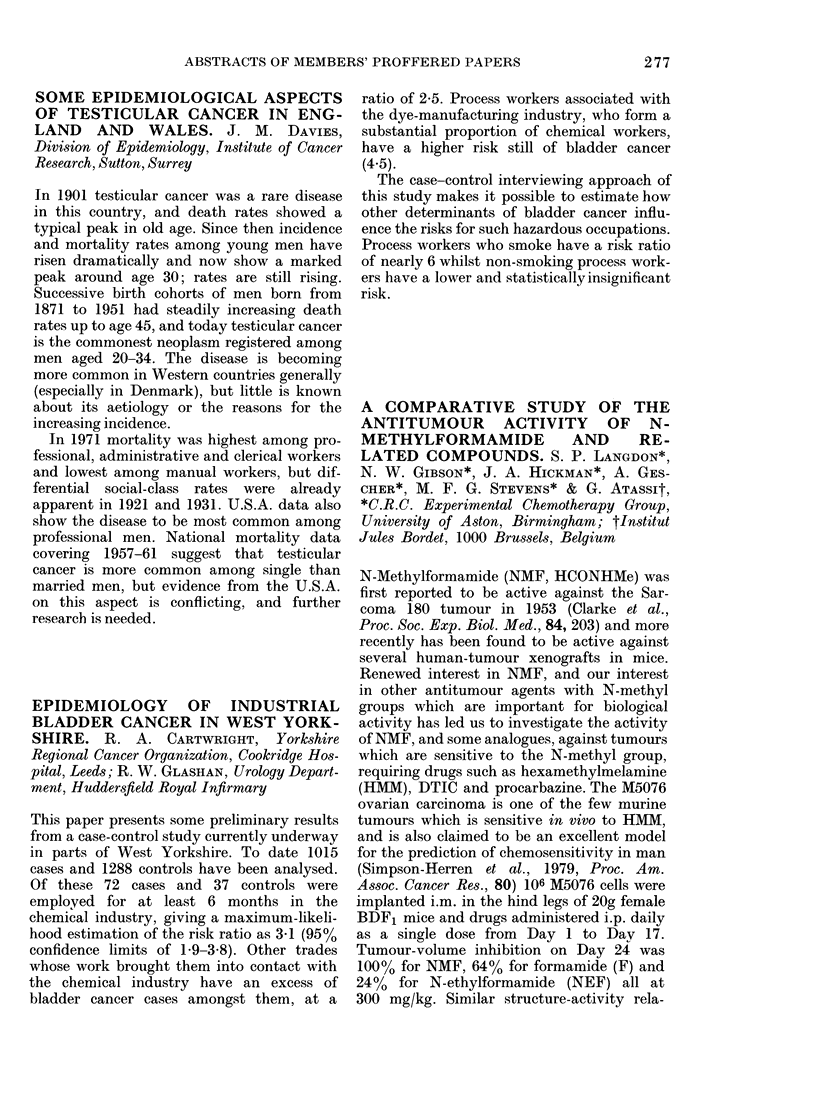

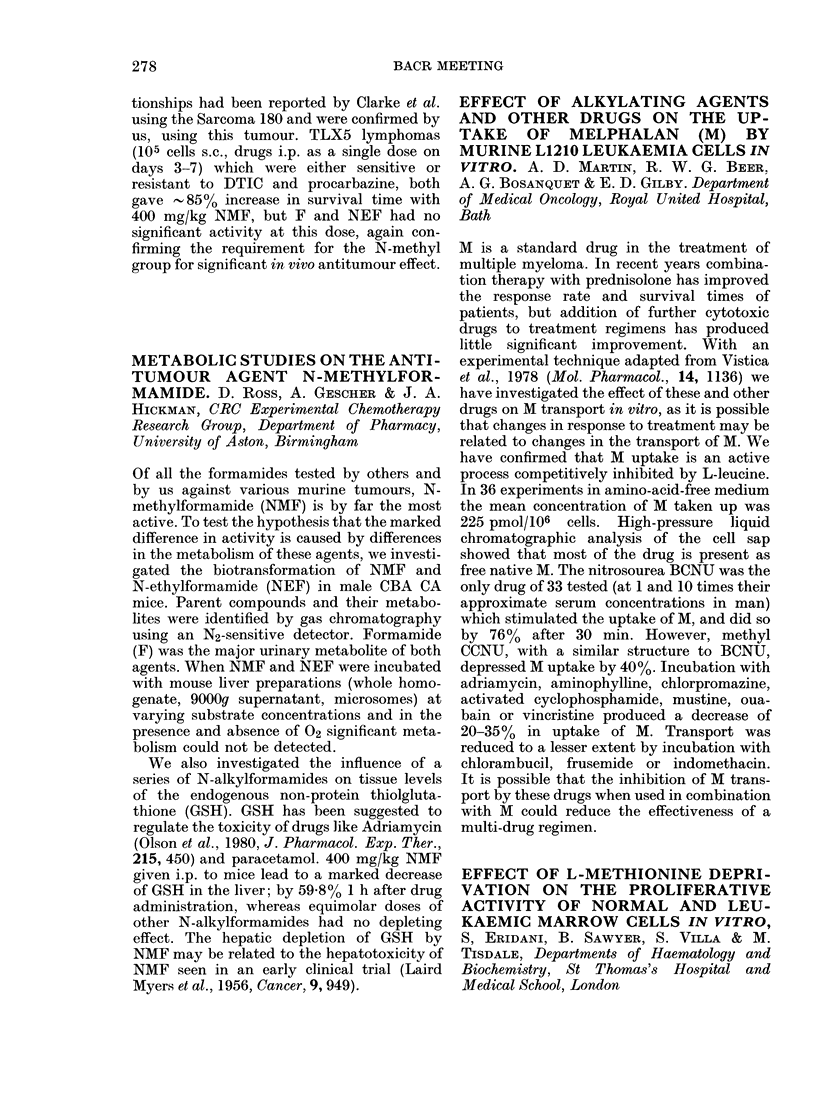

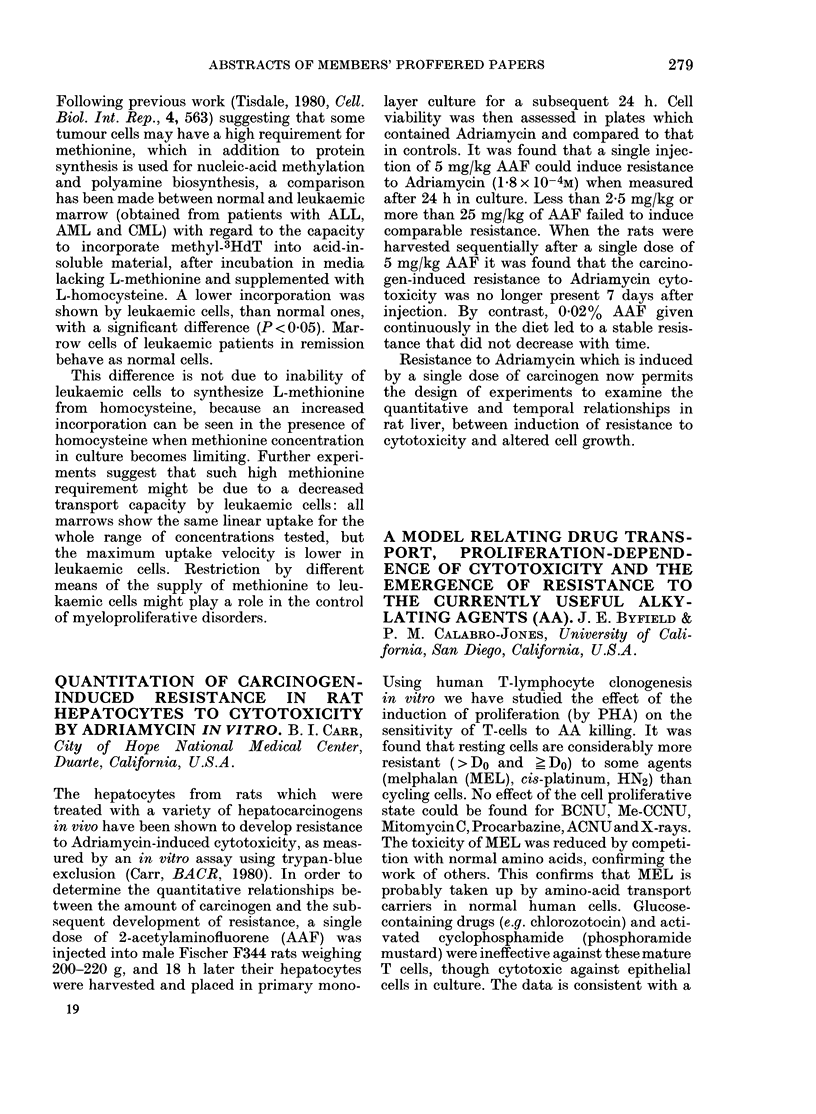

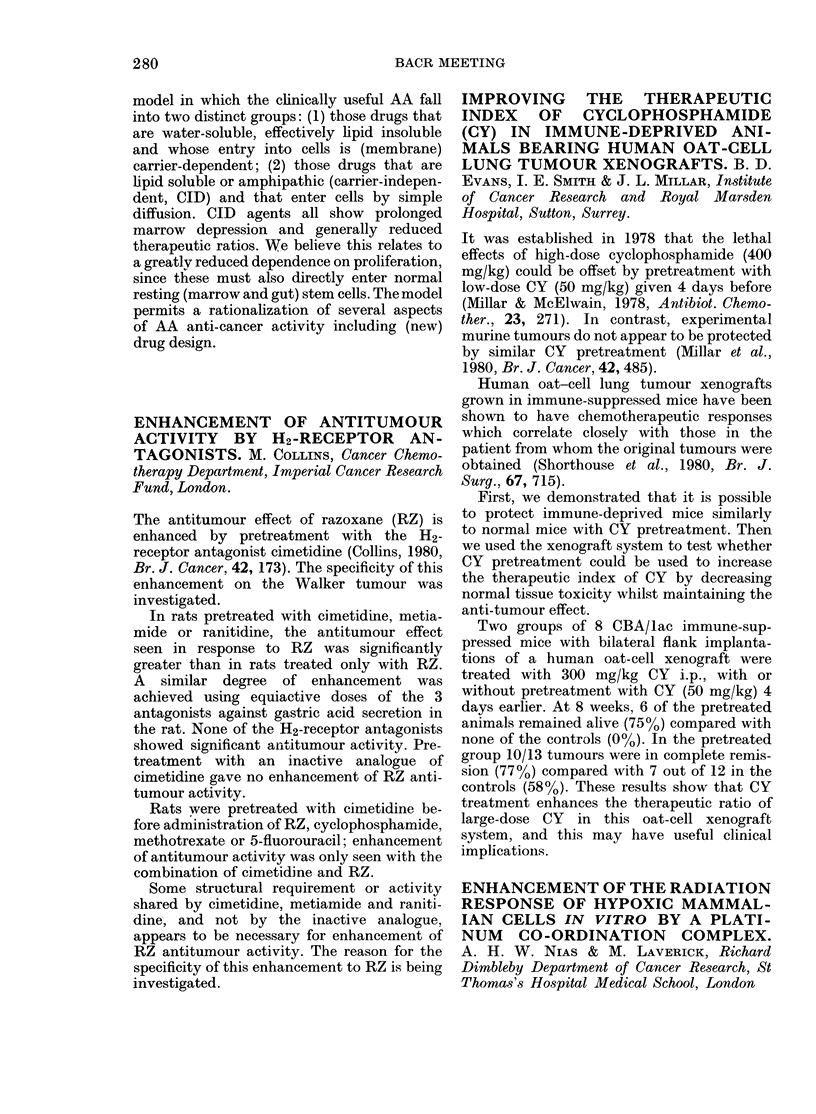

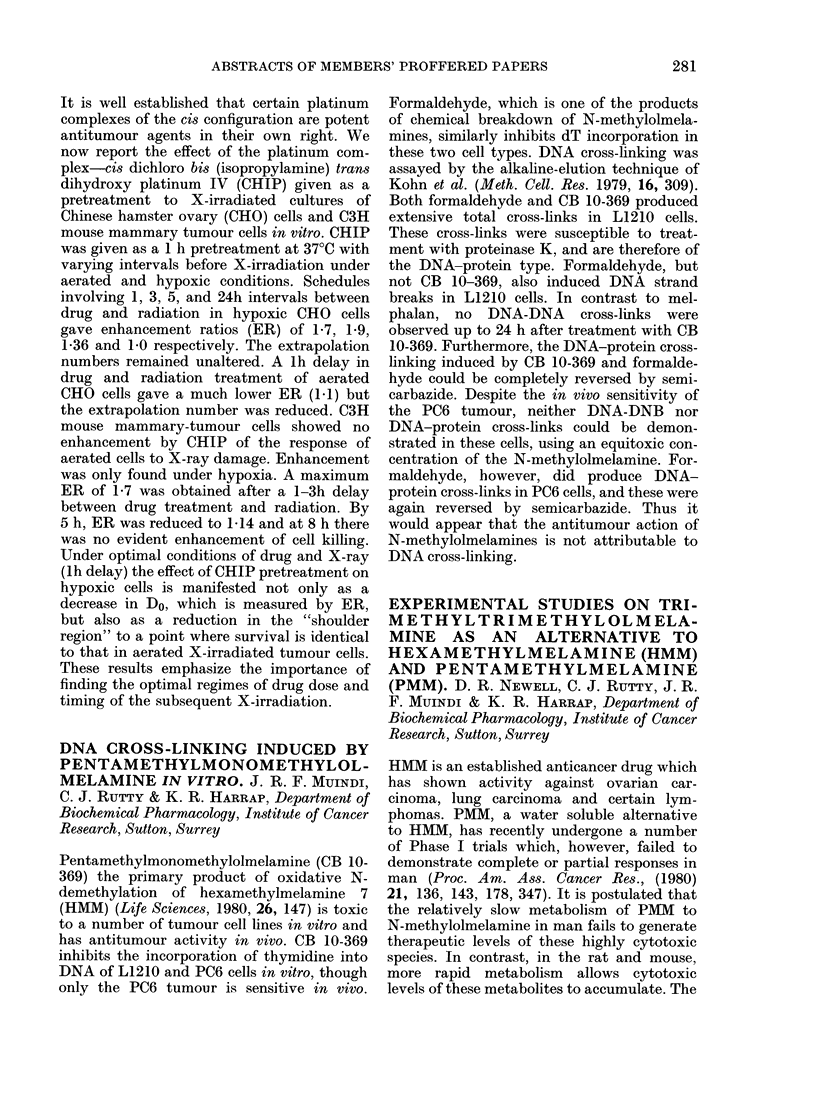

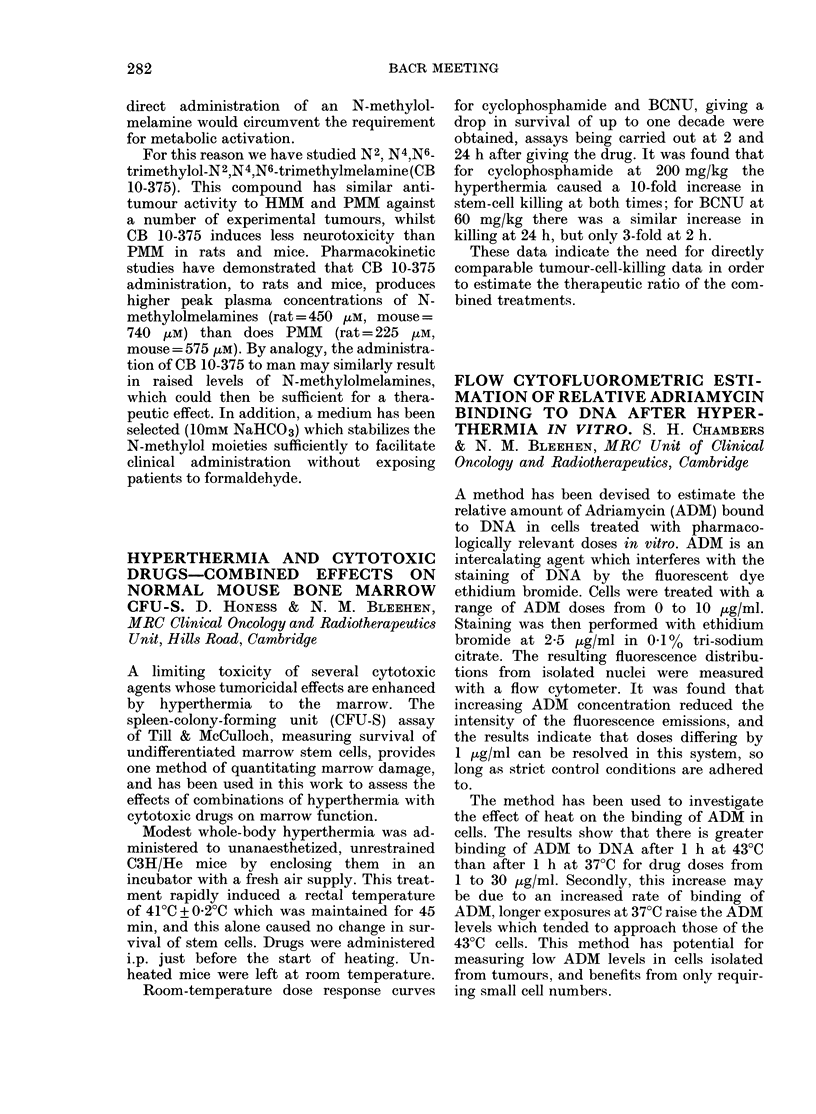

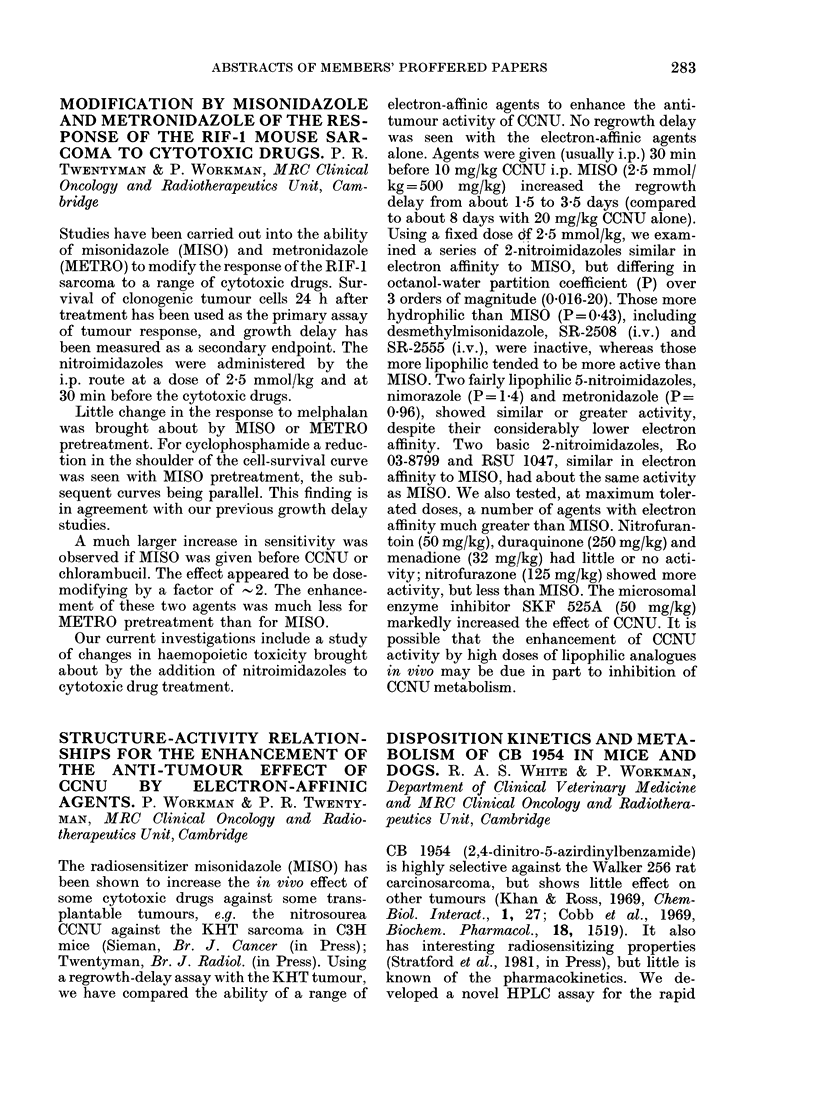

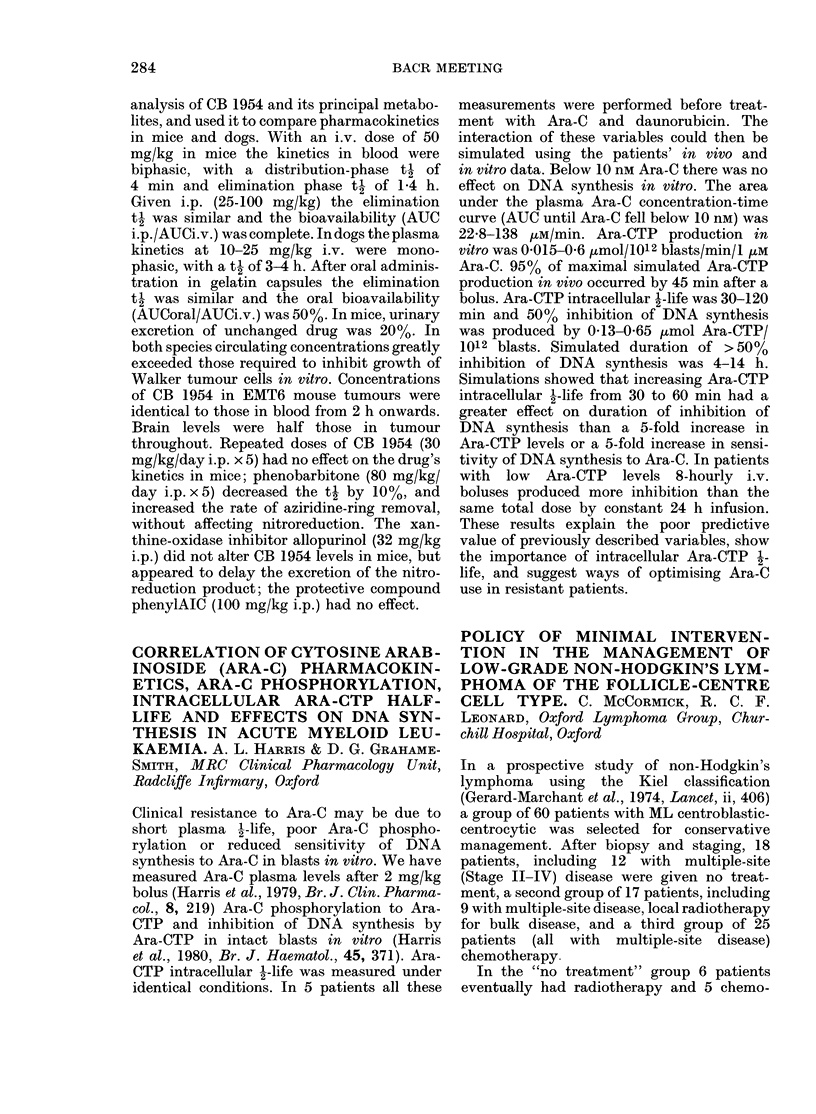

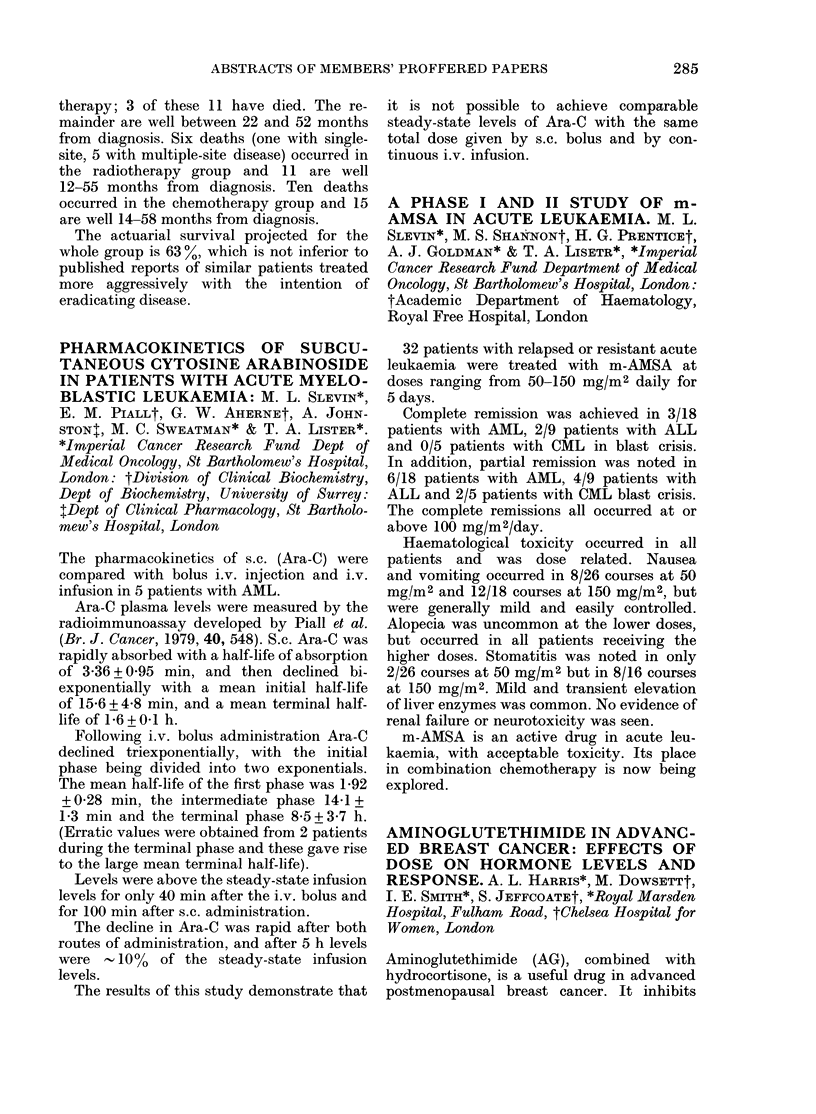

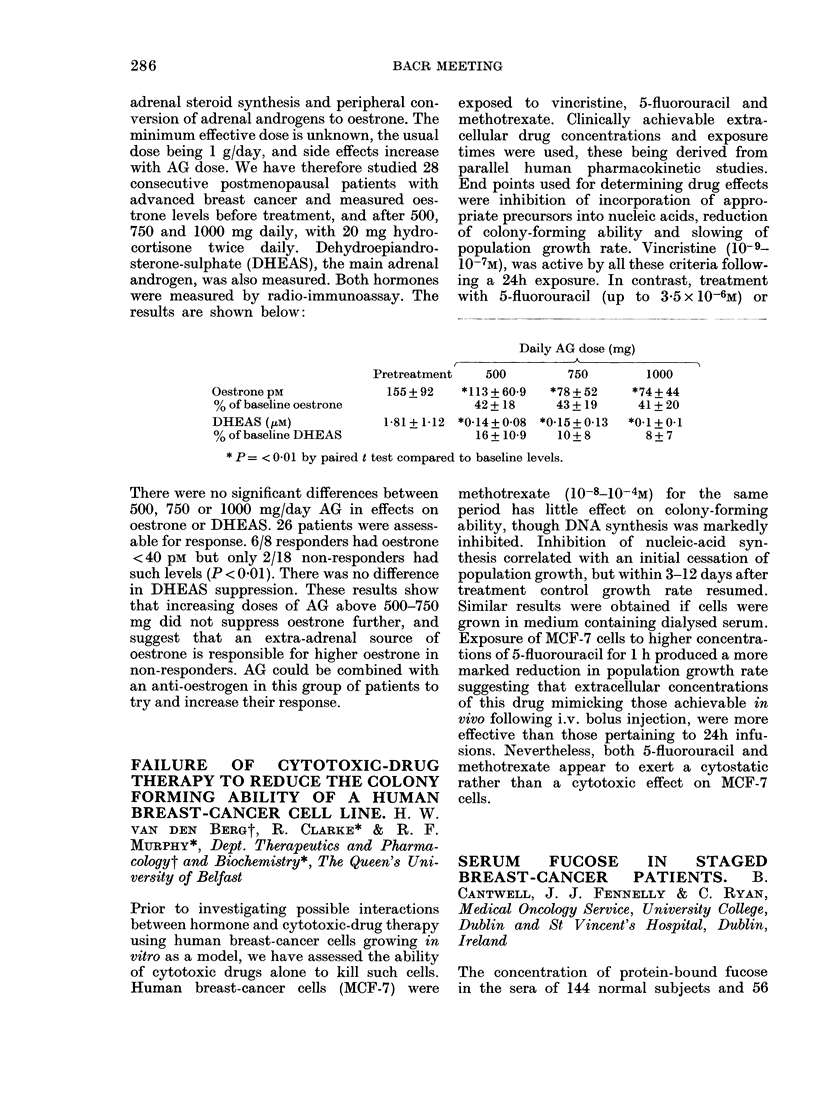

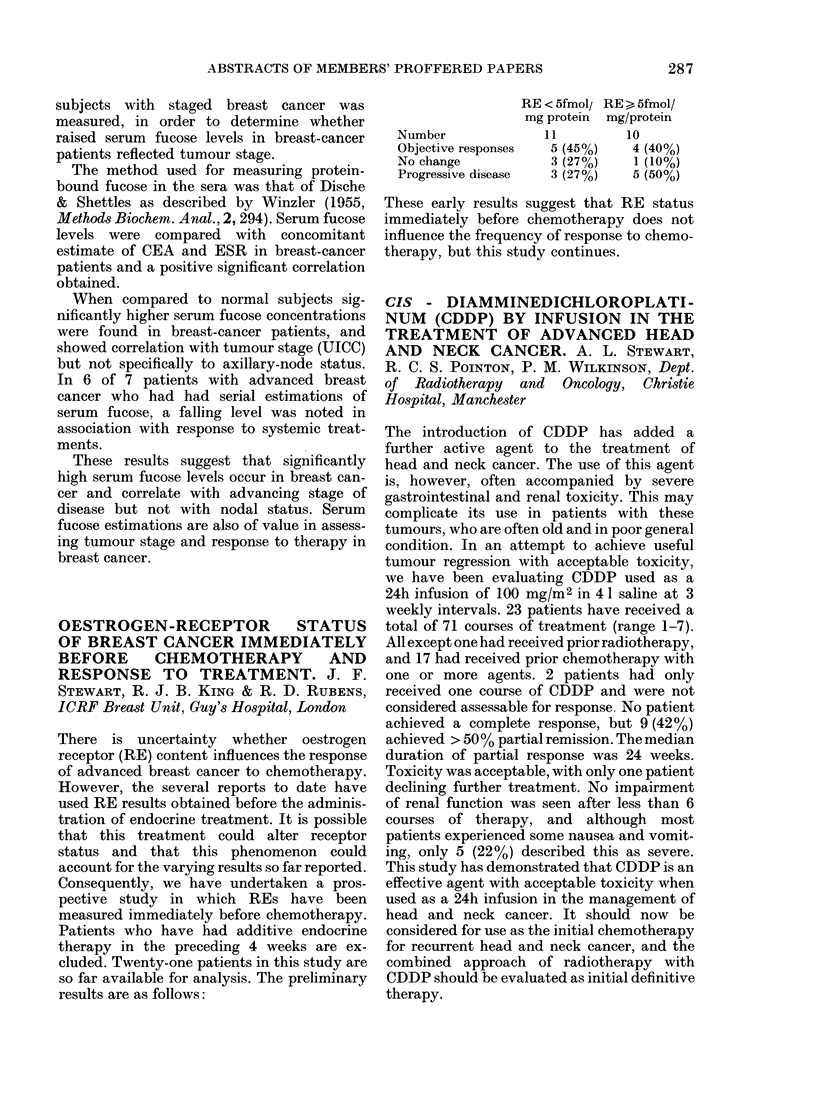

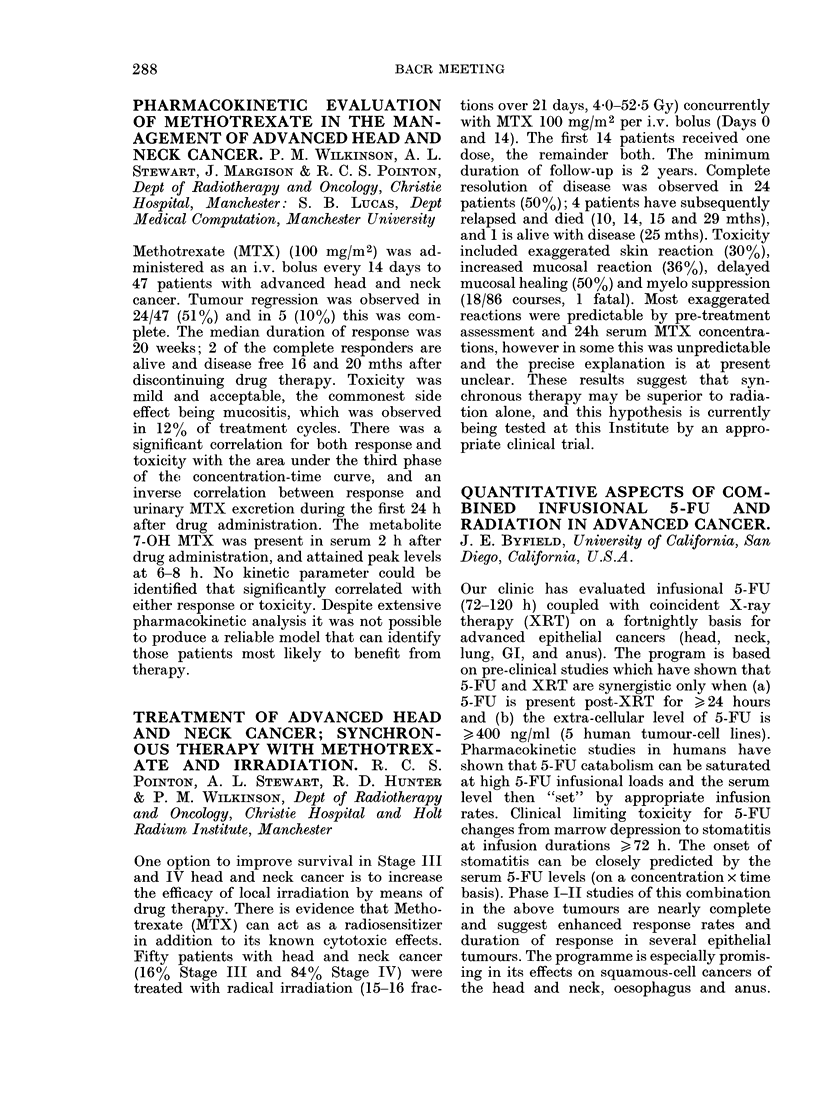

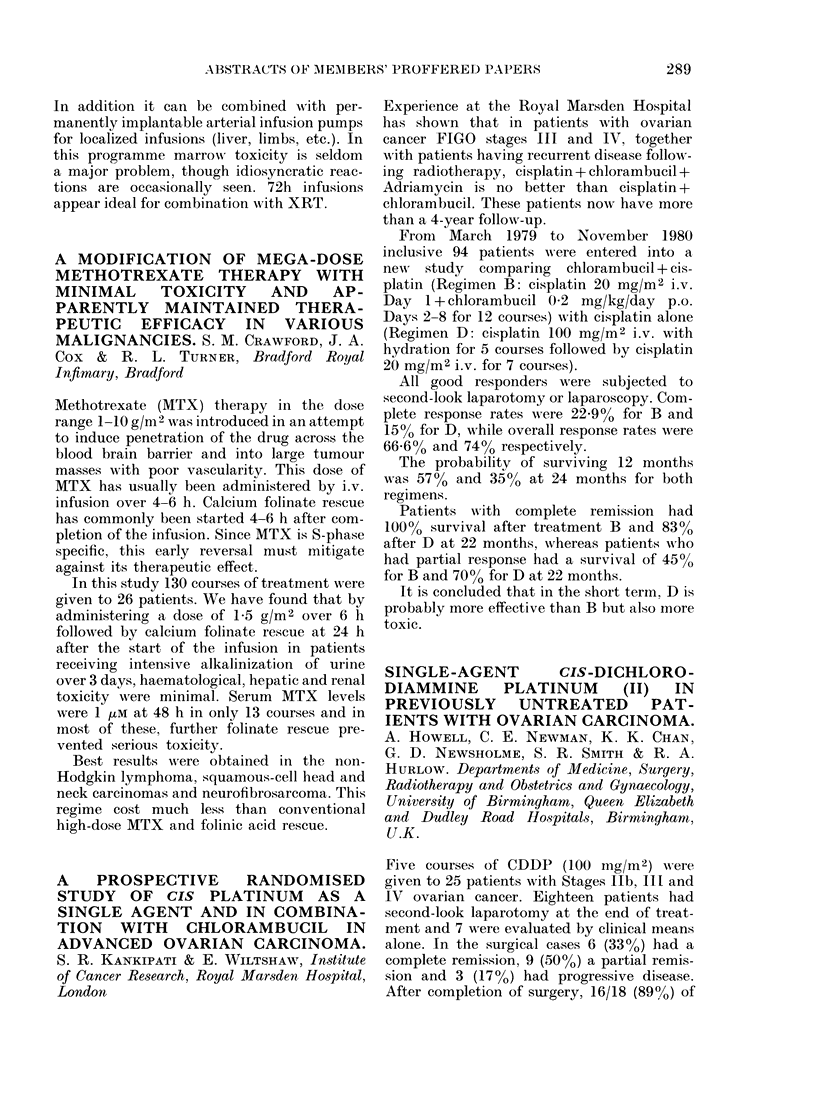

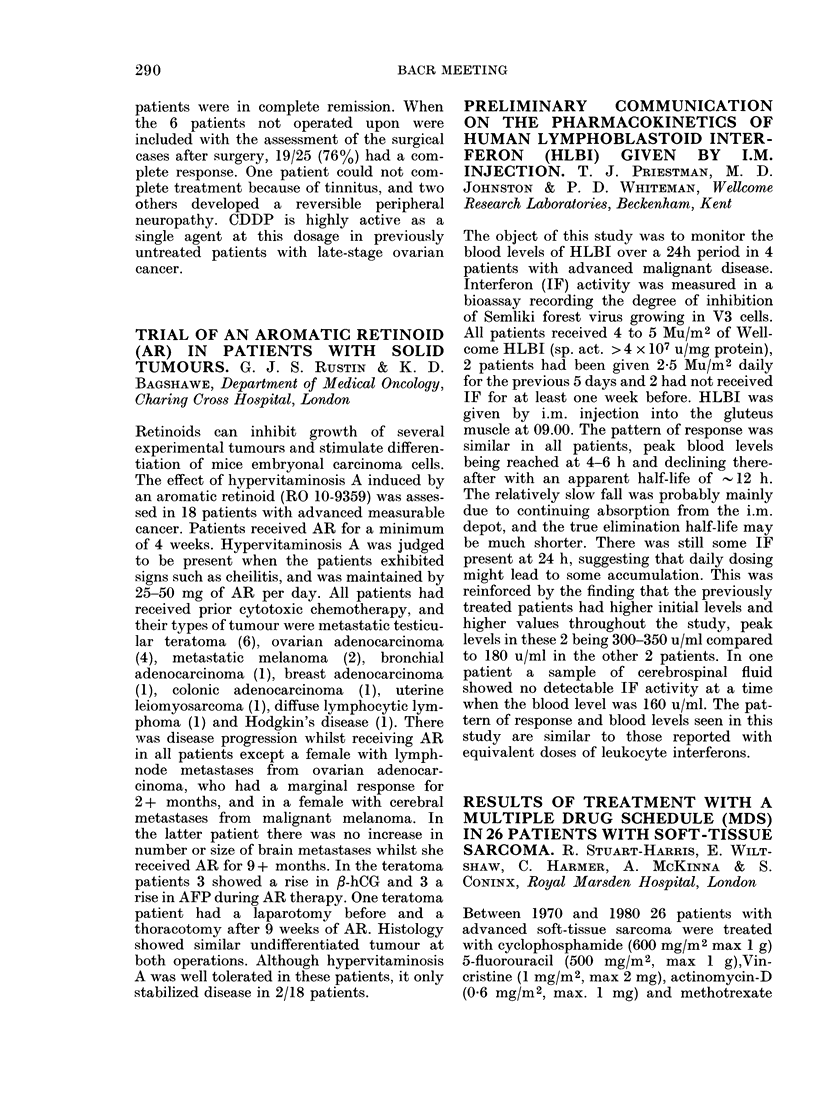

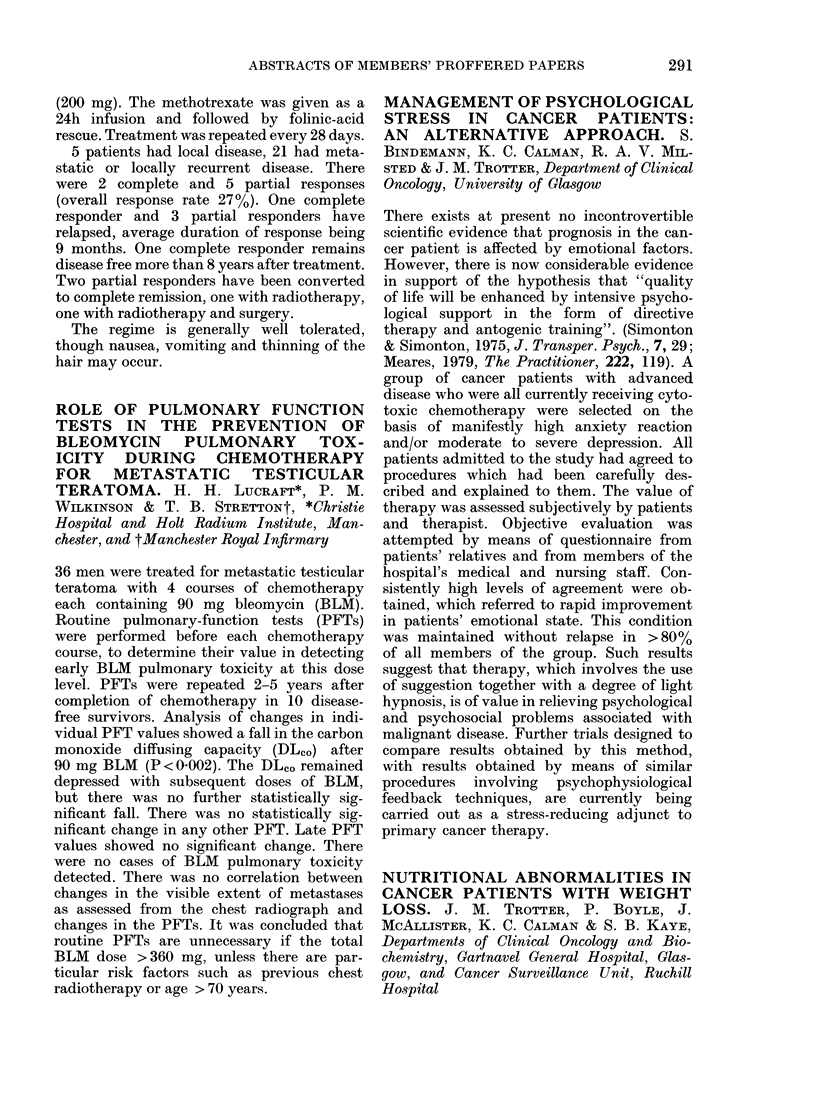

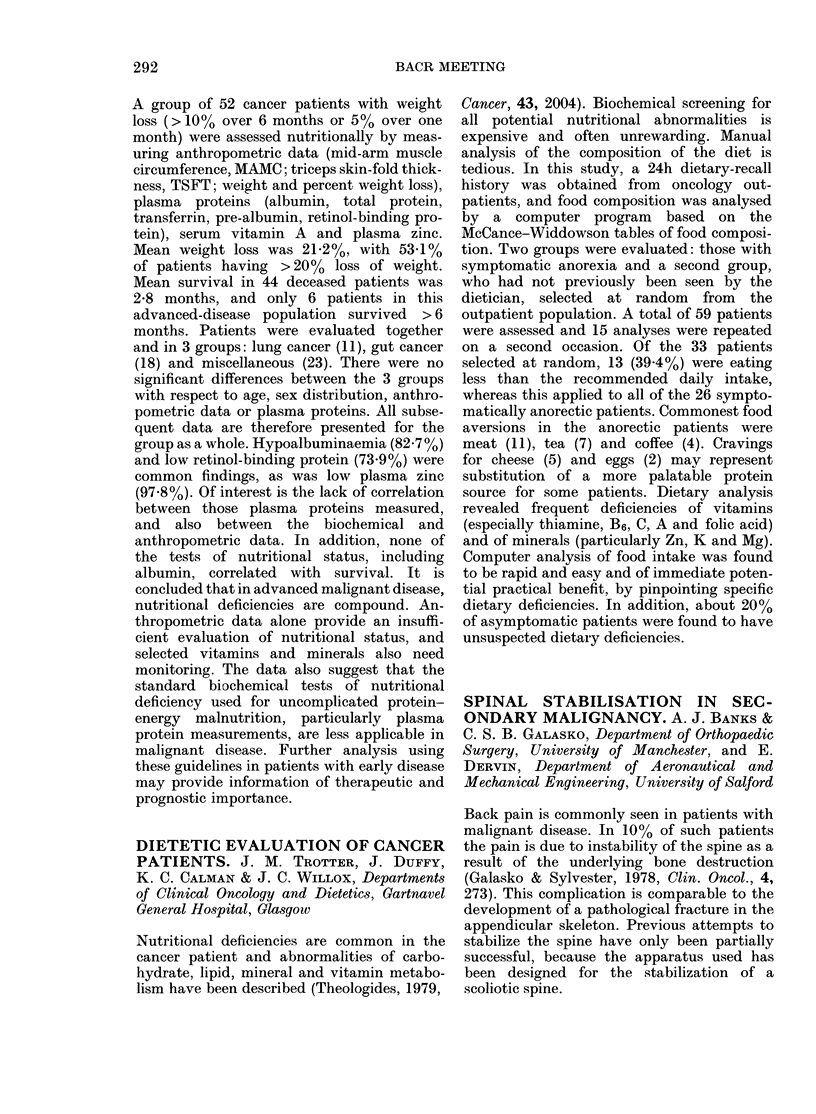

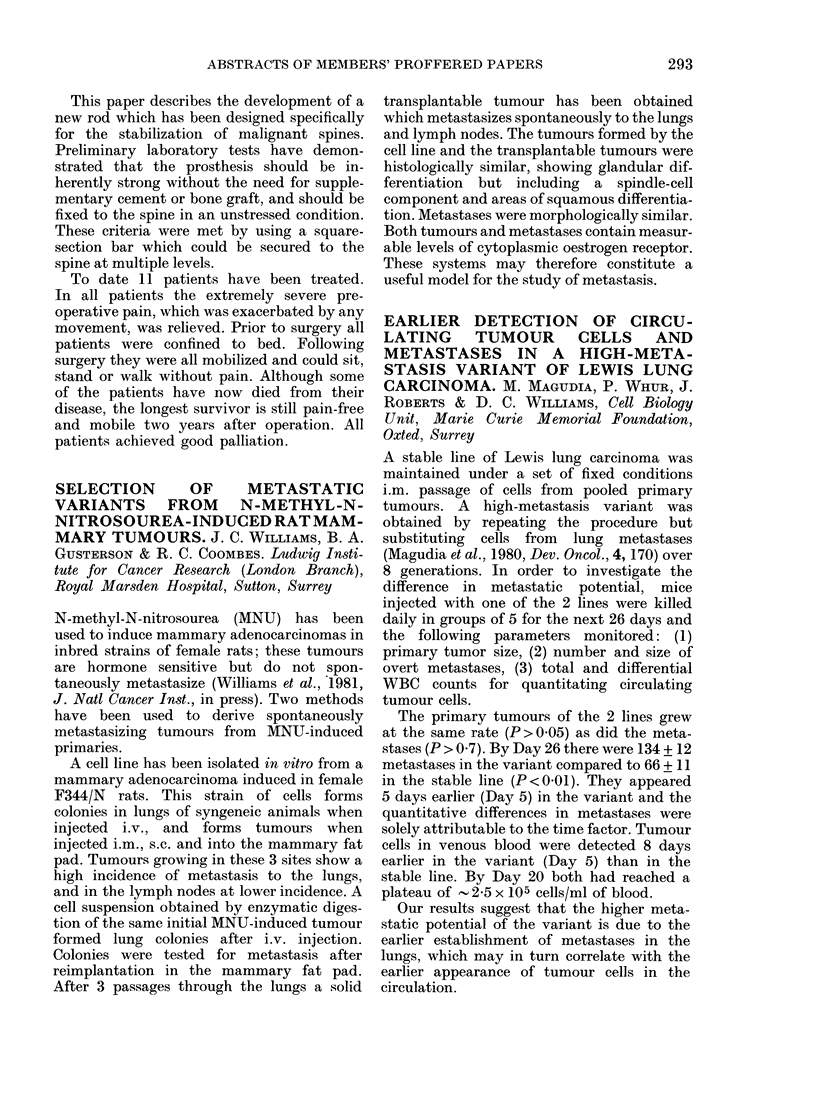

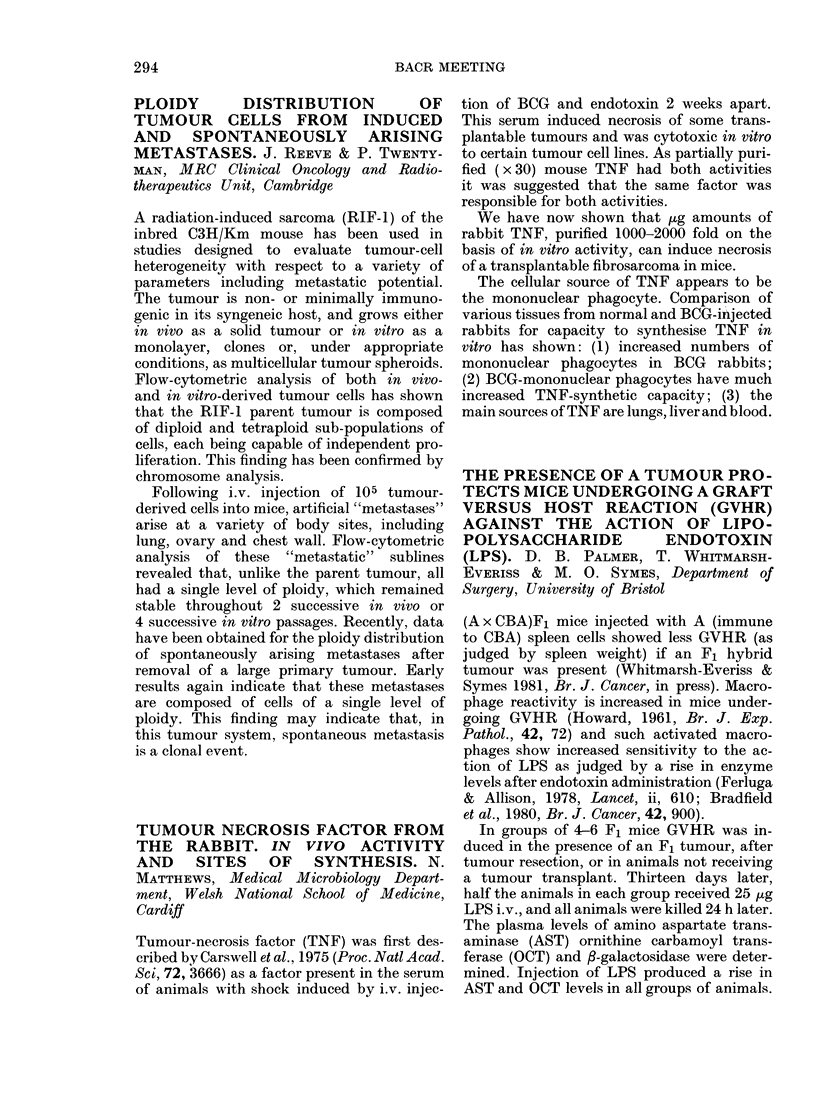

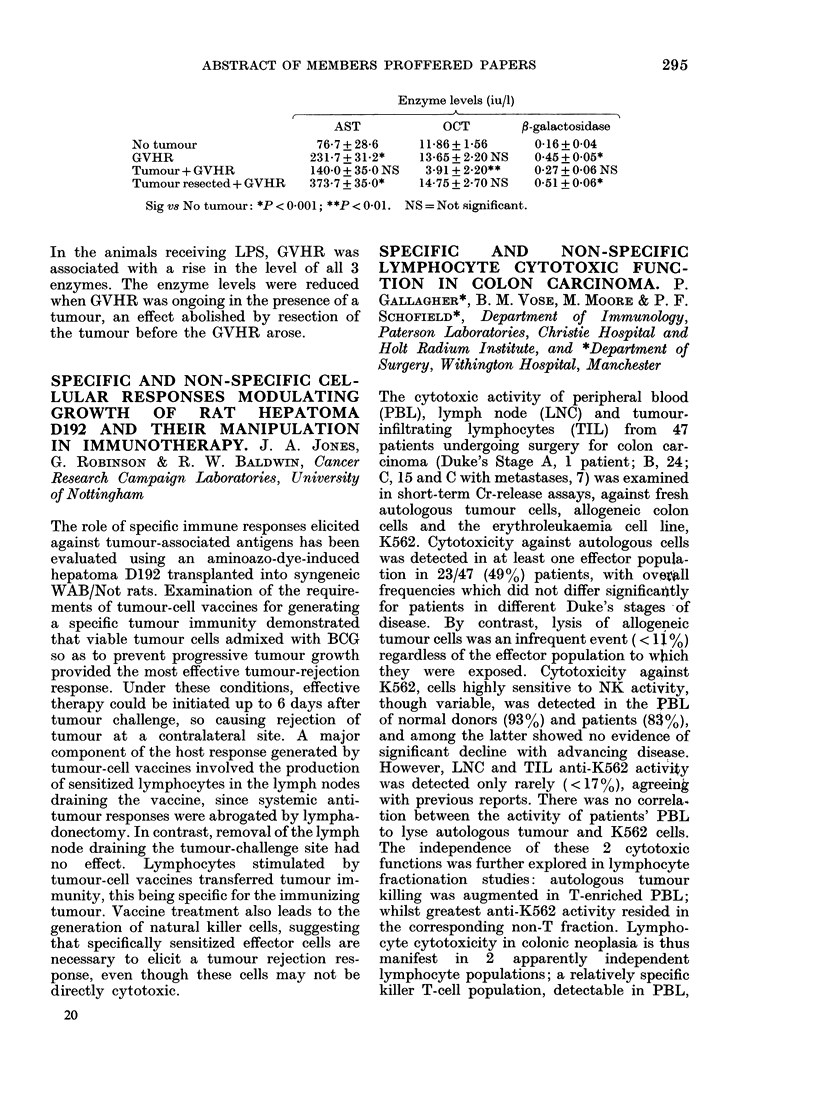

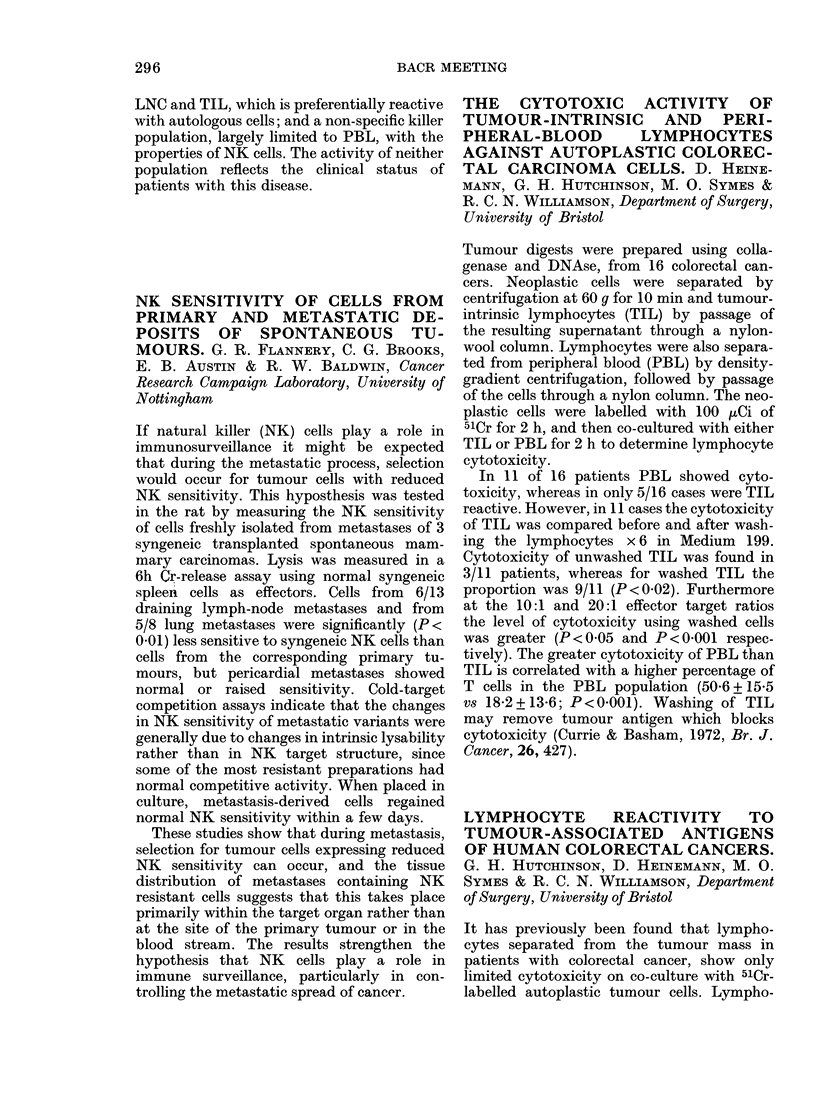

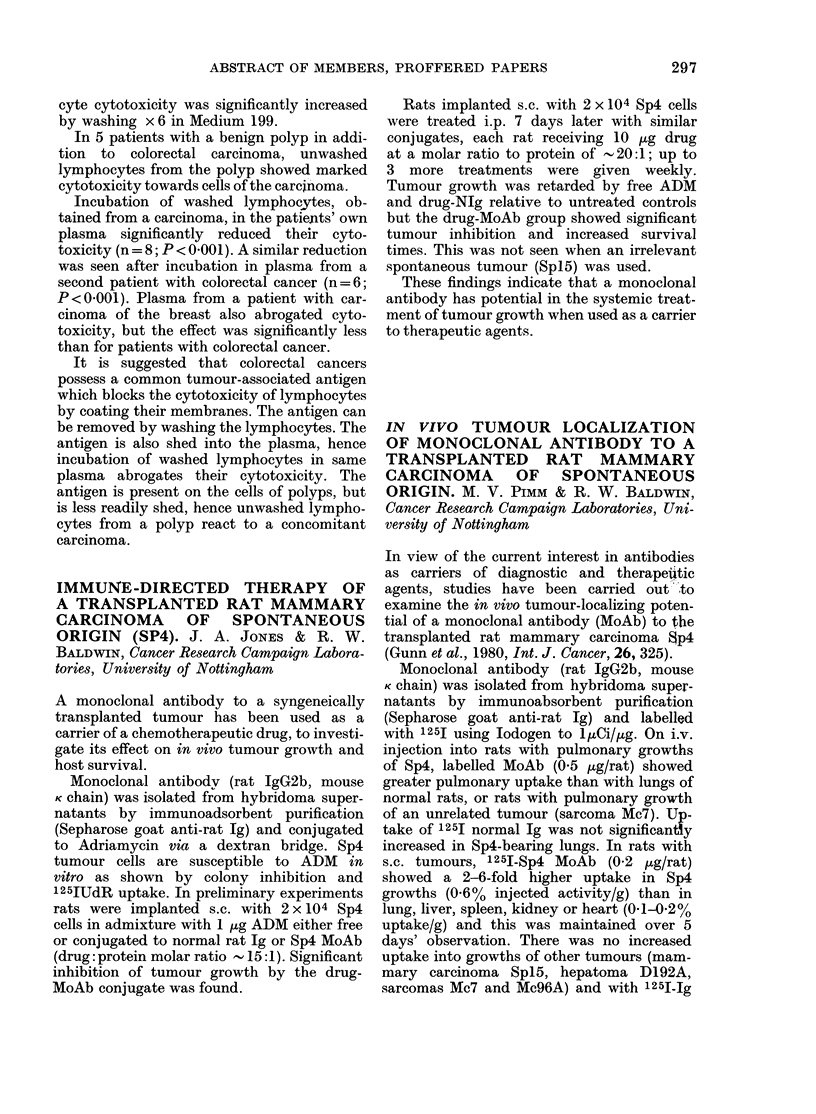

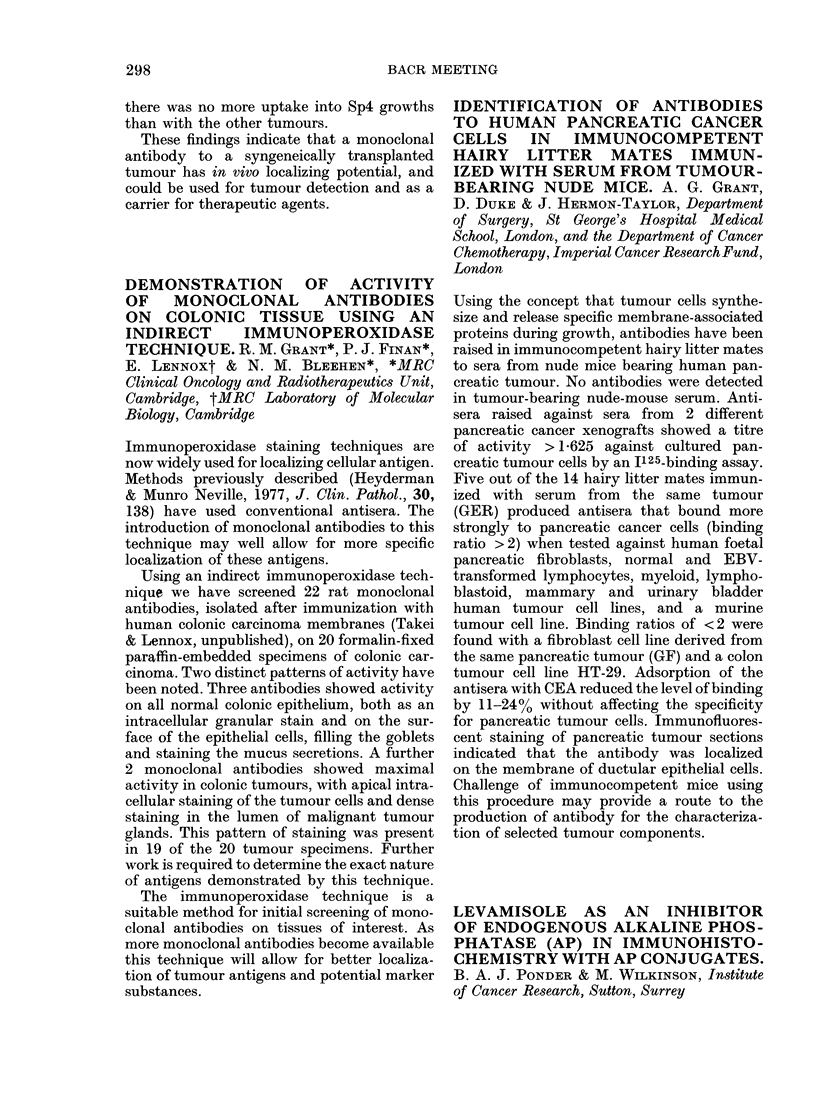

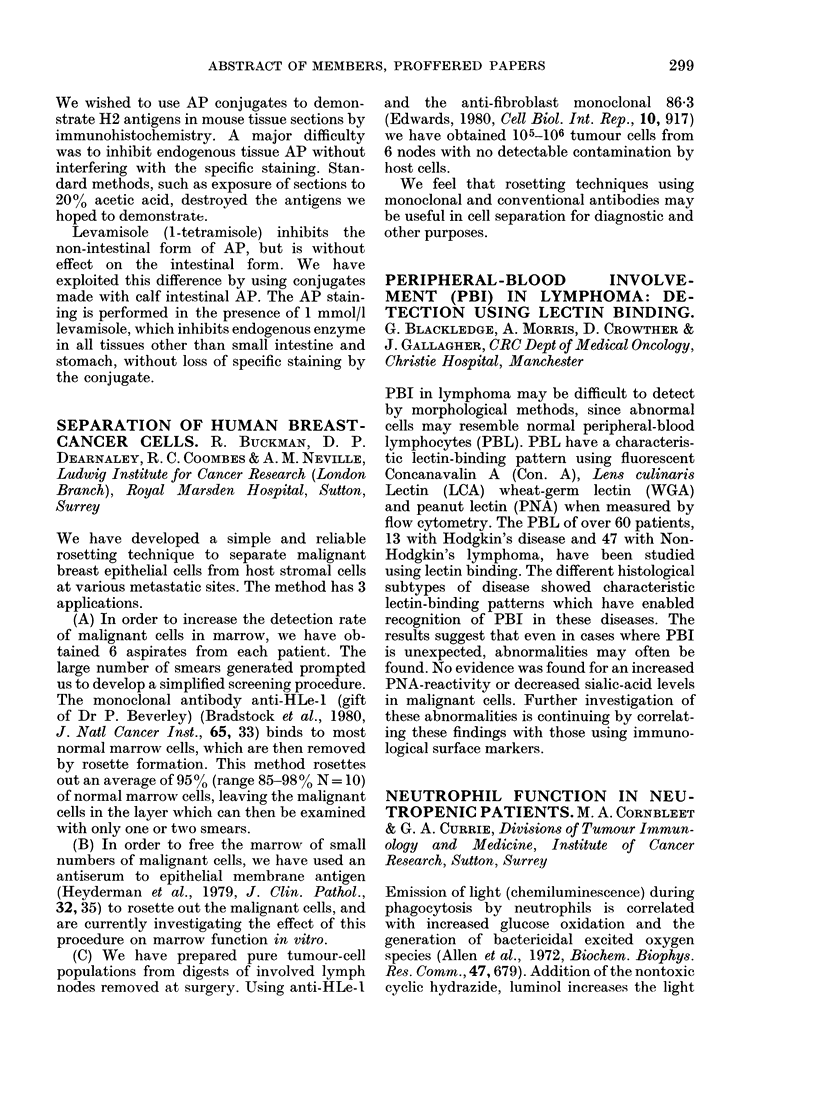

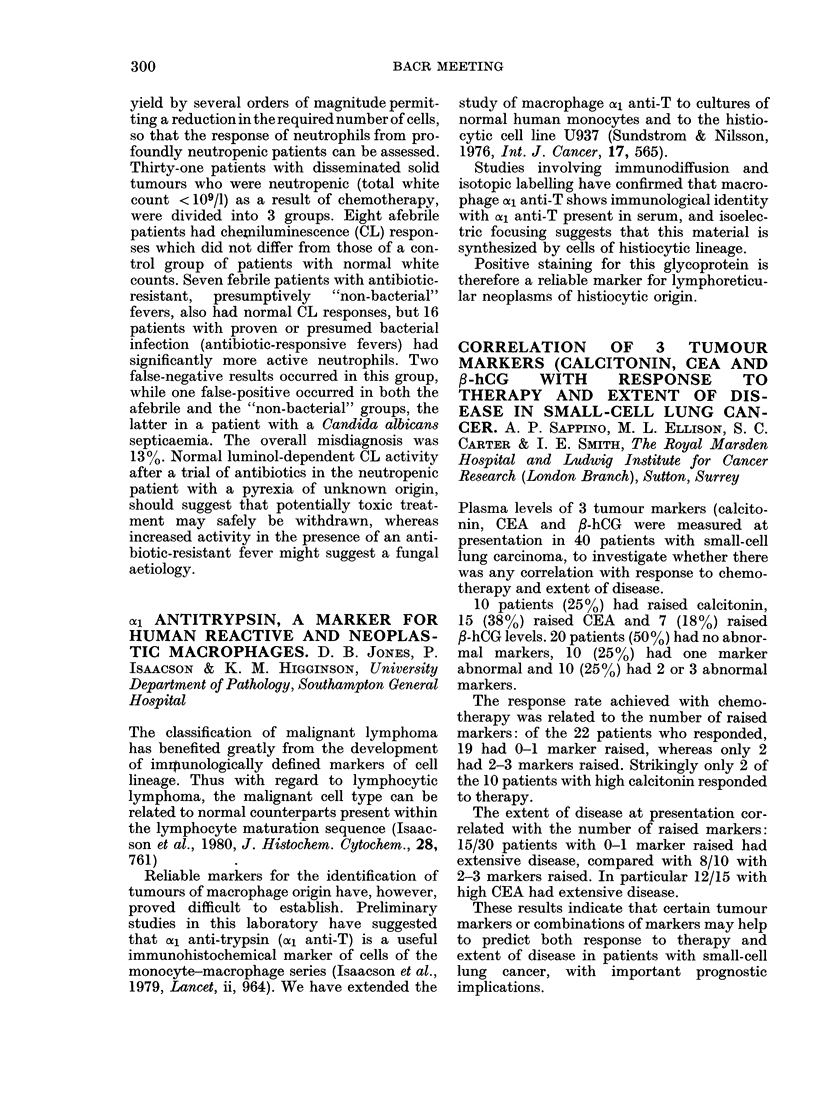

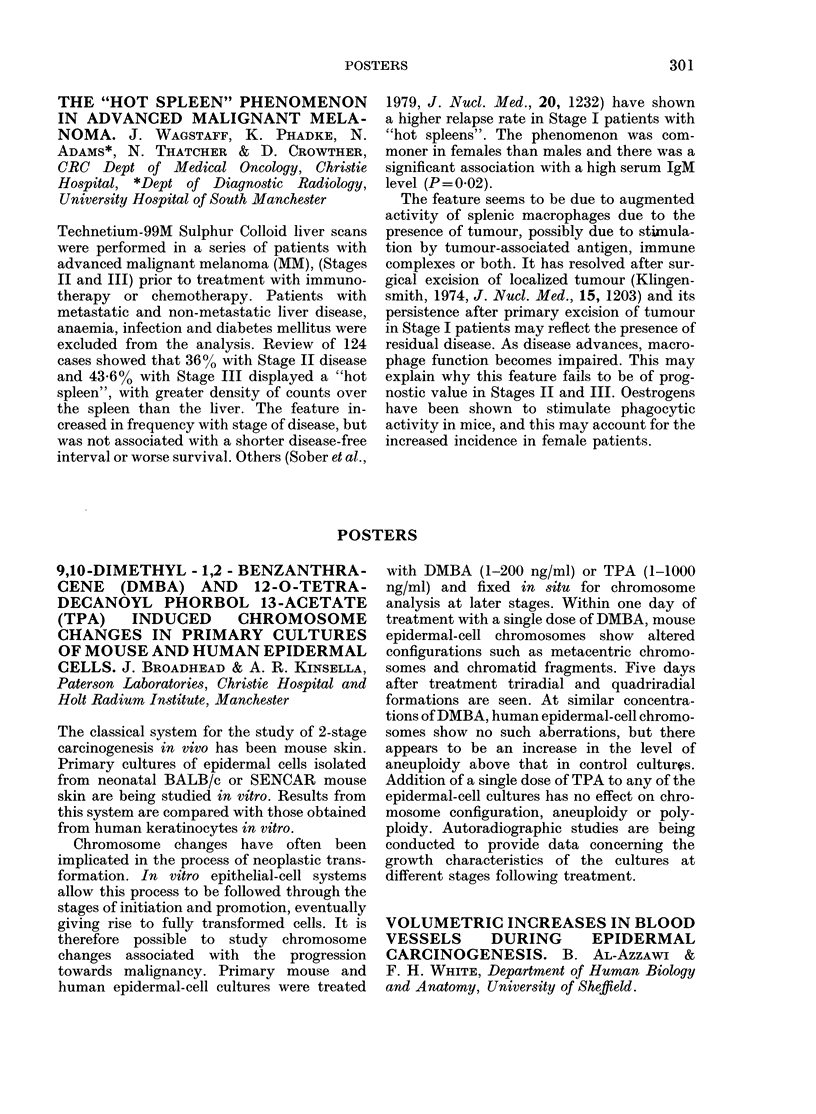

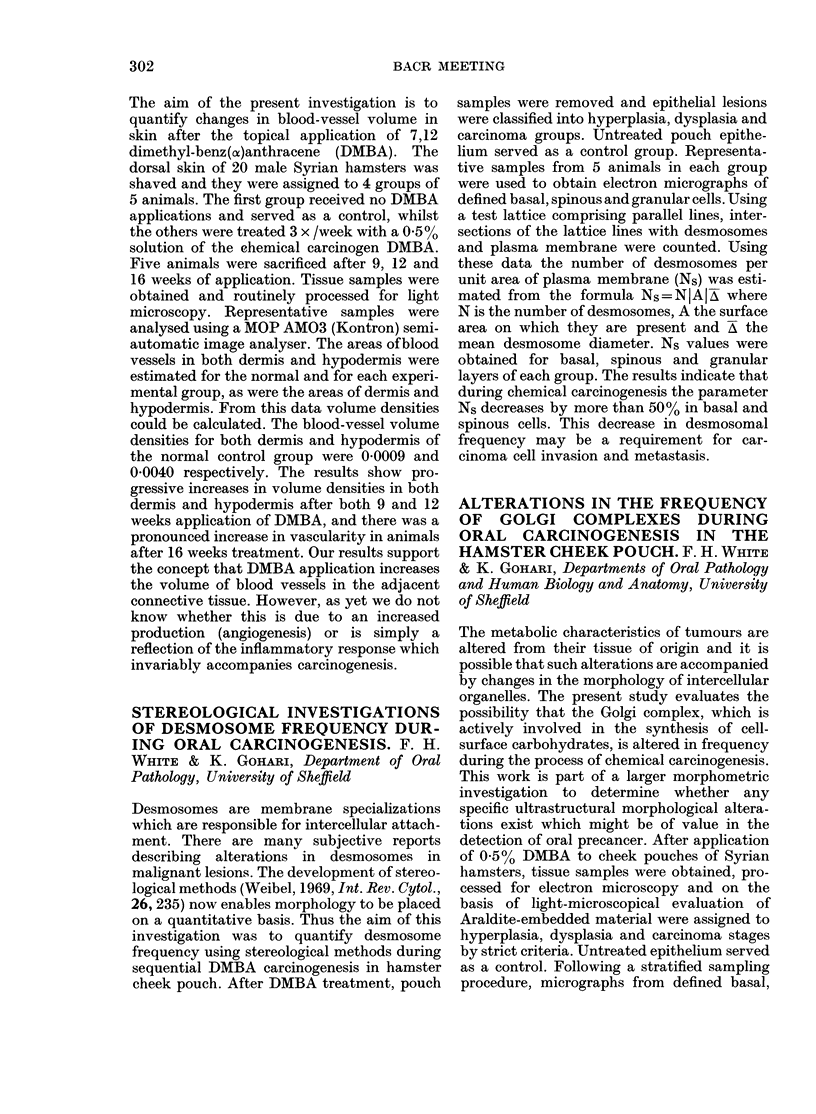

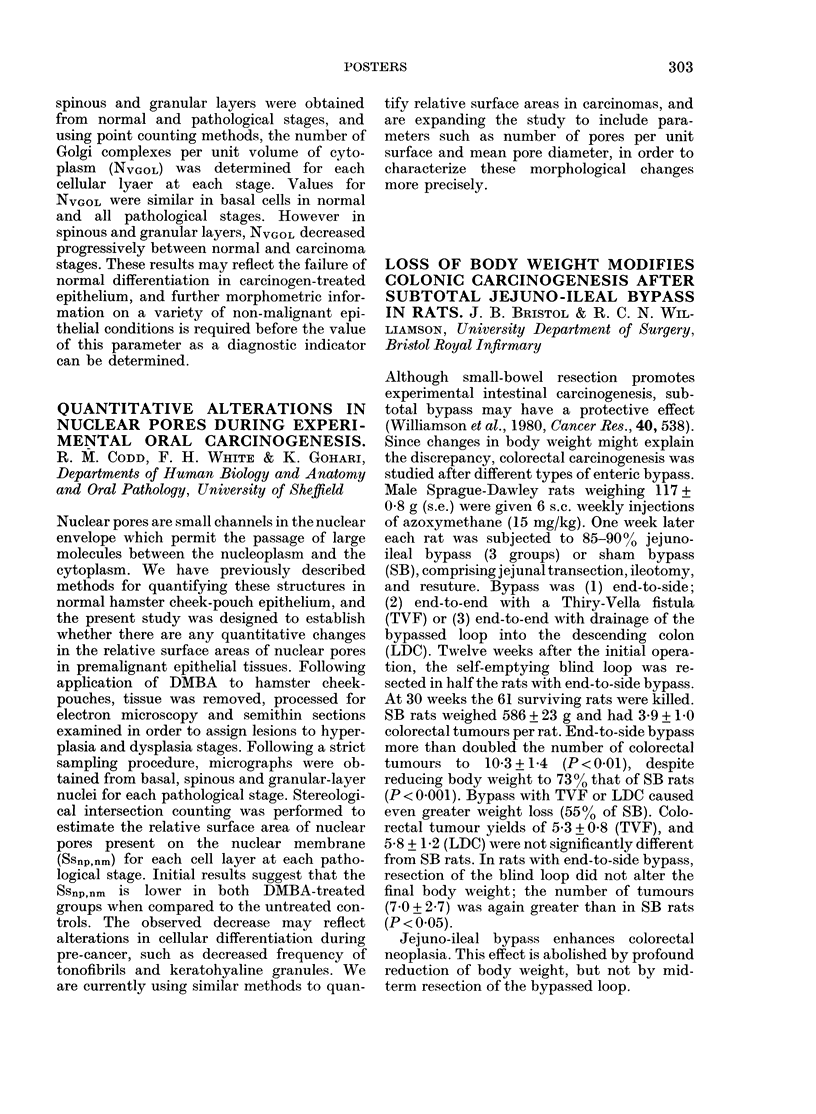

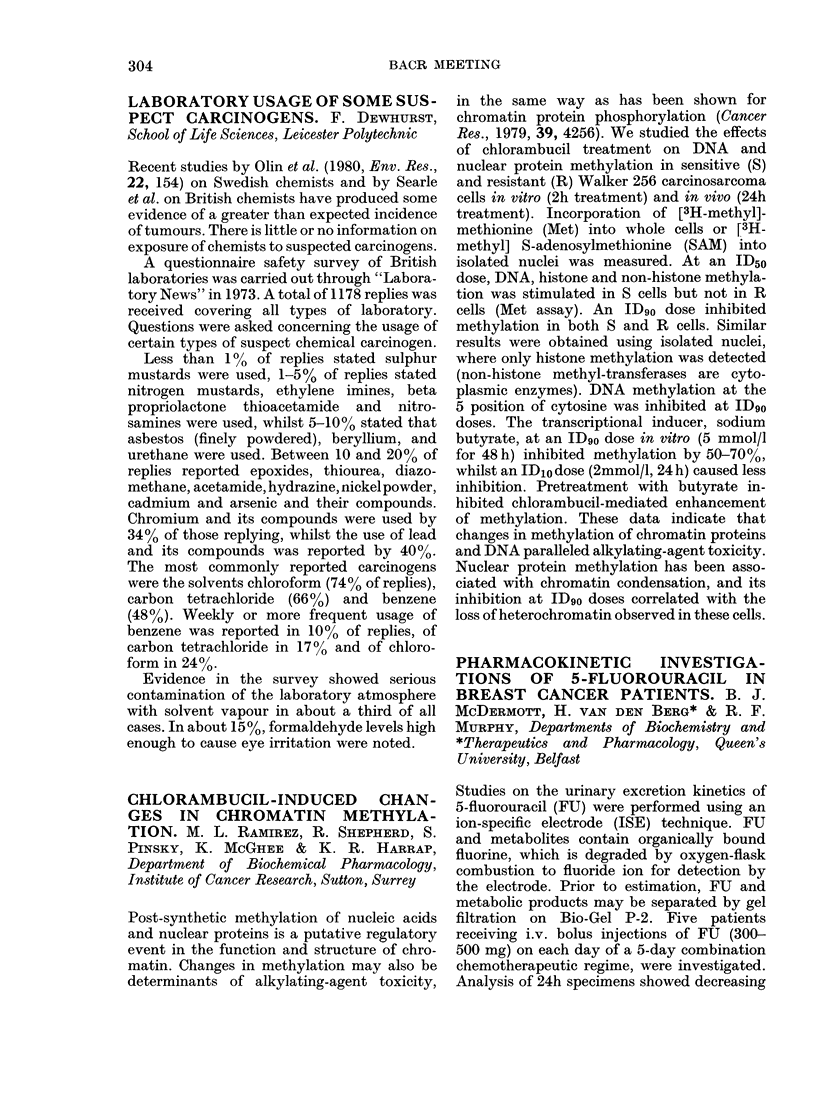

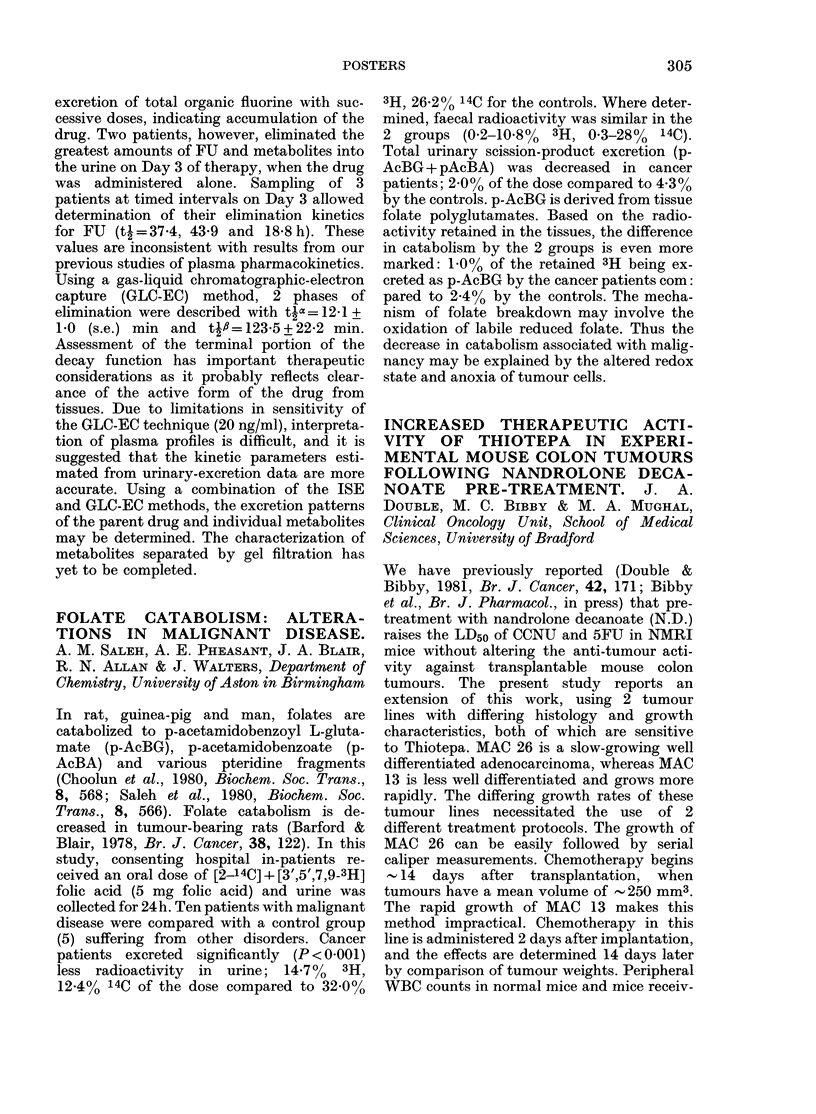

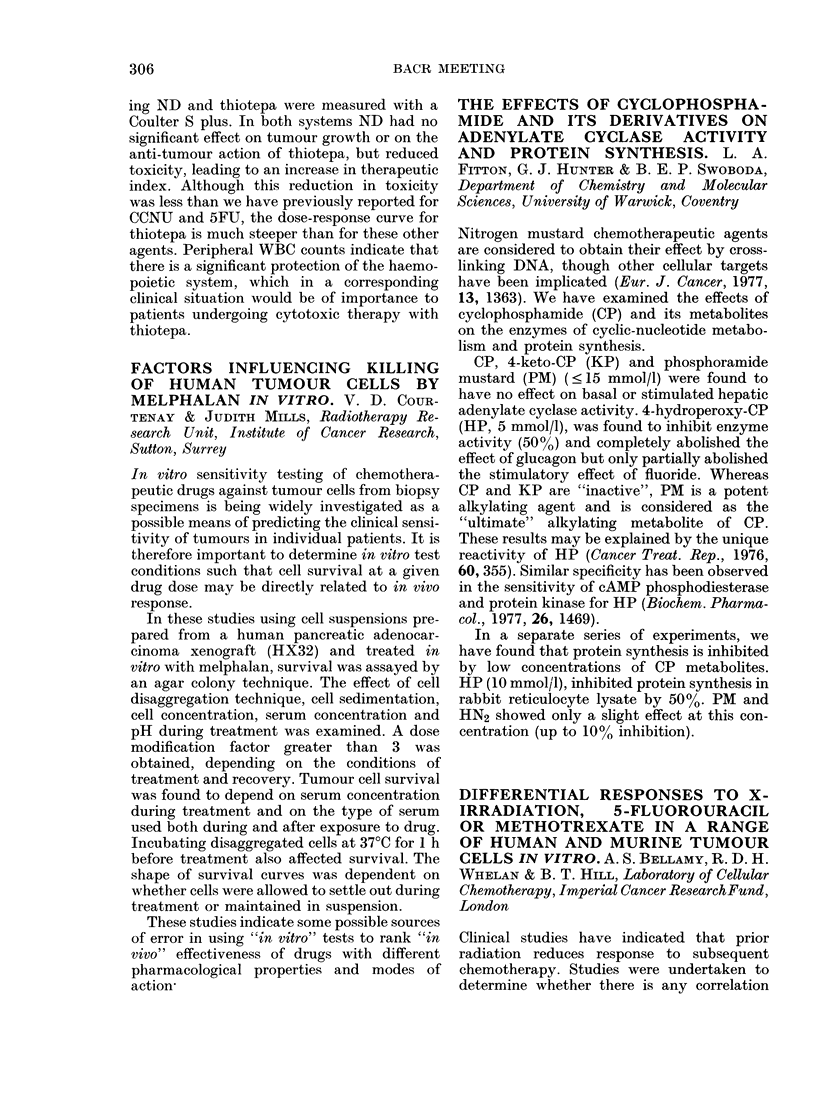

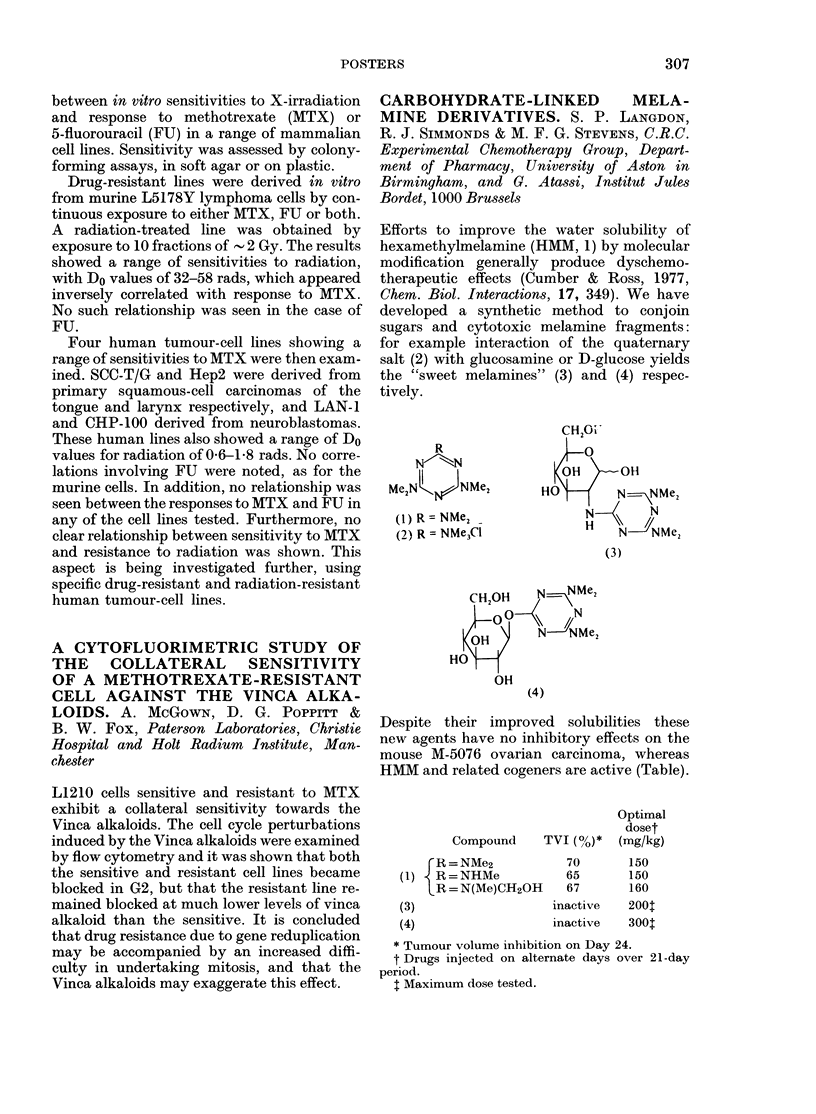

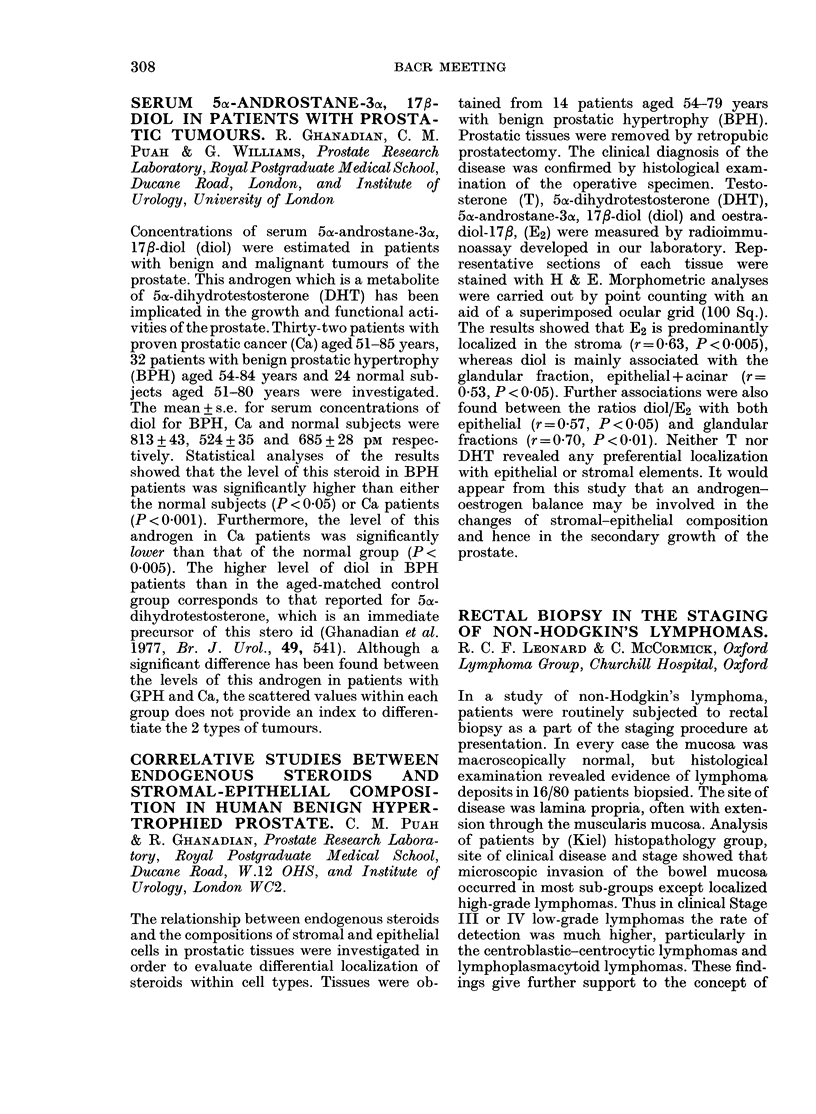

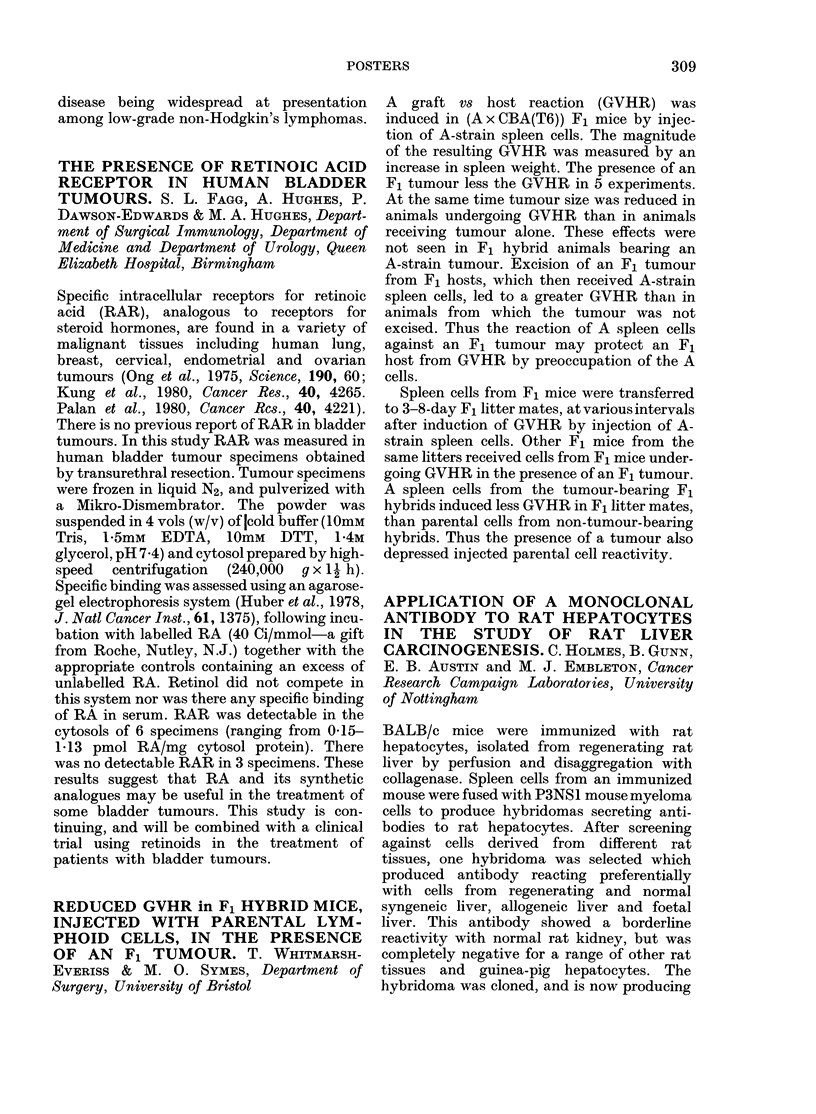

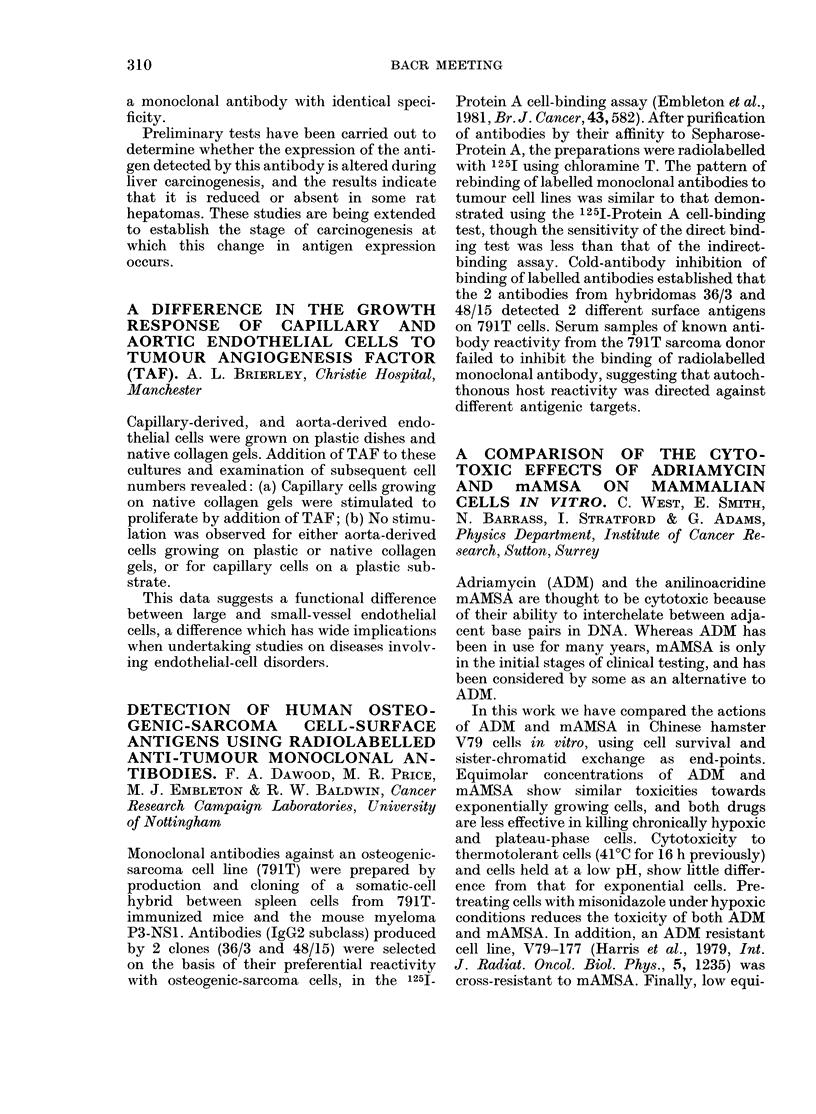

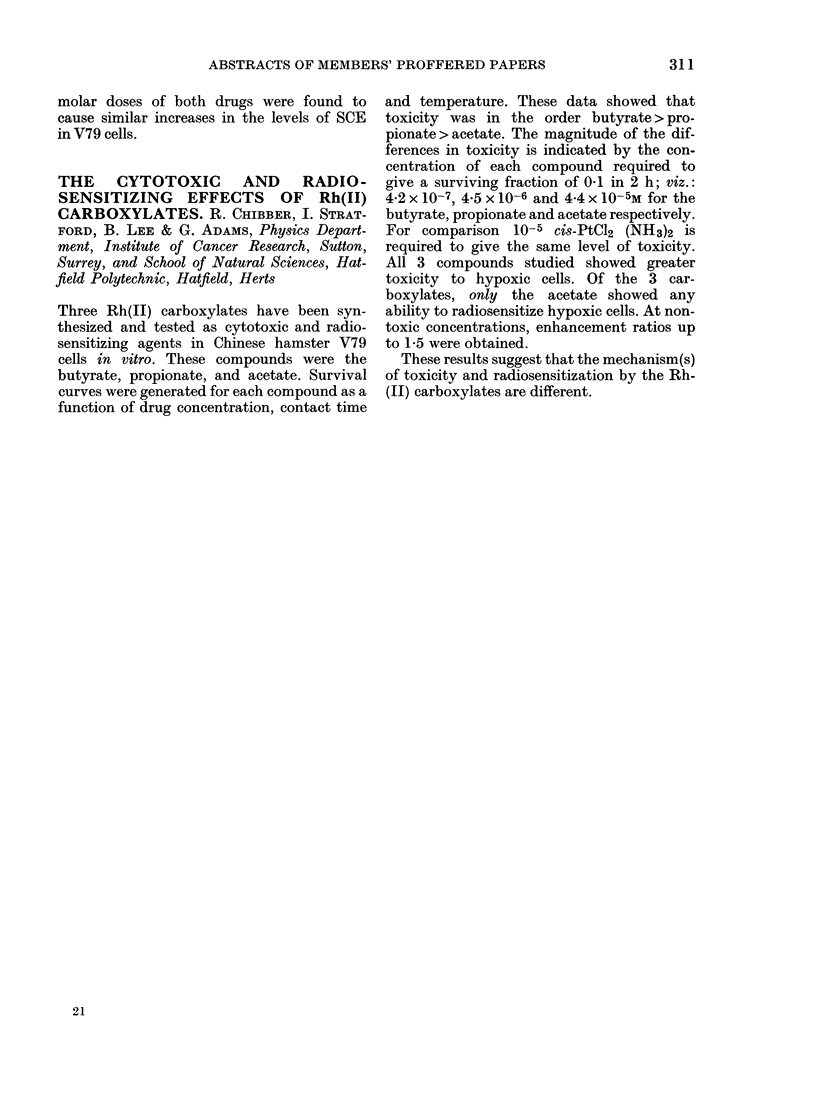


## References

[OCR_01247] Alberts D. S., Chang S. Y., Chen H. S., Larcom B. J., Jones S. E. (1979). Pharmacokinetics and metabolism of chlorambucil in man: a preliminary report.. Cancer Treat Rev.

[OCR_01253] Alberts D. S., Chen H. S., Mayersohn M., Perrier D., Moon T. E., Gross J. F. (1979). Bleomycin pharmacokinetics in man. II. Intracavitary administration.. Cancer Chemother Pharmacol.

[OCR_01260] Alberts D. S., Samon S. E., Chen H. S., Surwit E. A., Soehnlen B., Young L., Moon T. E. (1980). In-vitro clonogenic assay for predicting response of ovarian cancer to chemotherapy.. Lancet.

[OCR_03369] Allen R. C., Stjernholm R. L., Steele R. H. (1972). Evidence for the generation of an electronic excitation state(s) in human polymorphonuclear leukocytes and its participation in bactericidal activity.. Biochem Biophys Res Commun.

[OCR_00777] BURCHENAL J. H., KARNOFSKY D. A., MYERS W. P. L. (1956). The hepatotoxic action of N-methylformamide in man.. Cancer.

[OCR_01266] Barlow J. J., Piver M. S. (1977). Single agent vs combination chemotherapy in the treatment of ovarian cancer.. Obstet Gynecol.

[OCR_01271] Berry R. J., Laing A. H., Wells J. (1975). Fresh explant culture of human tumours in vitro and the assessment of sensitivity to cytotoxic chemotherapy.. Br J Cancer.

[OCR_01277] Bischoff K. B., Dedrick R. L., Zaharko D. S., Longstreth J. A. (1971). Methotrexate pharmacokinetics.. J Pharm Sci.

[OCR_02689] Carswell E. A., Old L. J., Kassel R. L., Green S., Fiore N., Williamson B. (1975). An endotoxin-induced serum factor that causes necrosis of tumors.. Proc Natl Acad Sci U S A.

[OCR_00238] Chu Y. H., Craig A. W., O'Connor P. J. (1981). Repair of O6-methylguanine in rat liver DNA is enhanced by pretreatment with single or multiple doses of aflatoxin B1.. Br J Cancer.

[OCR_01406] Cobb L. M., Connors T. A., Elson L. A., Khan A. H., Mitchley B. C., Ross W. C., Whisson M. E. (1969). 2,4-dinitro-5-ethyleneiminobenzamide (CB 1954): a potent and selective inhibitor of the growth of the Walker carcinoma 256.. Biochem Pharmacol.

[OCR_01287] Coffey J. J., White C. A., Lesk A. B., Rogers W. I., Serpick A. A. (1972). Effect of allopurinol on the pharmacokinetics of 6-mercaptopurine (NSC 755) in cancer patients.. Cancer Res.

[OCR_01293] Connors T. A., Cox P. J., Farmer P. B., Foster A. B., Jarman M. (1974). Some studies of the active intermediates formed in the microsomal metabolism of cyclophosphamide and isophosphamide.. Biochem Pharmacol.

[OCR_04331] Cumber A. J., Ross W. C. (1977). Analogues of hexamethylmelamine. The anti-neoplastic activity of derivatives with enhanced water solubility.. Chem Biol Interact.

[OCR_03061] Currie G. A., Basham C. (1972). Serum mediated inhibition of the immunological reactions of the patient to his own tumour: a possible role for circulating antigen.. Br J Cancer.

[OCR_01300] Dedrick R. L., Forrester D. D., Ho D. H. (1972). In vitro-in vivo correlation of drug metabolism--deamination of 1- -D-arabinofuranosylcytosine.. Biochem Pharmacol.

[OCR_01306] Dendy P. P., Bozman G., Wheeler T. K. (1970). In-vitro screening test for human malignant tumours before chemotherapy.. Lancet.

[OCR_03316] Edwards P. A., Easty D. M., Foster C. S. (1980). Selective culture of epithelioid cells from a human squamous carcinoma using a monoclonal antibody to kill fibroblasts.. Cell Biol Int Rep.

[OCR_01319] Finj C., Sadée W. (1975). Determination of 5-fluorouracil (NSC-19893) plasma levels in rats and man by isotope dilution-mass fragmentography.. Cancer Chemother Rep.

[OCR_01325] Freshney R. I., Paul J., Kane I. M. (1975). Assay of anti-cancer drugs in tissue culture: conditions affecting their ability to incorporate 3H-leucine after drug treatment.. Br J Cancer.

[OCR_02561] Galasko C. S., Sylvester B. S. (1978). Back pain in patients treated for malignant tumours.. Clin Oncol.

[OCR_04648] Harris J. R., Timberlake N., Henson P., Schimke P., Belli J. A. (1979). Adriamycin uptake in V79 and adriamycin resistant Chinese hamster cells.. Int J Radiat Oncol Biol Phys.

[OCR_01330] Harris P. A., Gross J. F. (1975). Preliminary pharmacokinetic model for adriamycin (NSC-123127).. Cancer Chemother Rep.

[OCR_03243] Heyderman E., Neville A. M. (1977). A shorter immunoperoxidase technique for the demonstration of carcinoembryonic antigen and other cell products.. J Clin Pathol.

[OCR_01340] Holmes H. L., Little J. M. (1974). Tissue-culture microtest for predicting response of human cancer to chemotherapy.. Lancet.

[OCR_04586] Huber P. R., Geyer E., Küng W., Matter A., Torhorst J., Eppenberger U. (1978). Retinoic acid-binding protein in human breast cancer and dysplasia.. J Natl Cancer Inst.

[OCR_01345] Ioachim H. L., Sabbath M., Andersson B., Barber H. R. (1974). Tissue cultures of ovarian carcinomas.. Lab Invest.

[OCR_00499] Kinsella A. R., Radman M. (1978). Tumor promoter induces sister chromatid exchanges: relevance to mechanisms of carcinogenesis.. Proc Natl Acad Sci U S A.

[OCR_01354] Lieberman A., LeBrun Y., Glass P., Goodgold A., Lux W., Wise A., Ransohoff J. (1977). Use of high dose corticosteroids in patients with inoperable brain tumours.. J Neurol Neurosurg Psychiatry.

[OCR_01360] Limburg H., Heckmann U. (1968). Chemotherapy in the treatment of advanced pelvic malignant disease with special reference to ovarian cancer.. J Obstet Gynaecol Br Commonw.

[OCR_01366] Malkasian G. D., Decker D. G., Jorgensen E. O., Edmonson J. H. (1977). Medroxyprogesterone acetate for the treatment of metastatic and recurrent ovarian carcinoma.. Cancer Treat Rep.

[OCR_02415] Meares A. (1979). Meditation: a psychological approach to cancer treatment.. Practitioner.

[OCR_01015] Millar J. L., McElwain T. J. (1978). Combinations of cytotoxic agents that have less than expected toxicity on normal tissues in mice.. Antibiot Chemother (1971).

[OCR_03804] Olin G. R., Ahlbom A. (1980). The cancer mortality among Swedish chemists graduated during three decades. A comparison with the general population and with a cohort of architects.. Environ Res.

[OCR_00756] Olson R. D., MacDonald J. S., vanBoxtel C. J., Boerth R. C., Harbison R. D., Slonim A. E., Freeman R. W., Oates J. A. (1980). Regulatory role of glutathione and soluble sulfhydryl groups in the toxicity of adriamycin.. J Pharmacol Exp Ther.

[OCR_01373] Owellen R. J., Hartke C. A., Hains F. O. (1977). Pharmacokinetics and metabolism of vinblastine in humans.. Cancer Res.

[OCR_01378] Owellen R. J., Root M. A., Hains F. O. (1977). Pharmacokinetics of vindesine and vincristine in humans.. Cancer Res.

[OCR_04442] Palan P. R., Romney S. L. (1980). Cellular binding proteins for vitamin A in human carcinomas and in normal tissues.. Cancer Res.

[OCR_01383] Patton T. F., Himmelstein K. J., Belt R., Bannister S. J., Sternson L. A., Repta A. J. (1978). Plasma levels and urinary excretion of filterable platinum species following bolus injection and iv infusion of cis-dichlorodiammineplatinum(II) in man.. Cancer Treat Rep.

[OCR_00352] Pegg A. E., Hui G. (1978). Formation and subsequent removal of O6-methylguanine from deoxyribonucleic acid in rat liver and kidney after small doses of dimethylnitrosamine.. Biochem J.

[OCR_01392] Rutty C. J., Connors T. A. (1977). In vitro studies with hexamethylmelamine.. Biochem Pharmacol.

[OCR_01397] Rutty C. J., Connors T. A., Nguyen-Hoang-Nam, Do-Cao-Thang, Hoellinger H. (1978). In vivo studies with hexamethylmelamine.. Eur J Cancer.

[OCR_03960] Saleh A. M., Pheasant A. E., Blair J. A., Allan R. N. (1980). The effect of malignant disease on the metabolism of pteroylglutamic acid in man.. Biochem Soc Trans.

[OCR_01404] Schabel F. M., Trader M. W., Laster W. R., Wheeler G. P., Witt M. H. (1978). Patterns of resistance and therapeutic synergism among alkylating agents.. Antibiot Chemother (1971).

[OCR_01105] Shorthouse A. J., Smyth J. F., Steel G. G., Ellison M., Mills J., Peckham M. J. (1980). The human tumour xenograft--a valid model in experimental chemotherapy?. Br J Surg.

[OCR_03393] Sundström C., Nilsson K. (1976). Establishment and characterization of a human histiocytic lymphoma cell line (U-937).. Int J Cancer.

[OCR_01411] Tattersall M. H., Sodergren J. E., Dengupta S. K., Trites D. H., Modest E. J., Frei E. (1975). Pharmacokinetics of actinoymcin D in patients with malignant melanoma.. Clin Pharmacol Ther.

[OCR_02462] Theologides A. (1979). Cancer cachexia.. Cancer.

[OCR_00866] Tisdale M. J. (1980). Effect of methionine replacement by homocysteine on the growth of cells.. Cell Biol Int Rep.

[OCR_01433] WRIGHT J. C., COBB J. P., GUMPORT S. L., GOLOMB F. M., SAFADI D. (1957). Investigation of the relation between clinical and tissue-culture response to chemotherapeutic agents on human cancer.. N Engl J Med.

[OCR_03748] Weibel E. R. (1969). Stereological principles for morphometry in electron microscopic cytology.. Int Rev Cytol.

[OCR_01416] Wheeler T. K., Dendy P. P., Dawson A. (1974). Assessment of an in vitro screening test of cytotoxic agents in the treatment of advanced malignant disease.. Oncology.

[OCR_01422] Whiting B., Miller S. H., Caddy B. (1978). A procedure for monitoring cyclophosphamide and isophosphamide in biological samples.. Br J Clin Pharmacol.

